# Immunogenicity and protective efficacy on non-adjuvanted CD40-targeting SARS-CoV-2 vaccines in non-human primates

**DOI:** 10.1016/j.ebiom.2026.106361

**Published:** 2026-07-01

**Authors:** Romain Marlin, Mireille Centlivre, Laetitia Bossevot, Anne-Sophie Gallouët, Marie Alexandre, Delphine Planas, Mariangela Cavarelli, Isabelle Staropoli, Wesley Gros, Mathieu Surenaud, Craig Fenwick, Sylvain Cardinaud, Mathilde Galhaut, Sandra Zurawski, Victor Magneron, Loïc Pintore, Cécile Hérate, Marie-Anne Rameix Welti, Paul Mazet, Vanessa Contreras, Francis Relouzat, Nathalie Dereuddre-Bosquet, Giuseppe Pantaleo, Rodolphe Thiébaut, Mélanie Prague, Olivier Schwartz, Gerard Zurawski, Véronique Godot, Roger Le Grand, Yves Lévy

**Affiliations:** a Université Paris-Saclay, Inserm, CEA; Immune Diseases, Microbiology and Innovative Therapies (IDMIT/UMRS1184); Fontenay-aux-Roses & Le Kremlin-Bicêtre, France; b Vaccine Research Institute, Créteil, France; c Inserm U955, Equipe 16, Institut Mondor de Recherche Biomédicale, Université Paris-Est Créteil, Créteil, France; d Université de Bordeaux, Inserm U1219 Bordeaux Population Health Research Centre, Inria SISTM, 33000, Bordeaux, France; e Virus and Immunity Unit, Institut Pasteur, Université Paris Cité, Paris, France; f Service of Immunology and Allergy, Lausanne University Hospital (CHUV), Lausanne, Switzerland; g Baylor Scott and White Research Institute and Inserm U955, Dallas, TX, United States of America; h National Reference Center for Respiratory Viruses, Molecular Mechanisms of Multiplication of Pneumoviruses Unit, Institut Pasteur, Université Paris Cité, Paris, France; i Molecular Mechanisms of Multiplication of Pneumoviruses, Université Paris-Saclay, Université de Versailles St. Quentin, UMR 1173 (2I), INSERM, Assistance Publique des Hôpitaux de Paris, Paris, France; j Swiss Vaccine Research Institute, Lausanne University Hospital, University of Lausanne, Lausanne, Switzerland; k CHU Bordeaux, Department of Medical information, Bordeaux, France; l AP-HP, Hôpital Henri-Mondor Albert-Chenevier, Service d'Immunologie Clinique et Maladies Infectieuses, Créteil, France

**Keywords:** Preclinical study, Immunogenicity, SARS-CoV-2 vaccine candidate, Non-human primate, Dendritic cell targeting vaccine, CD40

## Abstract

**Background:**

The emergence of antigenically distinct SARS-CoV-2 variants increases the risk of immune escape and requires continually updated vaccines. In addition, short-lived specific immunity is a limitation faced by current COVID-19 mRNA vaccines against Sarbecoviruses. This underscores the need for new vaccine approaches providing lasting immunity against SARS-CoV-2.

**Methods:**

Here, we demonstrate the capacity of two non-adjuvanted subunit vaccines to induce long-lasting and protective immunity against SARS-CoV-2 variants in macaques. We designed antibody-mediated vaccines (AMV) leveraging an anti-CD40 monoclonal antibody to enhance immune responses by targeting selected antigens to antigen-presenting cells through the CD40 receptor. The CD40.RBDv vaccine targets sequences from the original Wuhan RBD and a mutated RBD, while CD40.Pan.CoV incorporates a conserved nucleocapsid sequence and a mutated RBD region.

**Findings:**

We show that both adjuvant-free vaccines induce robust and durable systemic and mucosal anti-RBD antibody responses that neutralise multiple SARS-CoV-2 variants in naive and SARS-CoV-2 convalescent animals, including recent variants such as XFG. In convalescents, mathematical modelling predicted persistence of vaccine-induced antibody for decades. Additionally, the vaccines boost immune responses in mRNA-vaccinated animals, demonstrating the efficiency of CD40-based vaccines as boosters. Both vaccines protect animals against B.1.617.2 Delta and BA.1 Omicron challenges. Viral control correlates with vaccine-induced systemic and mucosal antibody levels and T-cell responses.

**Interpretation:**

These findings support the ability of AMV targeting CD40 to induce strong, long-lasting responses against adapted antigens and broad protection against evolving SARS-CoV-2 variants without requiring adjuvant. These two vaccine candidates are currently under clinical testing.

**Funding:**

The Investissements d’Avenir program (ANR-10-LABX-77-01 and ANR-11-INBS-0008), the PSPC COVID-19 – Project EVIDENCE.

**Research in context**

**Evidence before this study**

We searched major biomedical literature databases to identify relevant preclinical and clinical studies on SARS-CoV-2 vaccine immunogenicity, durability and protective efficacy, with a particular focus on protein subunit and dendritic cell-targeted vaccine strategies. The search covered the period between September 1, 2021 and December 1, 2025, using combinations of the terms “SARS-CoV-2 vaccine”, “protein subunit vaccine”, “DC-targeting vaccine”, “mRNA booster”, “durability of immune response” and “hybrid immunity”. Previous studies demonstrated that first-generation mRNA vaccines effectively reduced COVID-19 morbidity and mortality, but available evidence consistently showed limited durability of protection against infection, particularly with the emergence of immune-evading variants such as Omicron subvariants. Subunit vaccines demonstrated favourable safety, tolerability, and scalability, but often required multiple doses and adjuvant formulations to achieve sufficient immunogenicity and optimal efficacy. Preclinical research increasingly supported the targeting of dendritic cells, particularly through CD40 engagement, as a beneficial strategy for amplifying vaccine-induced immunity. Early clinical studies of CD40-targeted vaccines against human immunodeficiency virus (HIV) and human papillomavirus (HPV) confirmed feasibility, safety, and immunogenicity, while preclinical studies indicated protective efficacy of CD40-targeted constructs against SARS-CoV-2. However, limited data were available regarding the durability, breadth and protective efficacy of CD40-targeted vaccines against SARS-CoV-2 variants of concern (VOCs), particularly in the context of hybrid immunity and boosting following mRNA vaccination.

**Added value of this study**

This study provides a comprehensive preclinical evaluation of two antibody-mediated targeting vaccine candidates in non-human primates. One candidate was designed to present two receptor-binding domain (RBD) sequences. The second candidate combines a highly conserved Nucleocapsid sequence with an RBD sequence to broaden the applicability of SARS-CoV-2 vaccines beyond the Spike protein. Preclinical evaluation of these two adjuvant-free CD40-targeted vaccine candidates demonstrated their ability to elicit robust systemic and mucosal antibody responses, and induce durable immunity. The vaccines were immunogenic both as primary immunisation in naïve animals and as heterologous boosters in animals with pre-existing natural or mRNA vaccine-induced immunity. A single non-adjuvanted CD40 vaccine boost generated durable antibody responses with predicted persistence exceeding that observed after mRNA vaccination alone. Protective efficacy was supported by reduced viral replication following VOC challenge and by the absence of detectable subgenomic viral RNA in vaccinated animals. This study also identifies immune correlates associated with protection and highlights the potential of CD40-targeted strategies to improve the breadth and durability of SARS-CoV-2 vaccine-induced immunity without the need for adjuvant formulations.

**Implications of all the available evidence**

Taken together with existing evidence, our findings support CD40-targeted vaccines as a potential strategy for next-generation SARS-CoV-2 vaccines aimed at improving the durability and breadth of protective immunity against emerging variants. The ability of non-adjuvanted CD40 vaccines to induce potent immune responses in both naïve and previously immunised hosts suggests potential utility as heterologous booster vaccines in populations with pre-existing immunity.

More broadly, these findings reinforce the relevance of dendritic cell-targeted vaccine approaches for enhancing long-term antiviral immunity and support further clinical development of CD40-targeted vaccine platforms. Such strategies may contribute to improved protection against evolving SARS-CoV-2 variants and could have broader applications for vaccines against other viruses.

## Introduction

The rapid development of SARS-CoV-2 vaccines in 2020 marked an unprecedented advancement in science and public health, significantly reducing COVID-19 mortality and morbidity. While initial vaccine formulations and boosters effectively mitigated severe illness, hospitalisation, and death, they did not fully prevent infection nor transmission. The ongoing emergence of SARS-CoV-2 variants of interest (VOIs) and variants of concern (VOCs) continue to pose a public health challenge.

Current vaccines primarily target the SARS-CoV-2 Spike (S) protein, which is the main site recognised by neutralising antibodies. Many of these potent neutralising antibodies bind to the receptor-binding domain (RBD) of the S protein, often competing with its binding to the ACE2 receptor. Emerging VOCs frequently exhibit immune escape mutations within the S and RBD sequences. The extensive mutations observed in the SARS-CoV-2 Omicron variant and its sub-lineages, compared to previous VOCs, are particularly concerning. This rapid viral evolution has progressively eroded vaccine-induced immunity, increasing the risk of infection with each new variant wave. While the precise evolutionary trajectory of the pandemic remains uncertain, the emergence of antigenically distinct VOCs presents a substantial obstacle to achieving herd immunity and maintaining strong vaccine effectiveness.

Optimal pandemic control necessitates vaccines that demonstrate high efficacy, broad protection, and ideally, long-lasting immunity. The observed decline in neutralising antibody levels associated with protection against symptomatic COVID-19^1^ following mRNA vaccination raises concerns and necessitates booster vaccinations with updated compositions.^2^ Moreover, the need for repeated vaccinations may lead to decreased public adherence and vaccine fatigue. Therefore, the lack of durability of vaccine-induced protective immunity against infection, coupled with the continuous emergence of immune-evading SARS-CoV-2 variants, represents a significant challenge underscoring the need for innovative vaccine strategies.

Licenced subunit protein vaccines have established safety and tolerability profiles across diverse populations.^3^^,^^4^ These vaccines offer advantages such as low distribution costs, the absence of cold-chain requirements, and high scalability. Subunit vaccine often requires immunogenicity enhancement through adjuvant formulations. Numerous SARS-CoV-2 Spike protein-based subunit vaccines with various formulations and adjuvants are currently under investigation.^5^ However, the multitude of potential adjuvant combinations adds complexity and may limit development or impact tolerability.

Enhancing protein immunogenicity can be achieved by improving delivery to antigen-presenting cells (APCs), particularly dendritic cells (DCs), which are crucial for initiating and regulating antigen-specific immunity. DCs capture, process, and present antigens to T cells as peptides bound to MHC class I and II molecules.^6^, ^7^, ^8^, ^9^ Targeting DC-specific receptors, such as MHC class II,^10^ C-type lectin-like receptors,^11^ or TNF family members like CD40,^12^ has demonstrated significant enhancement of immune responses to vaccine antigens. Our group has developed an antibody-mediated targeting vaccine (AMV) platform, utilising a fully humanised monoclonal antibody for targeting selected pathogen epitopes to the CD40 receptor, thereby enhancing antigen delivery and APC stimulation.^13^^,^^14^ We have reported the safety and immunogenicity of CD40-targeted HIV and HPV16 vaccines in non-HIV-infected volunteers (NCT04842682) and patients with HPV16-induced head and neck cancer (NCT06007092 EuCT N°: 2022-502930-25-00), respectively.^15^^,^^16^ Preclinical studies have also demonstrated the efficacy of a first-generation CD40-targeting vaccine in controlling SARS-CoV-2 infection.^17^, ^18^, ^19^

This study evaluates two CD40 AMV candidates in non-human primates (NHP), assessing immunogenicity and efficacy against SARS-CoV-2 VOCs challenges (B.1.617.2 Delta and Omicron BA.1). Leveraging the flexibility of this technology, we designed a bivalent CD40 construct presenting both the RBD from the original Wuhan strain and a RBD variant incorporating K417N, E484K, and N501Y mutations (RBDv1). To broaden the scope of SARS-CoV-2 vaccines beyond the S protein, a second candidate, CD40.Pan.CoV, includes a highly conserved non-S Nucleocapsid sequence (Npep2; 93.5% across Sarbecoviruses) and a RBD sequence harbouring K417N, L452R, T478K, E484Q, and N501Y mutations (RBDv2). We investigated the potency of these two vaccines, administered without adjuvant, both as boosters to pre-existing natural or mRNA vaccine-induced immunity and as a primary immunisation in naive animals. Finally, we assessed humoural and cellular immune responses associated with protection following Delta and Omicron BA.1 challenge.

## Methods

### Design and production of CD40.RBDv, IgG4.RBDv and CD40.Pan.CoV vaccines

Methods for expression vectors for antibody production and purification via transient transfection into Expi-CHO cells (ThermoFisher Scientific) with TransIT-PRO Pro reagent (Mirus Bio) using the manufacturers protocol and quality assurance including CD40 binding specificity were as previously described.^20^, ^21^, ^22^ The bivalent (two-subunit) CD40.RBDv vaccine was humanised anti-human CD40 12E12 antibody H chain (GenPept sequence ID: AJD85779.1 residues 20–467) fused at the C-terminus to SARS-CoV-2 Wuhan strain Spike protein S1 RBD (GenPept sequence ID: 7VYR_C residues 40–262 with a C259S replacement). To extend the landscape of COVID-19 vaccines based on the use of the SARS-CoV-2 S antigen this was paired to humanised anti-human CD40 12E12 antibody L chain (GenPept sequence ID: AJD85780.1 residues 21–236) fused at the C-terminus to SARS-CoV-2 Spike protein S1 RBDv1 with K417N, E584K and N501Y mutations (GenPept sequence ID: 7S5P_A residues 1–223 with a C220S replacement) described in VOC Alpha (N501Y), Beta (K417N, E484K, N501Y), Gamma (E484K, N501Y), Omicron BA.1, BA.2, BA.4, BA.5, BQ.1 or BQ.1.1 (K417N, N501Y) (Figure S1a). The CD40 non-binding human IgG4 control RBDv vaccine was as above except the antibody H chain was GenPept sequence ID: AIC59040.1 residues 27–473 with appended AS and the antibody L chain was GenPept sequence ID: BAC01726.1 residues 23–236 with appended AS.

The second candidate CD40.Pan.CoV vaccine was humanised anti-human CD40 12E12 antibody H chain (GenPept sequence ID: AJD85779.1 residues 20–467) fused at the C-terminus via a flexible linker sequence f4 (GenPept sequence ID: AJD85777.1 residues 699–725) to SARS-CoV-2 nucleocapsid phosphoprotein Npep2 (GenPept sequence ID: UHS04165.1 residues 95–230 with appended AS) paired to humanised anti-human CD40 12E12 antibody L chain (GenPept sequence ID: AJD85780.1 residues 21–236) fused at the C-terminus to SARS-CoV-2 Spike protein S1 RBDv2 with K417N, L452R, T478K, E484Q, N501Y mutations (GenPept sequence ID: 7S5P_A residues 1–223 with L134R, T160K, K166Q and C220S replacements) described in VOC Alpha (N501Y), Beta (K417N, N501Y), Gamma (N501Y), Delta (L452R, T478K), Kappa (L452R, E484Q), Omicron BA.1/BA.2 (K417N, T478K, N501Y), Omicron BA.4/BA.5/BQ.1/BQ.1.1 (K417N, L452R, T478K, N501Y) (Figure S1b). In all cases codons were optimised based on CHO usage. The Npep2 sequence is highly conserved (mean 93.5% [min 89.7 to max 100%]) across Sarbecoviruses and was selected through the alignment of sequences from SARS-CoV-2, VOCs, SARS and 32 described SARS-CoV-related coronaviruses (all from the Sarbecovirus subgenus).^18^ The T and B cell epitope enrichment of the Npep2 sequence has been already described.^18^

### Ethics and biosafety statement animal studies

Cynomolgus macaques (*Macaca fascicularis*), aged 54 months (median; [IQR 38–59 months]) at the initiation of immunisations (48 females and 55 males) and originating from Mauritian AAALAC certified breeding centers were used in this study. All animals were housed in IDMIT facilities (CEA, Fontenay-aux-Roses), under BSL-2 or BSL-3 containments (Animal facility authorisation #D92-032-02, Préfecture des Hauts de Seine, France) and in compliance with European Directive 2010/63/EU, the French regulations and the Standards for Human Care and Use of Laboratory Animals, of the Office for Laboratory Animal Welfare (OLAW, assurance number #A5826-01 and #F22-00556, US). The protocols were approved by the institutional ethical committee “Comité d’Ethique en Expérimentation Animale du Commissariat à l’Energie Atomique et aux Energies Alternatives” (CEtEA #44) under statement numbers A20-061, A20-066 and A22-006. The study was authorised by the “Research, Innovation and Education Ministry” under registration numbers APAFIS#28946-2021011312169043 v2, APAFIS#29191-2021011811505374 v1 and APAFIS #36939-2022042217237124 v1.

### Non-human primate study design

A first analysis was performed on SARS-CoV-2 naive animals: Cynomolgus macaques were injected subcutaneously (SC): i) on week 0 with 200 μg of CD40.RBDv (n = 6); ii) for durability analysis, on week 0 with 200 μg of CD40.RBDv (n = 2) or non-targeting vaccine control IgG4.RBDv (n = 3) and; iii) on weeks 0, 6 and 12 with 200 μg of CD40.Pan.CoV (n = 6). The SC route of administration was chosen due to the mode of action of CD40-targeted vaccines. This platform directs the antigens to dermal dendritic cells and antigen-presenting cells expressing CD40 receptor on their surface. The 200 μg dose was selected based on previous preclinical studies of other CD40-targeted vaccines, which demonstrated favourable profiles in terms of local reactogenicity and systemic immunogenicity. Moreover, safety analyses were performed here on naive animals with a manufacturing formulation of CD40.Pan.CoV. Macaques were injected SC on weeks 0 and 6 with either 1 mg of CD40.Pan.CoV (n = 2) or 1 mg of CD40.Pan.CoV adjuvanted with 1 mg of Poly-ICLC (Hiltonol®, Oncovir) (n = 2).

A second analysis was performed on convalescent animals that had been infected 77 weeks before (median; [IQR 74–77 weeks]) with SARS-CoV-2 Wuhan strain. SARS-CoV-2 (Wuhan) convalescent cynomolgus macaques were injected SC on week 0 with 200 μg of CD40.RBDv (n = 6) or CD40.Pan.CoV (n = 5), or intramuscularly (IM) on week 0 with 30 μg of BNT162b2 mRNA vaccine (Pfizer-BioNTech). Non-vaccinated Wuhan SARS-CoV-2 convalescent macaques (n = 6) and non-vaccinated SARS-CoV-2-naive macaques (n = 7) were included as controls. For durability analysis, SARS-CoV-2 convalescent cynomolgus macaques were injected SC on week 0 with 200 μg of CD40.RBDv (n = 2) or on week 0 and 6 with 200 μg of CD40.Pan.CoV (n = 8) and one-year follow-up was performed on these animals. A long-term analysis was also conducted on SARS-CoV-2 convalescent NHPs previously infected by different strains. Omicron XBB.1.5 convalescent animals (infected 48 weeks before; [IQR 47–52 weeks]) received a SC injection at week 0 of either 200 μg of CD40.Pan.CoV alone (n = 4) or 200 μg of CD40.Pan.CoV adjuvanted with 1 mg of Poly-ICLC (n = 4). Non-vaccinated Omicron XBB.1.5 convalescent macaques (n = 3) were included as controls. Delta convalescent animals (infected 127 weeks before; [IQR 127–131 weeks]) received 200 μg of CD40.Pan.CoV alone (n = 4). Omicron BA.2 convalescent animals (infected 47 weeks before) received 30 μg of bivalent BNT162b2 mRNA vaccine (Wuhan/Omicron BA.4-5) IM at week 0.

Finally, immunogenicity of CD40 boost vaccine was analysed on non-convalescent mRNA immunised animals. Cynomolgus macaques (n = 12) were injected IM at weeks 0 and 4 with 30 μg of BNT162b2 mRNA vaccine. At week 21, animals were injected SC with 200 μg of CD40.RBDv (n = 6) or injected IM with 30 μg of BNT162b2 mRNA vaccine (n = 6). Non-vaccinated macaques (n = 11) were included as controls.

Efficacy of CD40.RBDv and CD40.Pan.CoV was analysed in Wuhan SARS-CoV-2 convalescent and mRNA immunised groups after SARS-CoV-2 (Delta or Omicron BA.1) viral exposure performed 4 weeks after the last vaccine injection. Both female and male cynomolgus macaques were included in the experimental groups. Sex was taken into account during study design, as our previous work did not identify sex-related differences in SARS-CoV-2 susceptibility or CD40 vaccine responses.

### Multiplex ELISA for evaluation of anti-SARS-CoV-2 antibody responses

Serum samples from cynomolgus macaques were analysed using electroluminescence (ECL) assays for RBD-specific and NCAP-specific IgG responses, as well as their neutralising activity (using a surrogate RBD-ACE2 binding inhibition assay). The analysis was performed against SARS-CoV-2 Wuhan and multiple VOCs using the V-PLEX SARS-CoV-2 Panels 2, 11 and Key Variant RBD Panel 1 (IgG and ACE2, MesoScale Discovery [MSD], Rockville, USA) following the manufacturer's instructions and as previously described.^23^ BAL and nasal samples from cynomolgus macaques were analysed for RBD-specific IgG and IgA responses. The analysis was performed against SARS-CoV-2 Wuhan and multiple VOCs using the V-PLEX SARS-CoV-2 Panel 11 following the manufacturer's instructions.

The plates were blocked with 150 μL of blocker A (1% BSA in MilliQ water) solution for at least 30 min at room temperature shaking at 700 rpm with a digital microplate shaker. During blocking, heat-inactivated serum samples were diluted 1:500 and 1:500,000 (IgG assay) or 1:10 and 1:100 (ACE2 assay) in diluent 100 buffer. Each plate contained duplicates of a seven-point calibration curve with serial dilution of a reference standard and a blank well. The plates were then washed three times with 150 μL of the MSD wash buffer, blotted dry, and 50 μL (IgG assay) or 25 μL (ACE2 assay) of the diluted samples were added to the plates and set to shake at 700 rpm at room temperature for 2 h for IgG assay and 1 h for ACE2 assay. For IgG assay, the plates were again washed three times and 50 μL of SULFO-Tagged anti-human IgG antibody was added to each well or for ACE2 assay, without plate washing, 25 μL of SULFO-Tagged human ACE2 protein was added to each well and all plates were incubated, shaking at 700 rpm at room temperature for at least 1 h. Plates were then washed three times, and 150 μL of MSD GOLD Read Buffer B was added to each well. The plates were read immediately after on a MESO QuickPlex SQ 120 machine. ECL signal was recorded. Results were expressed in AU/mL for total IgG and neutralising capacity for RBD Wuhan, Alpha, Beta and Delta. Antibody neutralising capacity against Omicron subvariants was expressed as % inhibition (% inhibition = 100 - ((sample ECL signal/ECL signal of ‘diluent only’) x 100)).

Positivity cutoff was established using about 50 sera from naive and SARS-CoV-2 infected cynomolgus from our facility. Nasal and BAL samples form ten naïve, uninfected control cynomolgus were used to determine the median reference value, which is represented in the figures by a dotted horizontal line.

### Antigen-specific T cell assays using non-human primate cells

To analyse the SARS-CoV-2 protein-specific T cell using functional assay, 15-mer peptides overlapping by 11 amino acids (aa) and covering the RBD Wuhan sequence (n = 53, aa 319–541 from Spike) and the SARS-CoV-2 Nucleoprotein sequence (n = 102, aa 1–419 from NCAP) synthesised by JPT Peptide Technologies (Berlin, Germany) and used at a final concentration of 2 μg/mL.

T-cell responses were characterised by measurement of the frequency of PBMC expressing IL-2 (PerCP5.5, 1:10; # 560708; MQ1-17H12, BD), IL-17a (Alexa700, 1:20; # 560613; N49-653, BD), IFN-γ (V450, 1:33.3; # 560371; B27, BD), TNF-α (BV605, 1:30.3; # 502936; Mab11, BioLegend), IL-13 (BV711, 1:20; # 564288; JES10-5A2, BD), CD137 (APC, 1:20; # 550890; 4B4, BD) and CD154 (FITC, 1:20; # 555699; TRAP1, BD) upon stimulation with the two peptide pools (RBD and NCAP). CD3 (APC-Cy7, 1:200; #557757; SP34-2, BD), CD4 (BV510, 1:33.3; # 563094; L200, BD) and CD8 (PE-Vio770, 1:50; # 130-113-159; BW135/80, Miltenyi Biotec) antibodies were used as lineage markers. Validation information for all antibodies was obtained from the corresponding manufacturer datasheets and reference webpages. Cross-reactivity was confirmed using the NHP reagent reactivity database or based on the “reactivity” information provided in manufacturer datasheets. One million PBMC were cultured in complete medium (RPMI1640 Glutamax+, Gibco; supplemented with 10% FBS), supplemented with co-stimulatory antibodies (FastImmune CD28/CD49d, Becton Dickinson). Then cells were stimulated with RBD sequence overlapping peptide pool at a final concentration of 2 μg/mL. Brefeldin A was added to each well at a final concentration of 10 μg/mL, and the plate was incubated at 37 °C, 5% CO2 during 18 h. Next, cells were washed, stained with a viability dye (LIVE/DEAD fixable Blue dead cell stain kit, ThermoFisher), and then fixed and permeabilized with the BD Cytofix/Cytoperm reagent. Permeabilized cell samples were stored at −80 °C before the staining procedure. Antibody staining was performed in a single step following permeabilization. After 30 min of incubation at 4 °C, in the dark, cells were washed in BD Perm/Wash buffer and then acquired on the ZE5 flow cytometer (Biorad). Analysis was performed on FlowJo v.10 software.

### Evaluation of immunoreaction of CD40.Pan.CoV manufacturing formulation

Dermal scoring was performed at days 0, 1, 3, and 7 after immunisation. Local skin reaction was scored using observations and graded scoring for erythema, oedema, bleeding, scabbing, fissuring and/or ulceration. Biochemistry parameters were analysed in lithium heparin plasma using standard kits (Siemens) and a canine kit (Randox) on an ADVIA1800 analyser (Siemens). Cytokines were quantified in EDTA-treated plasma using NHP Milliplex (PRCYTA-A40K-PX38, Millipore) and a Bioplex 200 analyser (Bio-Rad) according to manufacturer's instructions.

### Experimental infection of macaques with SARS-CoV-2

Four weeks after the last immunisation, animals were exposed to a total dose of 10^5^ TCID_50_ of SARS-CoV-2 B.1.617.2 Delta virus (NIH/BEI reference: NR-55612; Isolate hCoV-19/USA/PHC658/2021) or 10^5^ TCID_50_ of SARS-CoV-2 B.1.1.529 Omicron (NIH/BEI reference: NR-56462; Isolate hCoV-19/USA/MD-HP20874/2021) via the combination of intranasal and intra-tracheal routes (0.25 mL in each nostril and 4.5 mL in the trachea, i.e. a total of 5 mL; day 0), using atropine (0.04 mg/kg) for pre-medication and ketamine (5 mg/kg) with medetomidine (0.05 mg/kg) for anaesthesia. Nasopharyngeal and tracheal swabs, were collected at 1, 2, 3, 4, 5, 7, 10, 14, and 23 days post-exposure (d.p.exp.) while blood was taken at 2, 4, 7, 10, 14, and 23 d.p.exp. Bronchoalveolar lavages (BAL) were performed using 50 mL sterile saline at 3 d.p.exp in order to be close to the peak of viral replication and to be able to observe a difference between the vaccinated and control groups.

### Virus quantification in cynomolgus macaque samples

Upper respiratory (nasopharyngeal and tracheal) specimens were collected with swabs (Viral Transport Medium, VTM, CDC, DSR-052-01). Tracheal swabs were performed by insertion of the swab above the tip of the epiglottis into the upper trachea at approximately 1.5 cm of the epiglottis. All specimens were stored between 2 °C and 8 °C until analysis by RT-qPCR using Superscript III platinum one step qRT-PCR kit (Thermo Fisher), RdRp-IP4 specific primers and probe and a nine-point calibration curve consisted of a serial dilutions of a SARS-CoV-2 standard calibrated synthetic RNA (Exact Diagnostic) containing an RdRp gene fragment including the RdRp-IP4 RT-PCR target sequence (Table S1). The lower limit of detection was estimated to be 2.67 log_10_ copies of SARS-CoV-2 gRNA per mL and the lower limit of quantification was estimated to be 3.67 log_10_ copies per mL. SARS-CoV-2 E gene subgenomic RNA (sgRNA) was assessed by RT-qPCR using primers and probes previously described^24^ (Table S1). The lower limit of detection was estimated to be 2.87 log_10_ copies of SARS-CoV-2 sgRNA per mL and the lower limit of quantification was estimated to be 3.87 log_10_ copies per ml. Negative control consisted in VTM alone and positive control of VTM spiked with a known concentration of SARS-CoV-2. Samples, standards and controls were assessed in duplicate.

### Virus strains used in neutralisation assay

The Wuhan, Delta and XBB.1.5 strains have been described.^25^^,^^26^ The XFG.1.1 strain (hCoV-19/France/GES-RELAB-IPP04557/2025) was supplied by the National Reference Centre for Respiratory Viruses hosted by Institut Pasteur.

Viral strains were amplified through one or two passages on Vero E6 or IGROV-1 cells. Supernatants were harvested 2 or 3 days after viral exposure. The titration of viral stocks was performed on S-Fuse cells.^27^ Viral supernatants were sequenced directly from nasopharyngeal swabs and after isolation and amplification on Vero E6 or IGROV-1 cells.

The sequencing was performed as described previously.^26^

### S-Fuse neutralisation assay

U2OS-ACE2 GFP1-10 or GFP 11 cells, also termed S-Fuse cells, become GFP^+^ when they are productively infected by SARS-CoV-2.^27^ Cells were mixed (ratio 1:1) and plated at 8 × 10^3^ per well in a μClear 96-well plate (Greiner Bio-One). The indicated SARS-CoV-2 strains were incubated with serially diluted sera for 15 min at room temperature and added to S-Fuse cells. Sera were heat-inactivated for 30 min at 56 °C before use. 18 h later, cells were fixed with 2% PFA (Electron microscopy cat# 15714-S), washed and stained with Hoechst (dilution of 1:10,000, Invitrogen, Invitrogen cat# H3570). Images were acquired using an Opera Phenix high-content confocal microscope (PerkinElmer). The GFP area and the number of nuclei were quantified using the Harmony software (PerkinElmer). The percentage of neutralisation was calculated using the number of syncytia as value with the following formula: 100 × (1 – (value with serum – value in ‘non-infected’)/(value in ‘no serum’ – value in ‘non-infected’)).^28^ Neutralising activity of each serum was expressed as the half maximal effective dilution (ED_50_). ED_50_ values were calculated with a reconstructed curve using the percentage of neutralisation at each concentration.

### Principal component analysis

Principal component analysis (PCA) was performed using R software (version 4.4.1) with the ggplot2, dplyr, RColorBrewer, and ggrepel packages. Data were standardised (Z-score transformation) before PCA using the “prcomp” function with the parameters “center = TRUE” and “scale = TRUE”. The first two principal components were used for graphical representation. A continuous colour gradient was also applied to individual data points based on the expression level of the immunological variables of interest (anti-RBD IgG in serum and in BAL fluid, and IL-2^+^ CD4^+^ T cell response) or external variable (nasopharyngeal viral load AUC). Experimental groups were highlighted by polygons enclosing the individuals within each group.

### Modelling of the durability of vaccine-induced IgG binding response

#### Definition of the model

We studied the longevity of the RBD-specific IgG binding responses after the peak, observed at 14 days post vaccination, elicited by CD40 targeting vaccines in convalescent animals. We compared this longevity with the one induced by mRNA vaccines (monovalent or bivalent) in naive or convalescent animals. We used a pooled piecewise-linear model with zero or one breakpoint to capture the monophasic or biphasic forms of the dynamics, respectively, observed in the distinct groups. The consideration of these simple models was driven by the sparsity of our data and spline regression were performed to validate the linearity assumption between our outcome of interest and time, before and after the breakpoint (results not shown). Accordingly, the log_10_-transformed IgG binding responses were described as follows:

$$\log_{10}\left(Y_{ij}\right)=\left\{ \begin{array}{l} \beta_{0_{i}}\;+\;{\beta_{1}}_{i}\;\times \;t_{ij}\;+\;\varepsilon_{ij},if\;i\in \text{Monophasic}\\ {\beta_{0}}_{i}\;+\;\beta_{1_{i}}\;\times \;\min\left(t_{ij},\tau \right)\;+\;\beta_{2_{i}}\;\times \;\max\left(t_{ij}\;-\;\tau ,0\right)\;+\;\varepsilon_{ij},if\;i\in Biphasic \end{array}\right.$$

where *Y*_*ij*_ represents RBD-specific IgG binding response observed for animal *i* at time *t*_*ij*_ post-peak, with the parameters *β*_0_, *β*_1_ and *β*_2_ being the value at the peak, the first and the second slope, respectively, and *τ* the breakpoint between the two slopes. The variable *ε*_*ij*_ is the residual error assumed to be normally distributed of mean 0 and variance *σ*^2^. Macaques included in the durability analysis belonging to eight different groups of immunisation and showing distinct post-peak antibody dynamics, model parameters were adjusted for groups and random effects using linear mixed-models. Each parameter *θ* ∈ {*β*_0_,*β*_1_,*β*_2_} of the model is defined by the following linear-mixed model:

$$\begin{array}{ccc} & \theta_{i}=\theta_{pop}\;+\sum_{g\;=\;1}^{G\;-\;1}\phi_{\theta }^{g}\;I_{i\in g}\;+\;u_{i}^{\theta }\; & u_{i}^{\theta }∼N\left(0,\omega_{\theta }^{2}\right) \end{array}$$

where *θ*_*pop*_ represents the mean value of *θ* within the population, $\phi_{\theta }^{g}$ the regression coefficient associated with the categorical covariate of group *g* = 1, …,*G*-1, with *G* being the number of groups involved in the modelling, and considering the group CD40.RBDv vaccinated Wuhan SARS-CoV-2 convalescent as the reference. Finally, $u_{i}^{\theta }$ is the random effect on *θ* assumed normally distributed with mean 0 and standard deviation *ω*_*θ*_. To avoid overfitting due to the sparsity of our data, the breakpoint *τ* was assumed as not adjusted for random effects or covariates (models with adjustments on *τ* have been tested and rejected based on the corrected Bayesian information criterion, BICc).

#### Model building strategy

To identify which adjustments for group effects should be considered on model parameters, we applied the classic Stepwise Covariate Modelling (SCM) method implemented in Monolix 2024R1 (Monolix, Lixoft) using the BICc as selection criterion. The algorithm was initiated with the model free of covariates (i.e. $\forall g\;\forall \theta \;\phi_{\theta }^{g}=0$), all groups of vaccination were coded as binary covariates (0/1) to allow the maximum of flexibility to the algorithm and the adjustment of the three parameters *β*_0_, *β*_1_ and *β*_2_ for all covariates was allowed (except for the monophasic groups on *β*_2_). This algorithm was only applied to build the optimal model on antibody responses against RBD Wuhan VOCs. The selected model was then only estimated on antibody responses against RBD Alpha, Beta and Delta VOCs.

#### Model estimation and validation

The pooled model was implemented in Monolix and the estimation of population parameters was performed using the stochastic approximation expectation-maximisation algorithm. The model was evaluated independently for antibody responses against RBD Wuhan, Alpha, Beta and Delta VOCs. For each VOC, once the model was estimated, we validated it by verifying i) the significance of all selected covariates $\beta_{\theta }^{g}$ using Wald tests, ii) the normality of random effects and residuals using Shapiro-Wilk tests, and iii) the shrinkage of individual parameter distribution (Figure S2).

#### Derivation of the time to reach specific IgG cutoff

Once the model estimated, we used it to derive the relationship between the level of RBD specific IgG and the average time required to reach it in each group of animals. Due to the simplicity of the model, a literal expression of this relationship can be defined. Let note *Z* the target level of IgG (in log10 scale) and *T*_*Z*_ the time at which this level is reached, such that *log*_10_*Y*(*T*_*Z*_) = *Z*. The following algorithm can be applied to calculate *T*_*Z*_:

• If *Z* belongs to the first decreasing phase (i.e. monophasic or *Z* ∈ [*β*_0_ + *β*_1_*τ*;*β*_0_]) then *T*_*Z*_ = (*Z*-*β*_0_)/*β*_1_
• Else if Z belongs to the second decreasing phase (i.e. biphasic and *Z* ∈ (− *∞*;*β*_0_ + *β*_1_*τ*] then *T*_*Z*_ = [*Z*-*β*_0_-(*β*_1_-*β*_2_)*τ*]/*β*_2_
• Otherwise (i.e. *Z* ∈ [*β*_0_; + *∞*) or *β*_2_ > 0) *T*_*Z*_ can not be calculated.

To account for uncertainty in parameter estimation, we used a Monte-Carlo sampling approach, where *K*_*pop*_ = 1000 sets of population parameters were sampled from their empirical posterior distributions. Then to include between-subjects variability, we added a second layer by sampling *K*_*ind*_ = 55 sets of individual parameters (corresponding to the number of animals included in the model) from the population parameter distribution. For each set of simulated parameters, we were then able to calculate the predicted trajectories and the relationship between *Z* and *T*_*Z*_. From these simulations, we obtained for each group the 95% prediction intervals of the predicted trajectories and the distribution of *T*_*z*_ for all tested values of *Z* and extract i) the percentage of simulated parameters for which *T*_*z*_ was correctly calculated, ii) the median (i.e. 50th percentile), the 50% prediction intervals (i.e. 25th and 75th percentiles) and the 95% prediction intervals (i.e. 2.5th and 97.5th percentiles).

### Statistical analysis

Sample size was determined as the minimal number allowing non-parametric statistical analysis while complying with the 3Rs rule on reducing, replacing and refining the use of animals for scientific purpose. Convalescent and naïve animals we randomised separately using ALEA function in excel. For security reason, animal ID and experimental group are indicated on the housing cage, thus animals care, clinical examination and sampling was not blinded because constrains associated to BSL3 containment. All experimenters are blinded to the groups during sampling, immunisations, challenge, experiment. Quantification of SARS-CoV-2 antibodies, neutralisation assays, ICS, dermal scoring, biochemistry parameters, luminex assays and viral loads were determined blinded. Data were collected using classical Excel files (Microsoft Excel 2016). Differences between unmatched groups were compared using the two-tailed non-parametric Mann-Whitney test or the Kruskal-Wallis test following Dunn's multiple comparisons (Graphpad Prism 9.4.1), and differences between paired measurements (repeated measures form the same animals, e.g. before and after vaccination) were compared using the Wilcoxon matched-pairs signed rank test (Graphpad Prism 9.4.1). Correlation between viral and immune parameters was determined using nonparametric Spearman correlation (Graphpad Prism 9.4.1). Mathematical modelling of the durability of binding responses was performed using Monolix2024R1 and results were analysed using R version 4.2.2.

### Role of funders

The funding sources were not involved in the study design, data acquisition, data analysis, data interpretation, or writing of the manuscript.

## Results

### Design of dendritic cell targeting SARS-CoV-2 vaccines

**Fig. 1:**
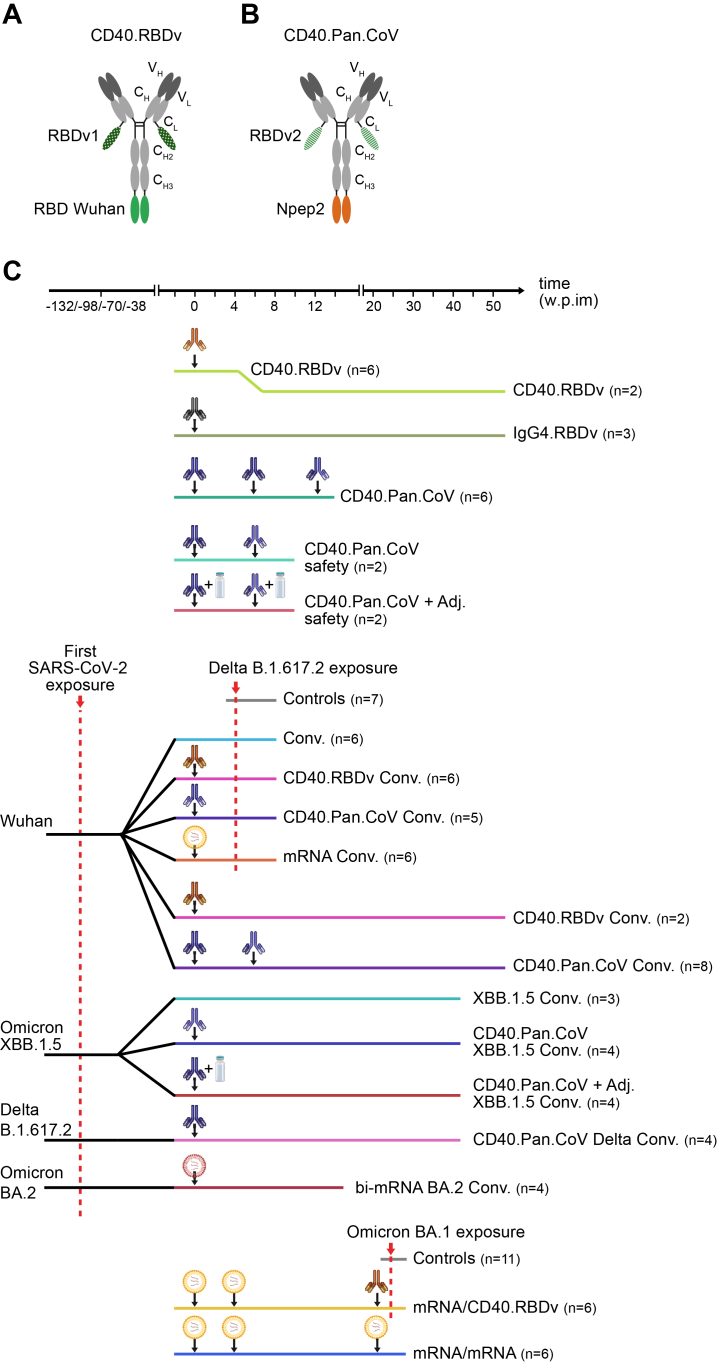
**Design of CD40.RBDv and CD40.Pan.CoV vaccines and schematic overview of vaccination strategies in naive and SARS-CoV-2 convalescent macaques.** (**A and B**) Schematic representation of CD40.targeting vaccines. (**A**) The CD40.RBDv vaccine consists of a humanised anti-CD40 IgG4 clone 12E12 fused via the Ct-HC to the RBD from the original SARS-CoV-2 Wuhan strain and via the Ct-LC to the RBDv1 with K417N, E484K N501Y mutations. (**B**) The CD40.Pan.CoV vaccine consists on humanised anti-CD40 IgG4 clone 12E12 fused via the Ct-HC to Npep2 and via the Ct-LC to the RBDv2 harbouring K417N, L452R, T478K, E484Q, N501Y mutations. (**C**) Schematic overview of vaccination strategies in naive, SARS-CoV-2 convalescent and mRNA-vaccinated macaques. In naive animals, cynomolgus macaques were injected subcutaneously (SC) on week 0 with CD40.RBDv (n = 6, light green). Safety parameters were analysed in naive animals, that were injected with CD40.Pan.CoV alone (n = 2, turquoise) or with CD40.Pan.CoV adjuvanted with Poly–IC–LC (n = 2, Indian red) on weeks 0 and 6. For durability analysis, some animals were injected SC on week 0 with CD40.RBDv (n = 2, light green) or IgG4.RBDv (n = 3, dark green). Cynomolgus macaques were injected subcutaneously (SC) on weeks 0, 6 and 12 with CD40.Pan.CoV (n = 6, dark cyan). In Wuhan convalescent animals (range = 98–70 weeks of SARS-CoV-2 convalescence), CD40.RBDv (n = 6, pink), CD40.Pan.CoV (n = 5, purple) or BNT162b2 mRNA vaccine (n = 6, orange) was administered on week 0. Non-vaccinated SARS-CoV-2 convalescent (n = 6, light blue) and non-vaccinated SARS-CoV-2-naive (n = 7, grey) macaques were included as controls. For durability analysis in Wuhan convalescent macaques, CD40.RBDv (n = 2, pink) was administrated on week 0 and CD40.Pan.CoV (n = 8, purple) on week 0 and 6. In Omicron XBB.1.5 convalescent animals (61−38 weeks of convalescence), either CD40.Pan.CoV alone (n = 4, dark blue) or CD40.Pan.CoV adjuvanted with Poly–IC–LC (n = 4, firebrick) was injected SC on week 0. Non-vaccinated Omicron XBB.1.5 convalescent macaques (n = 3, light sea green) were included as controls. In Delta convalescent animals (132−127 weeks of convalescence), CD40.Pan.CoV alone (n = 4, light pink) was injected SC on week 0. In Omicron BA.2 convalescent animals (47 weeks of convalescence) BNT162b2 bivalent mRNA vaccine (n = 4, dark red) was injected on week 0. Finally, immunogenicity of CD40.RBDv vaccine was analysed as a booster in mRNA immunised animals. Macaques were injected intramuscularly (IM) on weeks 0 and 4 with BNT162b2 mRNA vaccine (Pfizer-BioNTech). On week 21, animals were injected with CD40.RBDv (n = 6, yellow) or with BNT162b2 mRNA vaccine (n = 6, blue). Non-vaccinated macaques (n = 11, grey) were included as controls. Efficacy of CD40.RBDv and CD40.Pan.CoV was analysed after exposure to SARS-CoV-2 (Delta or Omicron BA.1) 4 weeks after the last vaccine injection.

Two subunit SARS-CoV-2 vaccine candidates were rationally designed and engineered using an anti-CD40 platform. Both constructs share a common backbone consisting of the humanised IgG4 mAb 12E12, which specifically targets human CD40, and is fused to SARS-CoV-2 antigens to facilitate targeted delivery to antigen-presenting cells. The first candidate, CD40.RBDv, is a bivalent vaccine in which the C-terminus of the heavy chain (Ct-HC) of 12E12 mAb is fused to Spike (S) Receptor-Binding Domain (RBD) of the original SARS-CoV-2 Wuhan strain, while the C-terminus of the light chain (Ct-LC) is fused to a variant RBD (RBDv1) containing the K417N, E484K and N501Y mutations found in VOCs (Fig. 1A). To extend the breadth of COVID-19 vaccines based on the use of the SARS-CoV-2 S antigen, the second candidate, CD40.Pan.CoV, included the 12E12 mAb fused via the Ct-HC to a non-S Nucleocapsid (NCAP) sequence (Npep2) from SARS-CoV-2, while the Ct-LC was fused to the RBD sequence containing K417N, L452R, T478K, E484Q, N501Y mutations (RBDv2) (Fig. 1B).

We engineered vectors expressing vaccine sequences to generate the CD40.RBDv and CD40.Pan.CoV vaccines. Both vaccines and IgG4 constructs (non-targeting antibodies fused to the same vaccine antigens) were produced in CHO cells and controlled for their quality (Figure S3). As evaluated by a solid-phase direct-binding assay and as previously reported, there was no significant difference in CD40 binding affinity (EC_50_ 30 pM) between the 12E12 anti-CD40 mAb and 12E12 anti-CD40 fused to different antigens. We have previously shown that the 12E12 anti-CD40 mAb fused to different viral antigens is capable of i) enhancing CD40-mediated internalisation and antigen-presentation by human mononuclear cells and *ex vivo*-generated monocyte-derived dendritic cells,^13^^,^^14^ and ii) binding CD40 receptor and activating non-human primate monocytes, DCs, and B cells.^17^^,^^29^

### Non-adjuvanted sub-unit CD40-based vaccines elicit systemic and mucosal anti-SARS-CoV-2 responses in SARS-CoV-2 naive macaques

**Fig. 2:**
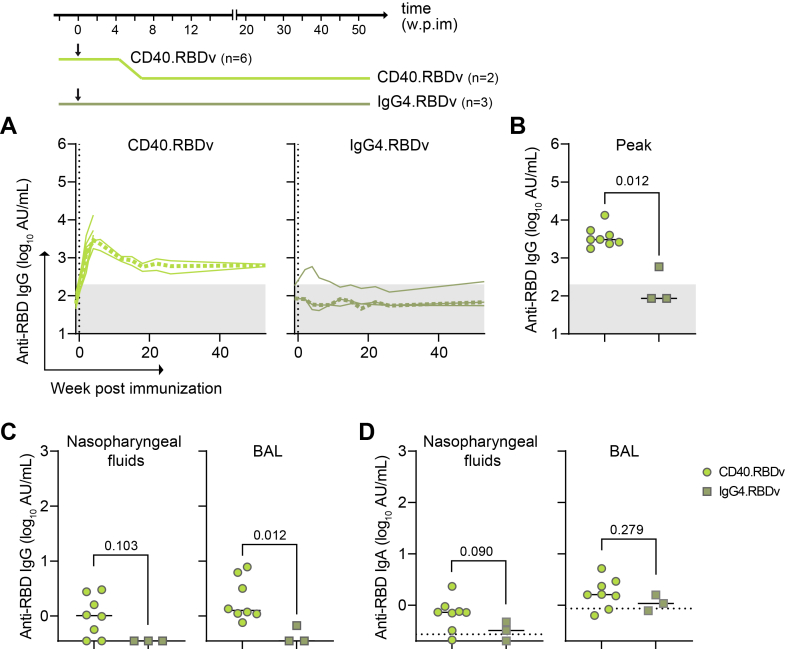
**SARS-CoV-2 specific Abs induced by CD40 targeting vaccine in naive NHP.** Binding IgG against Wuhan RBD were measured in serum (**A and B**) and fluid (**C and D**) samples using electrochemiluminescence assay (ECL). (**A**) Individual serum measures and median (thick dotted lines) are indicated for CD40.RBDv vaccinated animals (n = 8, light green) and IgG4.RBDv vaccinated animal (n = 3, dark green). Dotted vertical line represents vaccine administration and grey band indicates positivity cutoff: 200 AU/mL. (**B**) Peak responses were analysed in serum. (**C and D**) At week 3 post immunisation IgG and IgA against Wuhan RBD were measured in nasopharyngeal fluids (left) and bronchoalveolar lavages (BAL) (right). CD40.RBDv (light green circles) and IgG4.RBDv (dark green squares) groups were compared using the two-tailed non-parametric Mann-Whitney test. Dotted horizontal line indicates the median of non-immunised control animals. Line indicates the median.

We assessed the immunogenicity of non-adjuvanted CD40.RBDv (n = 8) and CD40.Pan.CoV (n = 6) vaccines in SARS-CoV-2 naive cynomolgus macaques (Fig. 1C). In a first set of experiments, a single SC injection of 200 μg of CD40.RBDv induced anti-RBD antibodies in serum peaking at week 4 (median titres [IQR]: 3050 [2426–4966] AU/mL). Response was then followed in two of these animals, which still had detectable specific antibodies at week 53 (633 [590–676] AU/mL) (Fig. 2A). The anti-RBD titres in those macaques were comparable to those we previously observed in Wuhan convalescent humans and macaques. The advantage of CD40 targeting was underscored by the low immunogenicity of SC injection of IgG4.RBDv (non-targeted vaccine) at week 4 (86 [85–585] AU/mL) and below the positivity cutoff at week 53 in control animals (n = 3) (Fig. 2A–B). Targeting antigens to CD40 also favours mucosal responses. At week 3, CD40.RBDv induced RBD specific IgG against Wuhan, Alpha (B1.1.7), Beta (B.1.351) and Delta (B.1.617.2) VOCs in upper respiratory tract (nasopharyngeal fluids; IgG anti-RBD Wuhan: 1.01 [0.40–2.47] AU/mL) and lower respiratory tract (broncho-alveolar lavages or BAL: 1.26 [1.10–5.44] AU/mL) while these values remained under the limit of detection in IgG4.RBDv recipients (median difference = 0.83, 95% CI [0.41–7.46], p = 0.012 in BAL) (Fig. 2C and Figure S4). Cross-reactive IgA against RBD were also detected in these fluids at week 3 post vaccine injection (Fig. 2D and Figure S4c).

**Fig. 3:**
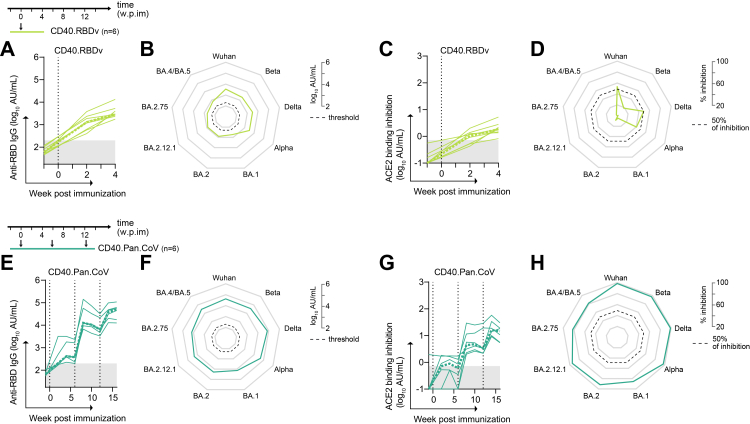
**Breadth increase of Ab response in naive NHP by the next generation of CD40 targeting vaccines.** IgG binding to Wuhan RBD (**A and E**) and inhibition of binding to human ACE2 (**C and G**) were analysed in serum samples of naive animals. Individual values and median (thick dotted lines) were indicated for CD40.RBDv vaccinated animals (n = 8, light green, **A and C**) and CD40.Pan.CoV vaccinated animal (n = 6, dark cyan, **E and G**). Dotted vertical line represents vaccine administrations and grey band indicates positivity cutoff. Radar plots represent the Ab titres in serum (**B and F**) or the percentage of inhibition of ACE2 binding by animal serum (**D and H**) against different VOC RBD at 4 weeks post last vaccine injection (i.e. following a single injection of CD40.RBDv or 3 injections of CD40.Pan.CoV). Coloured line represents the median group and dotted line indicates positivity cutoff (**B and F**) or 50% of inhibition (**D and H**).

Analysis of the breadth of CD40.RBDv-induced RBD-specific IgG binding responses in serum showed comparable titres against RBD Wuhan, Alpha, Beta and Delta variants (Fig. 3A, Figure S5a and S5d) and a 4.4–6.5 fold-decrease against Omicron variants (Fig. 3B and Figure S5d). The CD40.RBDv vaccine induced specific antibodies that can inhibit the binding of RBD proteins to human ACE2 (Fig. 3C, Figure S5b and S5e), however, with limited reactivity against the more recent VOCs (less than 10% inhibition against Omicron variants) (Fig. 3D and Figure S5e).

In a second set of experiments, non-adjuvanted CD40.Pan.CoV vaccine was administered in SARS-CoV-2 naive animals (n = 6; 200 μg at weeks 0, 6 and 12) (Fig. 1C). Four weeks after the last injection (week 16) titres of anti-RBD Wuhan IgG rose to 41,909 AU/mL [IQR 14,760–65,799] (Fig. 3E) and remained stable against RBD Alpha, Beta and Delta VOCs while only a slight decrease (1.4–3.1 fold) was noted against Omicron variants (Fig. 3F, Figure S5a and S5d), highlighting the benefits of multiple administrations in a naive settings for a stronger Ab response in the absence of an adjuvant. IgG inhibiting ACE2 binding elicited by CD40.Pan.CoV vaccine remained high (median: 97%; [IQR 85–99%]) regardless of RBD sequence (82% against RBD BA.4/BA.5) (Fig. 3G–H and Figure S5b and S5e). The CD40.Pan.CoV induced NCAP-specific IgG in naive macaques, confirming the immunogenicity of the vaccine Npep2 sequence (Figure S5c). CD40.Pan.CoV vaccine induced also mucosal responses with RBD specific IgG and IgA against Wuhan and VOCs in nasopharyngeal and BAL fluids measured 3 weeks after the last vaccine administration (Figure S5f–i).

The reactogenicity of the CD40.Pan.CoV vaccine was assessed in its manufacturing formulation. In SARS-CoV-2 naive animals, the non-adjuvanted formulation (n = 2) was compared with Poly-ICLC (Hiltonol®)-adjuvanted CD40.Pan.CoV (n = 2). Mild erythema was observed at the injection site with both formulations, and only at D1 post injection (Figure S6a). Increases in inflammatory proteins and cytokines occurred solely following administration of the adjuvanted CD40.Pan.CoV formulation, and these effects remained transient (Figure S6b–c). While anti-RBD antibody titres were higher in the adjuvanted group (Figure S6d), both vaccine formulations demonstrated immunogenicity, with two doses of non-adjuvanted CD40.Pan.CoV sufficient to elicit IgG capable of inhibiting ACE2 against multiple strains (Figure S6e).

### Non-adjuvanted CD40 targeting vaccines recall specific and long-lasting immune responses in SARS-CoV-2 convalescent macaques

**Fig. 4:**
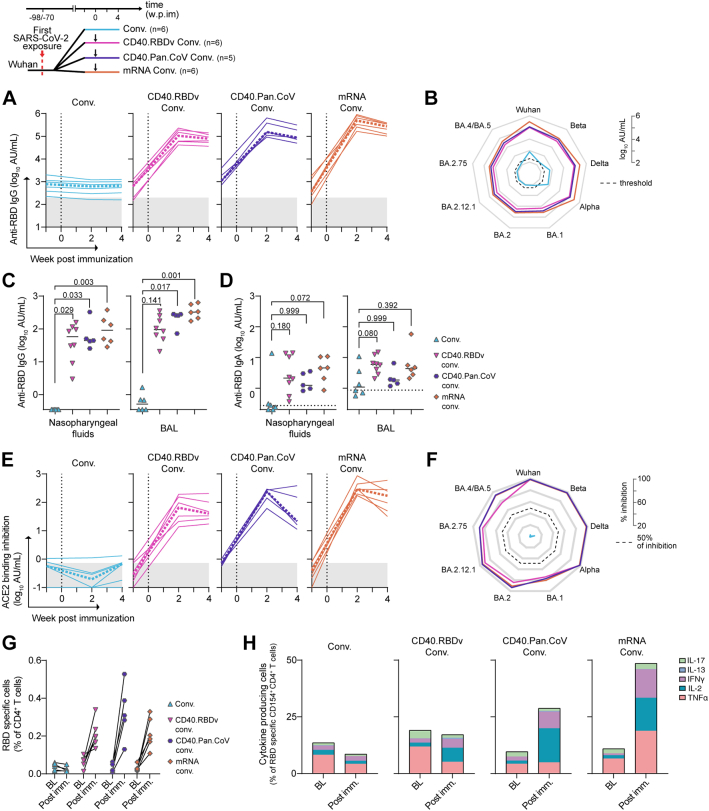
**CD40 targeting vaccines recall specific and sustainable immune responses in convalescent macaques.** IgG binding to Wuhan RBD (**A and B**) and inhibition of binding to human ACE2 (**E and F**) were analysed in serum samples of SARS-CoV-2 convalescent animals. Individual values and median (thick dotted lines) were indicated for non-vaccinated (n = 6, light blue); CD40.RBDv (n = 6, pink), CD40.Pan.CoV (n = 5, purple) and mRNA vaccinated convalescent animals (n = 6, orange). Dotted vertical line represents vaccine administrations and grey band indicates positivity cutoff. Radar plots represent Ab titres in serum (**B**) or the percentage of inhibition of ACE2 binding by animal serum (**F**) against different VOC RBD at 4 weeks post vaccine injection. Coloured line represents the median group and dotted line indicates positivity cutoff (**B**) or 50% of inhibition (**F**). Binding IgG (**C**) and IgA (**D**) against Wuhan RBD in nasopharyngeal fluids (left) and bronchoalveolar lavages (right) were compared between groups using Kruskal-Wallis test following Dunn's multiple comparisons. Dotted horizontal line indicates the median of non-immunised control animals. (**G**) Frequency of RBD-specific CD4^+^ T cells (CD154^+^/CD137^+^) in the total CD4^+^ T cell population for each non-vaccinated (n = 6, light blue), CD40.RBDv (n = 6, pink), CD40.Pan.CoV (n = 5, purple) and mRNA vaccinated convalescent animals (n = 6, orange) 2 weeks post vaccine injection. PBMC were stimulated overnight with SARS-CoV-2 RBD overlapping peptide pool. Time points in each experimental group were compared using the Wilcoxon signed rank test. (**H**) Frequency of cytokine producing cells in the RBD-specific CD4^+^ T cells (CD154^+^) for non-vaccinated, CD40.RBDv, CD40.Pan.CoV, and mRNA vaccinated convalescent animals. Each bar indicates the median of the 6 or 5 vaccinated convalescent macaques. Distribution of cytokines is indicated within each bar. BL: Baseline before immunisation; “Post imm.”: Two weeks post immunisation.

Macaques convalescent from SARS-CoV-2 Wuhan infection (70–98 weeks post-infection) were randomly assigned to receive a single injection of non-adjuvanted CD40.RBDv (n = 6), CD40.Pan.CoV (n = 5) or BNT162b2 mRNA vaccine (n = 6, Fig. 1C). Four weeks post-injection, titres of anti-RBD Wuhan in serum rose to 10,238 [IQR 67,339–159,880] and 112,990 [IQR 83,136–330,903] AU/mL in CD40.RBDv and CD40.Pan.CoV vaccinated animals, respectively (Fig. 4A–B and Figure S7a and S7c), while non-vaccinated convalescent macaques used as controls maintained low titres (n = 6; 786 [288–1274] AU/mL). Titres of anti-RBD IgG induced by the CD40 constructs were similar to those observed in mRNA-vaccinated animals (Fig. 4A and B and S7a & S7c). Recall and high titres of anti-NCAP specific IgG were elicited after one injection of CD40.Pan.CoV in all animals (Figure S7g). As compared to the Wuhan strain, IgG against the Omicron strains was also observed in both groups of CD40 vaccinated animals but were 5.3–6.7 and 3.4–4.5 fold lower than the Wuhan strain with CD40.RBDv and CD40.Pan.CoV vaccines, respectively (Fig. 4B and Figure S7c). A similar profile was observed with mRNA vaccine, in which IgG against Omicron variants were 7.2–9.5 fold lower than anti-RBD Wuhan IgG titres (Fig. 4B and Figure S7c). At week 3 post vaccination, all vaccines elicited a significant higher level of anti-RBD specific binding IgG in nasopharyngeal fluids (median difference = 79.48, 95% CI [8.64–114.64], p = 0.029 for CD40.RBDv; and median difference = 43.99, 95% CI [25.02–327.39], p = 0.033 for CD40.Pan.CoV) and BAL (median difference = 257.82, 95% CI [72.12–280.34], p = 0.017) (Fig. 4C) while non-vaccinated convalescent animals maintained low antibody levels. Specific IgA were also measured in nasopharyngeal fluids and BAL in vaccinated animals (Fig. 4D). Significant increases in binding mucosal responses (IgG and IgA) against RBD Alpha, Beta, and Delta VOCs, were observed in both vaccine groups (Figure S7e–f). The mucosal antibody responses measured after mRNA vaccination were similar to those induced with the CD40 vaccine constructs (Fig. 4C–D and Figrue S7e–f). Both vaccines elicited cross-neutralising activities up to 99% against RBD Wuhan, Alpha, Beta, and Delta VOCs which remained high with 84% (median; [IQR 77–89%]) and 94% (median; [IQR 84–95%]) inhibitory activity against Omicron VOCs in the CD40.RBDv and CD40.Pan.CoV groups, respectively, while no neutralising activities were detectable in non-vaccinated convalescent animals (Fig. 4E–F, Figure S7b and S7d). Additional mutations included in the CD40.Pan.CoV construct compared to CD40.RBDv increased the breadth of the antibody responses, particularly against RBD sequences containing the L452R mutation (Figure S8). In addition, the neutralising activity induced by CD40.Pan.CoV vaccine were identical to that measured after mRNA vaccination of convalescent individuals (Fig. 4E–F and Figure S7d).

Before vaccination, frequencies of RBD-specific CD4^+^ T cells were low and similar in all groups of convalescent macaques (median 0.04 [IQR 0.019–0.060]%). Two weeks after vaccination, median frequencies of these cells increased to 0.20 [IQR 0.16–0.26]% (median difference = 0.15, 95% CI [0.03–0.27], p = 0.031 as compared to baseline) in the CD40.RBDv group and to 0.3 [IQR 0.21–0.46]% in the CD40.Pan.CoV group, which showed a trend toward increased responses (median difference = 0.30, 95% CI [0.12–0.48], p = 0.062), while no changes were noted in the non-vaccinated convalescents (Fig. 4G). Similar increase of RBD-specific CD4^+^ T cells was measured in mRNA group after vaccination (median 0.19 [IQR 0.15–0.30]%; median difference = 0.17, 95% CI [0.08–0.31], p = 0.031 as compared to baseline). The quality of these specific T-cell responses was also modified by the CD40 vaccines with an increased frequency of cells producing IL-2 and IFN-γ after vaccination (Fig. 4H and Figure S9a–b). RBD-specific CD8^+^ T cell responses were not detectable in vaccine or control groups (Figure S9a). We confirmed the T-cell immunogenicity of the Npep2 sequence in recipients of the CD40.Pan.CoV vaccine, which is also mediated by Th1 CD4^+^ T cells (Figure S9c).

**Fig. 5:**
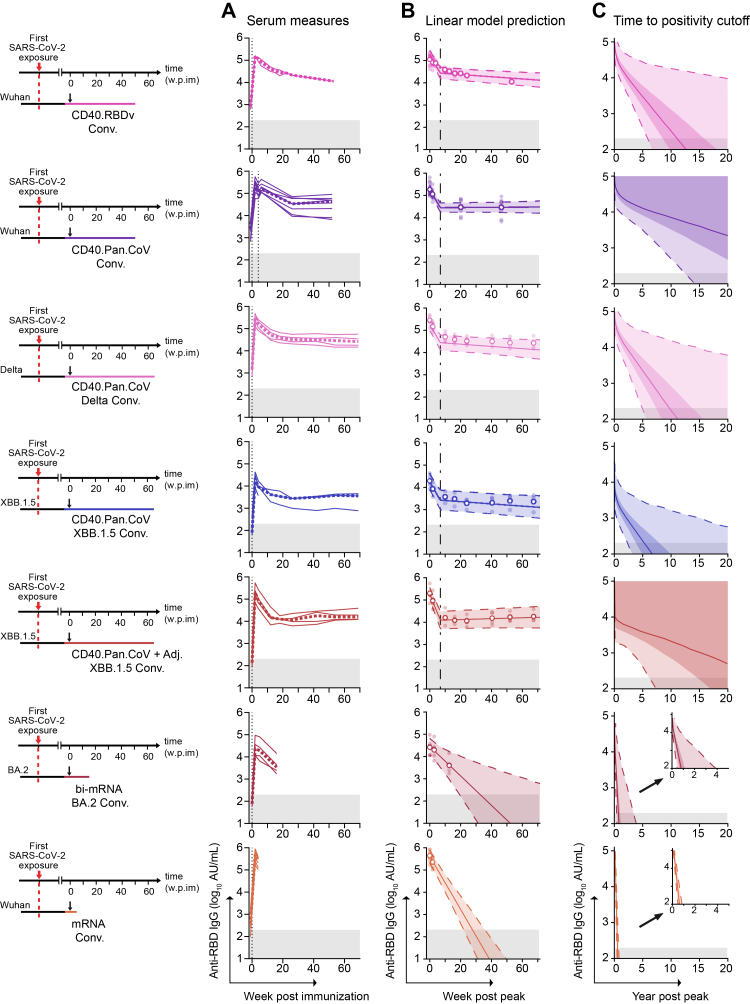
**Analysis of the durability of anti-SARS-CoV-2 antibodies binding to Wuhan RBD in SARS-CoV-2 convalescent vaccinated NHPs. (A)** IgG binding to Wuhan RBD were analysed in serum samples of SARS-CoV-2 convalescent animals. Individual values (thin lines) and median (thick dotted lines) were indicated for CD40.RBDv vaccinated Wuhan SARS-CoV-2 convalescent (n = 8, pink), CD40.Pan.CoV vaccinated Wuhan SARS-CoV-2 convalescent (n = 13, purple), CD40.Pan.CoV vaccinated Delta SARS-CoV-2 convalescent (n = 4, light pink), CD40.Pan.CoV vaccinated XBB.1.5 SARS-CoV-2 convalescent (n = 4, dark blue), adjuvanted CD40.Pan.CoV vaccinated XBB.1.5 SARS-CoV-2 convalescent animals (n = 4, firebrick), bivalent mRNA vaccinated BA.2 SARS-CoV-2 convalescent (n = 4, dark red), and mRNA vaccinated Wuhan SARS-CoV-2 convalescent (n = 6, orange). Dotted vertical line represents vaccine administrations. **(A**–**C)** Grey band indicates positivity cutoff. **(B)** Post-peak IgG binding to Wuhan RBD predicted by the pooled monophasic-biphasic linear mixed model. Median (thick solid line) and its 95% confidence interval (coloured shaded area) predicted by the model are compared to observations with median and individual observed dynamics represented by large white dots and small circles, respectively. The black vertical dash-dotted line indicates the breakpoint time *τ* estimated in the model for groups described by the biphasic model. **(C)** Relationship between IgG binding to Wuhan RBD levels and time post peak, in years, required to reach them predicted by the model. Median prediction is indicated by the solid line, and 95% and 50% prediction intervals are displayed by light and dark shaded areas, respectively. For mRNA groups, a focus of prediction over 5 years was embedded.

For analysis of durability, Wuhan convalescent macaques were injected SC at week 0 with CD40.RBDv (n = 2) or at weeks 0 and 6 with CD40.Pan.CoV (n = 8). Anti-RBD Wuhan and VOCs Alpha, Beta, and Delta binding and ACE2 binding inhibiting IgG titres described biphasic dynamics from the peak response. After a first decrease, anti-RBD IgG titres remained high and stable up to 53 weeks (up to 11,430 AU/mL and 43,166 AU/mL in CD40.RBDv and CD40.Pan.CoV groups, respectively) (Fig. 5A and Figure S10a–b). The same profile was observed in convalescent macaques previously infected with the Delta strain (127–132 weeks post-infection) and vaccinated with CD40.Pan.CoV (n = 4). Anti-RBD IgG titres remained high and stable 42 weeks after vaccination (31,555 [IQR 19,994–53,729] AU/mL) (Fig. 5A and Figure S11). Vaccination with CD40.Pan.CoV in Omicron XBB.1.5 convalescent NHPs (38–61 weeks post-infection) also elicited anti-RBD specific binding IgG but at a lower level compared to Delta convalescent animals (Fig. 5A and Figure S11). Ongoing clinical trials are testing the booster effect of non-adjuvanted or Poly-ICLC (Hiltonol®) adjuvanted CD40.RBDv (NCT06255626, EuCT 2023-504594-20-00) or CD40.Pan.CoV (EuCT 2024-516767-92-01). Therefore, in an additional experiment, we tested poly-ICLC-adjuvanted CD40.Pan.CoV as a boost in Omicron XBB.1.5 convalescent NHPs. We showed that this combination increases both anti-RBD binding and neutralising IgG responses at similar levels compared to CD40.Pan.CoV vaccinated Delta convalescents (Fig. 5A and Figure S11). In all groups, a statistical model represented the dynamics of the response by a first phase of rapid decrease, with an estimated duration of about 6/7 weeks, followed by a more stable plateauing phase (Figure S12a). The first phase average decay rates range from −0.12 to −0.17 log_10_ AU/mL per week, respectively in Wuhan convalescent CD40.RBDv and Omicron XBB.1.5 convalescent adjuvanted CD40.Pan.CoV (Table 1 and Table S2). In the second phase, the average decay rates were low, indicating stable IgG titres, in all CD40.Pan.CoV groups (Fig. 5B and Figure S12c). We estimated the maintenance of anti-RBD IgG binding titres above the limit of positivity for more than 5.28 years in all groups, ranging from 9.74 to 38.96 years in Wuhan and Delta convalescent CD40.Pan.CoV and in Omicron XBB.1.5 convalescent adjuvanted CD40.Pan.CoV animals (Fig. 5C, Table 1 and Table S2). In comparison, we analysed the dynamics of anti-RBD IgG titres following vaccination with bivalent mRNA (Wuhan/BA.4-5) in Omicron BA.2 convalescent NHPs and monovalent mRNA (Wuhan) in Wuhan convalescent NHPs (Fig. 5A). In contrast to the CD40 groups, the statistical model described the response dynamics in the mRNA groups using a monophasic phase (Fig. 5B and Figure S12b–c). Thus, the maintenance of anti-RBD IgG titres above the limit of positivity was estimated to last 0.56 to 0.71 years (Fig. 5C, Table 1 and Table S2).

**Table 1:** Estimation of the pooled monophasic-biphasic linear mixed model on anti-SARS-CoV-2 antibodies binding to Wuhan RDB for the convalescent groups vaccinated either by the CD40 targeting vaccines or mRNA vaccines, and prediction of the time required to reach the positivity cutoff of 200 AU/mL.

	Breakpoint (τ) (days post-peak) Mean [95% CI] P-value^c^	Peak value (β0) (log10 AU/mL) Mean [95% CI] P-value^c^	First slope (β1) (log10 AU/mL/Week) Mean [95% CI] P-value^c^	Second slope (β2) (log10 AU/mL/Week) Mean [95% CI] P-value^c^	Time to the positivity cutoff^a^ (years post-peak) Median [Q1; Q3] Cutoff reached (%)^d^
Groups described by the biphasic part of the model					
CD40.RBDv Conv. [reference]	47.97 [46.71–49.22] (na)	5.27 [5.18–5.35]	−0.120 [−0.130 to −0.109]	−0.005 [−0.007 to −0.003]	11.10 [8.54–15.86] (94.4%)
CD40.Pan.CoV Conv.		5.27 [5.18–5.35] (na)	−0.120 [−0.130 to −0.109] (na)	0.000 [−0.002 to 0.003] (∗∗∗)	38.96 [24.25–75.93] (44.4%)
CD40.Pan.CoV Delta Conv.		5.27 [5.18–5.35] (na)	−0.120 [−0.130 to −0.109] (na)	−0.005 [−0.007 to −0.003] (na)	9.74 [7.37–13.80] (94.8%)
CD40.Pan.CoV XBB.1.5 Conv.		4.25 [3.97–4.52] (∗∗∗)	−0.120 [−0.130 to −0.109] (na)	−0.005 [−0.007 to −0.003] (na)	5.28 [3.82–7.95] (94.4%)
CD40.Pan.CoV + Adj XBB.1.5 Conv.		5.27 [5.18–5.35] (na)	−0.172 [−0.203 to −0.141] (∗∗)	0.002 [−0.002 to 0.006] (∗∗∗)	24.96 [14.65–56.85] (27.0%)
Groups described by the monophasic part of the model					
Bi-mRNA BA.2 Conv.	b	4.45 [4.18–4.72] (∗∗∗)	−0.066 [−0.096 to −0.037] (∗∗∗)	b	*0.71*^b^*[0.56*–*1.00] (97.7%)*
mRNA Conv.	b	5.62 [5.39–5.84] (∗∗)	−0.120 [−0.130 to −0.109] (na)	b	*0.56*^b^*[0.51*–*0.62] (100%)*

a Threshold of 200 AU/mL. The median, Q1 and Q3 were extracted as the 50th, 25th, and 75th percentiles of the distribution of the time to positivity cutoff calculated on parameters simulated from the empirical posterior distribution. The reader can refer to the Supplementary Materials for more information.

b The available data do not allow estimation of a second slope because the follow-up duration is insufficient, strongly impacting the calculation of the time requested to reach the positivity cutoff. Italicized values indicate estimates derived from groups with limited follow-up data and should therefore be interpreted with caution due to their greater dependence on model assumptions.

c P-values of the Wald tests performed to verify the significance of group effects; na: not adjusted, ns: not significant, ∗ p < 0.05, ∗∗ p < 0.01, ∗∗∗ p < 0.001. The group CD40.RBDv Conv. being considered as the group of reference, no adjustment for group effects have been performed on it.

d The percentage of simulations for which the simulated parameters allowed to reach the positivity cutoff. CI, confidence interval; Q1, first quartile; Q3, third quartile.

Finally, we extended the characterisation of the neutralising activity of antibody responses to CD40-vaccines by testing the functional neutralisation measured against XBB1.5 and the most recent circulating XFG viruses using a cellular assay for neutralisation.^28^ We show that CD40.RBDv and CD40.Pan.CoV elicited neutralising activity against XBB1.5 and XFG. For example, given with or without adjuvant, in Delta or XBB1.5 convalescent animals, CD40.Pan.CoV elicited neutralising responses against XBB1.5 or XFG in 10/11 animals (Figure S13a). Moreover, we confirmed that ACE2-binding inhibition correlated with functional neutralisation measured using authentic virus (Figure S13b) and thus could be used here as a surrogate for neutralising antibody responses.

### Non-adjuvanted CD40.RBDv is a potent booster of immune memory responses in naive macaques primed with BNT162b2 mRNA vaccine

**Fig. 6:**
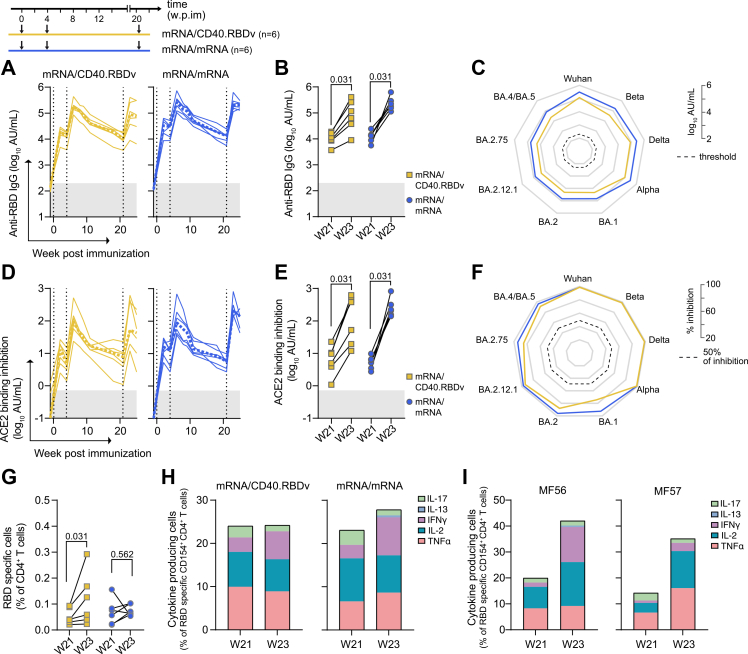
**SARS-CoV-2 specific Abs in mRNA/CD40.RBDv vaccinated NHPs.** IgG binding to Wuhan RBD (**A–C**) and inhibition of binding to human ACE2 (**D–F**) were analysed in serum samples of vaccinated animals. Individual values and median (thick dotted lines) were indicated for mRNA/CD40.RBDv (n = 6, yellow) and mRNA/mRNA (n = 6, blue) groups. Dotted vertical line represents vaccine administrations and grey band indicates positivity cutoff. (**B and E**) Ab responses before and after the CD40.RBDv or mRNA boost (i.e., last immunisation) were analysed among individual using Wilcoxon matched-pairs signed rank test. Radar plots represent the Ab titre in serum (**C**) or the percentage of inhibition of ACE2 binding by animal serum (**F**) against different VOC RBD at 4 weeks post vaccine injection. Coloured line represents the median group and dotted line indicates positivity cutoff (**C**) or 50% of inhibition (**F**). (**G**) Frequency of RBD-specific CD4^+^ T cells (CD154^+^/CD137^+^) in the total CD4^+^ T cell population for each mRNA/CD40.RBDv (n = 6, yellow) or mRNA/mRNA vaccinated animals (n = 6, blue). Time points were compared using the Wilcoxon signed rank test. (**H and I**) Frequency of cytokine producing cells in the RBD-specific CD4^+^ T cells (CD154^+^) for mRNA/CD40.RBDv and mRNA/mRNA vaccinated animals. Each bar indicates the median (**H**) of the 6 vaccinated animals or individual values (**I**) for the two animals within mRNA/CD40.RBDv group which exhibited increase of IL-2^+^ and IFNγ^+^ producing cells after the boost. Distribution of cytokines is indicated within each bar. W21: week 21 post immunisation, i.e., the baseline of CD40.RBDv or mRNA boost; W23: Two weeks post CD40.RBDv or third mRNA immunisation.

Considering that a large proportion of the general population is already infected and/or vaccinated with COVID-19 mRNA vaccines, we next evaluated the booster effect of the CD40.RBDv vaccine. First, naive macaques received two injections of BNT162b2 mRNA vaccine (Pfizer-BioNTech; n = 12; weeks 0 and 4). Two weeks following the 2nd mRNA vaccine, RBD-specific IgG titres in serum were 195,580 [IQR 126,921–346,576] AU/mL (Fig. 6A and Figure S14a). These responses decreased to 12,352 [8647–16,099] AU/mL at week 21. Analysis of anti-RBD IgG titre dynamics following vaccination revealed a biphasic linear decay model and estimated their persistence at 0.96–0.99 years (Figure S14b–c and Table S3). At week 21, animals received either one boost of non-adjuvanted CD40.RBDv (n = 6) or a third injection of mRNA vaccine (n = 6, Fig. 1C). Thus, RBD-specific IgG titres increased by 8.6-fold at week 23, two weeks after the CD40.RBDv boost (anti-RBD titres: 89,297 [IQR 30,474–294,707] AU/mL; median difference = 122,474, 95% CI [5191–386,703], p = 0.031), and by 14.9-fold after the mRNA boost (202,104 [144,936–373,594] AU/mL; median difference = 208,113, 95% CI [103,857–623,995], p = 0.031, Fig. 6B). CD40.RBDv boost, similarly to a 3rd mRNA shot, also induced anti-RBD IgG against different VOCs, including Omicron subvariants (Fig. 6C, Figure S15a and S15c). ACE2 binding inhibiting activity against RBD Wuhan was 97 [46–179]AU/ml (i.e., 99.88 [99.84–99.91]% of inhibition) two weeks following the 2nd mRNA injection and decreased at week 21 (5.70 [3.56–9.75] AU/mL; i.e., 92.24 [83.19–98.61] % of inhibition) (Fig. 6D). The CD40.RBDv boosted neutralising titres and neutralising activity against RBD Wuhan (215 [17.5–491.2] AU/mL and above 99%, respectively) (Fig. 6E–F), even if the magnitude of the response remained heterogeneous within the group (Fig. 6D). Post CD40.RBDv boost, percentages of inhibition reached up to 99% against Alpha, Beta, and Delta variants and 75–92% against Omicron VOCs (92% activity against BA.4/BA.5) (Fig. 6F, Figure S15b and S15d). Similarly, the 3rd mRNA vaccine dose enhanced neutralising activity, with inhibition percentages reaching up to 99% against Wuhan, Alpha, Beta, and Delta variants and 93–98% against Omicron VOCs (Fig. 6D–F and Figure S15d). IgG- and IgA-specific responses against RBD from Wuhan, Alpha, Beta, and Delta variants were detectable in mucosal fluids (nasopharyngeal fluids and BAL) (Figure S15e–f). CD40.RBDv boost significantly increased RBD-specific CD4^+^ T cell responses (median difference = 0.05, 95% CI [0.00–0.20], p = 0.031), in 3 NHPs out of 6 (Fig. 6G). The overall quality of the response for the group was not changed after the boost (Fig. 6H), but the IL-2^+^ and IFN-γ^+^ producing cells were increased after the CD40.RBDv boost in two T-cell responder animals (MF56 and MF57, Fig. 6I and Figure S16). In contrast, 3rd mRNA shot did not impact the RBD-specific CD4^+^ T cell responses (Fig. 6G and Figure S16).

### Multidimensional analysis of immunogenicity of CD40-based vaccines

**Fig. 7:**
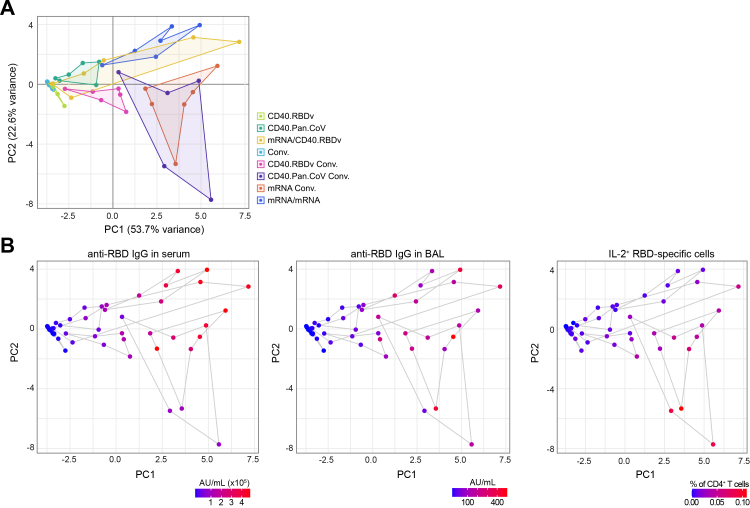
**Multidimensional unsupervised analysis of experimental groups and immunogenicity measures.** Principal component analysis (PCA) was conducted on antibody (serum and fluids) and T cell responses following vaccination in CD40.RBDv (light green), CD40.Pan.CoV (dark cyan), convalescent (light blue), CD40.RBDv convalescent (pink), CD40.Pan.CoV convalescent (purple), mRNA convalescent (orange), mRNA/CD40.RBDv (yellow) and mRNA/mRNA (blue) animals. (**A**) Individual plot connected to outline polygons highlighted in different colours. (**B**) PCA highlights the contribution of the following variables: serum (left) and BAL (middle) RBD specific IgG and IL-2^+^ CD4^+^ T cell response (right). Colours indicate the intensity of measured parameters (low in blue and high in red). Groups are connected in polygons.

We performed a Principal Component Analysis (PCA) based on immunogenicity data (antibody and T-cell responses) of CD40.RBDv, CD40.Pan.CoV and mRNA vaccines in naive and convalescent animals described above. Fig. 7 revealed distinct clustering between vaccinated and non-vaccinated convalescent animals (Fig. 7A), confirming the booster effect of CD40-based vaccines on residual immunity. Furthermore, we found an overlap between (i) convalescent animals vaccinated with CD40.Pan.CoV and those vaccinated with mRNA, and (ii) naive animals primed with mRNA and boosted either with a single non-adjuvanted CD40.RBDv dose or with a third mRNA dose, demonstrating that CD40-based vaccinated elicits immune responses equivalent to those induced by mRNA vaccination in both contexts (Fig. 7). Furthermore, PCA confirmed a high variability in vaccine response intensity within the mRNA/CD40.RBDv group, as previously noted (Fig. 6A, D and G). Variable contribution analysis revealed that vaccinated convalescent animals exhibited a stronger T-cell response, particularly IL-2-specific T cells, compared to naive animals vaccinated with CD40-targeting vaccines, either alone or in combination with mRNA vaccines (Fig. 7B and Figure S17). The same profile between groups was observed with anti-RBD specific IgG mucosal responses (Fig. 7B).

### Efficacy of CD40-targeting vaccines

**Fig. 8:**
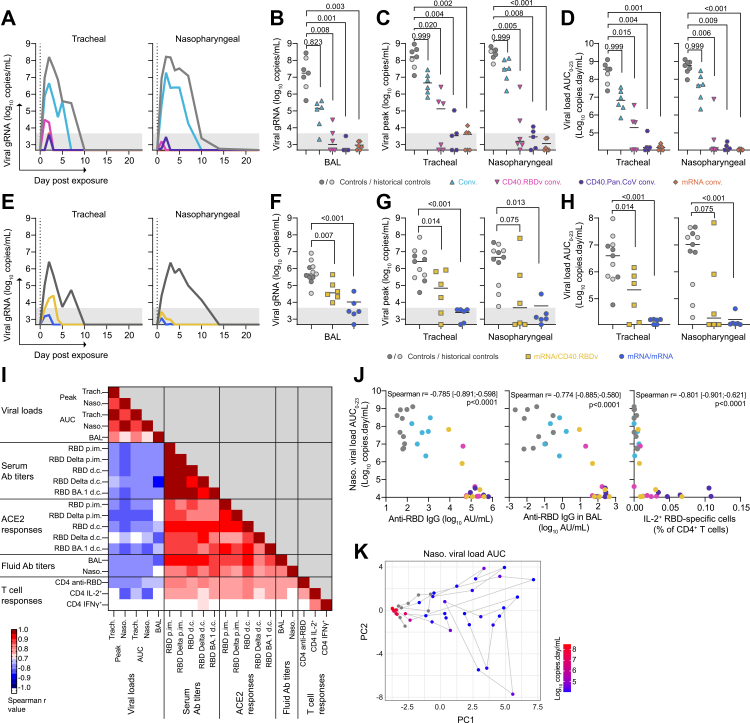
**SARS-CoV-2 RNA quantification after viral exposure.** Viral loads were measured after Delta B.1.617.2 (**A–D**) and Omicron BA.1 (**E–H**) exposure. (**A and E**) Tracheal and nasopharyngeal viral loads determined by RT-qPCR in animal of control (n = 7 and n = 11, grey), convalescent (n = 6, light blue), CD40.RBDv convalescent (n = 6, pink), CD40.Pan.CoV convalescent (n = 5, purple), mRNA convalescent (n = 6, orange), mRNA/CD40.RBDv vaccinated (n = 6, yellow) and mRNA/mRNA (n = 6, blue) groups. Lines indicate the median, dotted vertical line represents viral exposure and grey band indicates limit of quantification. (**B and F**) Viral load in BAL at 3 days post exposure. Symbol represents individual value and median value is indicated by horizontal bar. Grey band indicates limit of quantification. Viral peak (**C and G**) and area under the curve (AUC) (**D and H**) during the first 23 days of infection in tracheal (left) and nasopharyngeal (right) fluids. Viral loads, viral peak and AUC were compared between Delta B.1.617.2 exposed groups using Kruskal-Wallis test following Dunn's multiple comparisons (**B–D**), and Omicron BA.1 exposed groups using the two-tailed non-parametric Mann-Whitney test (**F–H**). (**I**) Correlation matrix between virological and immune parameters in CD40 vaccinated animals. The heatmap indicates the Spearman r values (Only values between −0.7 and −1, and 0.7 and 1 are coloured in the heatmap). p.im: week 2 post last immunisation; d.c: day of challenge. (**J**) Correlation between nasopharyngeal viral load AUC and IgG binding to Wuhan RBD in serum at week 2 post last immunisation (left) or IgG binding to Wuhan RBD in BAL at week 3 (center) or IL-2^+^ CD4^+^ T cell response against RBD after immunisation (right). The Spearman r [95% CI] and p (two-tailed) values are indicated. (**K**) Immunogenicity PCA highlights the contribution of the external variable: nasopharyngeal viral load AUC. Colours indicate the intensity of measured parameters (low in blue and high in red). Groups are connected in polygons.

Four weeks following a single administration of CD40.RBDv (n = 6), CD40.Pan.CoV (n = 5) or mRNA (n = 6) in SARS-CoV-2 convalescent macaques, or following a CD40.RBDv boost (n = 6) or a 3rd mRNA shot (n = 6) in mRNA vaccinated macaques, animals were challenged with a high dose (1.10^5^ TCID_50_) of Delta B.1.617.2 (Fig. 8A–D) or Omicron BA.1 virus (Fig. 8E–H), respectively via a combined intra-nasal and intra-tracheal route using a previously reported procedure.^17^ A total of eighteen SARS-CoV-2 naive animals and six convalescent animals were challenged as controls (Fig. 1C). After the Delta B.1.617.2 challenge, all naive and convalescent animals became infected as shown by the detection of viral genomic RNA (gRNA) in tracheal, nasopharyngeal swabs and BAL (Fig. 8A–D and Figure S18a–b). Both anti-CD40 vaccines provided partial protection compared to naive macaques, with significantly lower peak viral replication (gRNA) in BAL (median difference = 7.25 log_10_, 95% CI [6.43–8.14 log_10_], p = 0.008 for CD40.RBDv; and median difference = 7.24 log_10_, 95% CI [6.44–8.14 log_10_], p = 0.001 for CD40.Pan.CoV, Fig. 8B), tracheal (median difference = 8.21 log_10_, 95% CI [7.57–8.64 log_10_], p = 0.020 for CD40.RBDv and median difference = 8.21 log_10_, 95% CI [7.10–8.96 log_10_], p = 0.004 for CD40.Pan.CoV, Fig. 8C) and nasopharyngeal fluids (median difference = 8.53 log_10_, 95% CI [8.18–8.62 log_10_], p = 0.005 for CD40.RBDv; and median difference = 8.53 log_10_, 95% CI [8.19–8.62 log_10_], p = 0.008 for CD40.Pan.CoV, Fig. 8C). The levels of gRNA remained below the limit of quantification in upper respiratory tract samples in 7 of 11 of the animals vaccinated with CD40 targeting vaccines (Fig. 8C and Figure S18a–b). The efficacy of vaccination was also higher in the lower respiratory tract, as 9 of 11 vaccinated macaques were below the limit of quantification of gRNA in BAL at day 3 post-exposure whereas gRNA was detected in 5 of 6 non-vaccinated convalescent and all naive control animals (Fig. 8B). Overall, viral shedding as measured by the area under the curve (AUC) between days 0 and 23 post–challenge, was also significantly reduced (Fig. 8D). Analysis of viral sub-genomic RNA (sgRNA) in respiratory fluids showed that sgRNA was not detected in vaccinated animals in contrast to non-vaccinated animals (Figure S19a–c), Finally, quantification of gRNA and sgRNA levels in the respiratory fluids of mRNA-vaccinated animals revealed that the efficacy of mRNA vaccination was comparable to that of the CD40.Pan.CoV vaccine (Fig. 8A–D, Figure S18a–b and S19a–c).

Following Omicron BA.1 exposure, animals previously immunised with a prime-boost mRNA/CD40.RBDv regimen (Fig. 1C), exhibited detectable gRNA in BAL fluid 3 days post–challenge. However, significantly lower values were detected in vaccinated animals compared to control group (median difference = 5.61 log_10_, 95% CI [5.14–6.10 log_10_], p = 0.007; Fig. 8F). Two animals that received a 3rd mRNA injection also exhibited gRNA in their BAL. However, the efficacy of the 3rd mRNA dose was observed in all respiratory fluids (Fig. 8E–F). Peak of viral replication and AUC of viral load were also significantly reduced by 1.3-3 log_10_ in the upper respiratory tract of CD40.RBDv vaccinated animals as compared to controls, particularly in the tracheal compartment (Fig. 8G–H and Figure S18c–d). Notably, the heterogeneity of the immune responses elicited by the CD40.RBDv boost (Fig. 6, Figure S15 and S16) was replicated in the reduction of viral replication post–challenge, with 3 vaccinated animals exhibiting viral loads comparable to those of control animals (Fig. 8G–H and Figure S18c–d). Only one CD40.RBDv-vaccinated animal and no mRNA-vaccinated NHP exhibited sgRNA in their respiratory fluids (Figure S19d–f).

Spearman correlation analysis between immunological and virological parameters across CD40-vaccinated and challenged groups revealed that the magnitude of the vaccine-induced immune response correlated with the reduction of viral load (Fig. 8I–K). In particular, significant negative correlations were observed between nasopharynx AUC viral loads and serum anti-RBD IgG (r = −0.785, 95% CI [−0.891 to −0.598]), BAL anti-RBD IgG (r = −0.774, 95% CI [−0.885 to −0.580]) and the frequency of IL-2^+^ RBD-specific CD4^+^ T cells (r = −0.801, 95% CI [−0.901 to −0.621]), all measured after immunisation (p < 0.0001 for all comparisons) (Fig. 8J). The impact of anti-CD40 vaccination was further highlighted in Fig. 8K, which exhibited the distribution of nasopharyngeal AUC viral load as an external variable in the PCA of immunogenicity, previously performed (Fig. 7A). Indeed, animals exhibiting the highest AUC viral loads corresponded to those with the weakest vaccine-induced immune response.

## Discussion

The persistent evolution of SARS-CoV-2 VOCs, characterised by immune escape mutations, necessitates iterative vaccine updates to maintain efficacy. However, this approach is challenged by the concurrent circulation of multiple variants (GISAID Initiative https://gisaid.org/hcov19-variants/), the unpredictability of viral evolution,^30^ and waning public acceptance of frequent booster vaccinations. Consequently, the development of vaccines capable of conferring broader and more durable protection is paramount.

Leveraging the established principle of enhancing immune responses through targeted antigen delivery to DCs,^17^^,^^29^^,^^31^, ^32^, ^33^, ^34^, ^35^ we designed subunit vaccines incorporating mutated RBD sequences from SARS-CoV-2, either alone (CD40.RBDv) or in combination with a highly conserved nucleocapsid sequence (CD40.Pan.CoV), utilising CD40 targeting. In macaques, convalescent from prior SARS-CoV-2 infection, a single, non-adjuvanted dose of CD40.RBDv or CD40.Pan.CoV (18–24 months post-infection) induced sustained anti-RBD binding antibody responses and ACE2 binding inhibition against the original Wuhan strain, as well as Alpha, Beta, and Delta VOCs, that persisted for at least 53 weeks post-vaccination. We observed that ACE2-binding inhibition correlated with functional neutralisation measured using authentic virus (Figure S13b) and thus could be used here as a surrogate for neutralising antibody responses. SARS-CoV-2 naive macaques receiving a single dose of CD40.RBDv showed durable antibody responses, albeit at lower levels compared to vaccinated convalescent animals. Furthermore, we observed enhanced immunogenicity of CD40-targeted antigen delivery compared to non-targeted, isotype-matched antibody conjugates. Many subunit SARS-CoV-2 vaccines are administered in adjuvanted formulations to enhance immune responses,^36^ while the CD40 vaccines are immunogenic even without an adjuvant as a booster vaccine. These findings support the potential of non-adjuvanted CD40 vaccines to induce long-lived humoural immunity and broaden SARS-CoV-2 vaccine responses in both hybrid and vaccine-induced immunity settings.

The CD40 vaccine platform demonstrated a favourable safety profile, with local reactogenicity limited to local injection–site reactions, and transient inflammation observed only with the adjuvanted formulation (Figure S6a–c). Adverse events with the adjuvanted CD40 vaccine formulation, observed both here and in a Phase 1 human trial,^15^ were similar to those reported for other adjuvanted subunit vaccines.^36^^,^^37^

The majority of RBD mutations in SARS-CoV-2 variants have clustered around the ACE2 binding site. This strategic positioning facilitates both enhanced ACE2 receptor affinity, crucial for viral infectivity, and evasion of potent neutralising antibodies that typically disrupt the Spike-ACE2 interaction. Consequently, the humoural immune response to emerging variants is frequently compromised.^30^^,^^38^, ^39^, ^40^, ^41^, ^42^ At the time of CD40.RBDv vaccine design, escape-associated and infectivity-enhancing mutations were well characterised for Alpha, Beta, and Gamma VOCs,^43^^,^^44^ and for CD40.Pan.CoV, extended to Omicron BA.1. The mutated RBDv1 and RBDv2 sequences incorporated into the CD40.RBDv and CD40.Pan.CoV vaccines, respectively, encompass a significant proportion of these critical substitutions. Notably, residues such as K417N, N501Y, and T478K, which play pivotal roles in ACE2 binding, are conserved across virtually all significant SARS-CoV-2 variants.^30^ Furthermore, L452R exhibits intermittent substitutions since the emergence of Omicron variants, and E484K has recently reappeared in BA.2.86, JN.1, and subsequent circulating variants, including LP.8.1.^30^^,^^45^ The sustained conservation of these sites across diverse and highly immune-evasive variants underscores the essential role of these mutations in maintaining high-affinity ACE2 receptor binding.

Despite the emergence of additional mutations within the ACE2 interaction site, such as V445 P/H, G446S, S477N, F486 V/S/P, and Q498R, which have significantly diminished the neutralising efficacy of numerous monoclonal antibody therapies targeting class 1/2 RBD epitopes,^46^, ^47^, ^48^, ^49^ conserved epitopes within the RBDv1 and RBDv2 vaccine antigens may still continue to stimulate a neutralising polyclonal antibody response. This is supported by the strong antibody-mediated blockade of Spike Omicron BA.4/5 binding to ACE2 observed in CD40.Pan.CoV-vaccinated naive macaques. Furthermore, neutralisation assays against Omicron XBB.1.5 and XFG VOCs demonstrated that CD40.RBDv and CD40.Pan.CoV vaccines induced neutralising antibodies against these circulating strains at levels comparable to those elicited by an XBB1.5 mRNA vaccine in human, particularly in the contexts of hybrid and vaccine-boosted immunity (Figure S13a). Notably, among the 24 RBD residues directly involved in ACE2 contact, 16 are conserved within RBDv1 and RBDv2 across recent Spike VOCs. Furthermore, a substantial proportion of adjacent residues remain conserved, potentially contributing to the structural conformation of the class 1/2 neutralising region and facilitating effective antibody binding.

Beyond neutralising antibodies targeting class 1/2 sites that overlap with the ACE2 contact region, class 3 antibodies, including sotrovimab, bebtelovimab, and 47D11, target RBD regions exhibiting higher conservation across recent SARS-CoV-2 variants and the RBDv1/v2 vaccine antigens.^50^ Antibodies binding this region can impede ACE2 interactions and may also exert inhibitory effects through allosteric mechanisms or by disrupting viral fusion. Class 4 neutralising antibodies, such as CR3022, ADG20, and DH1047,^51^ recognise a more shielded RBD region, protected by inter-RBD contacts within the Spike trimer. The importance of maintaining inter-Spike protomer interactions, coupled with limited accessibility for antibody and the potentially lower neutralising potency of these antibodies, has resulted in high conservation of the class 4 region, with minimal evolution observed in SARS-CoV-2 VOCs. Given that the CD40.RBDv and CD40.PanCoV antigens present an RBD protein with an exposed class 4 region, these vaccines may facilitate the development of an enduring protective antibody response against this highly conserved epitope.

Our findings suggest a potential benefit of a heterologous CD40.RBDv boost in individuals previously immunised with mRNA vaccines. Over four years since the introduction of mRNA COVID-19 vaccines, extensive public surveys have demonstrated a decline in their effectiveness against moderate and severe disease over time.^52^ Furthermore, the long-term efficacy of recent monovalent mRNA vaccine platforms, designed to target specific circulating VOCs, remains to be fully established due to the continuous emergence of novel VOCs harbouring immune escape mutations.^53^ We observed a substantial decrease in binding and neutralising antibody titres four months after a second BNT162b2 mRNA vaccine dose in naive macaques, and our mathematical model predicted a persistence of anti-RBD IgG titres for 0.99 years. Notably, a single non-adjuvanted CD40.RBDv boost significantly increased (8.6-fold) the magnitude and breadth of neutralising antibody responses, achieving up to 99% inhibition against Alpha, Beta, and Delta variants, and 92% against Omicron BA.4/BA.5. Thus, a CD40.RBDv boost induced immune responses similar to those elicited by a third mRNA dose. Moreover, the increase in immune responses following CD40.RBDv boosting appeared greater than that achieved with other adjuvanted subunit vaccine in a similar boosting context.^54^ Prior studies have indicated that heterologous booster vaccines may offer enhanced protection compared to homologous boosting.^55^, ^56^, ^57^ This hypothesis is currently being investigated in an ongoing Phase 1/2a clinical trial (ANRS 0407s - LKV.Cov40; NCT06255626), which is evaluating the booster effect of a single CD40.RBDv administration in volunteers previously vaccinated with a primary series and booster doses of mRNA vaccines.

Prior investigations have established that hybrid immunity to SARS-CoV-2 confers greater durability and reduced reinfection risk compared to mRNA booster vaccinations.^53^ We demonstrated here the superior durability of CD40 vaccines compared to mRNA vaccines. In context of hybrid immunity, our mathematical model predicted that anti-RBD IgG titres persisted for approximately 0.7 years after a single mRNA dose, compared with 5.28–39 years following CD40 vaccination, although these estimates should be interpreted cautiously given the modelling assumptions. Moreover, 42 weeks after CD40.Pan.CoV vaccination, vaccinated convalescent NHP still displayed neutralising activity, including against recent SARS-CoV-2 strains such as XBB.1.5 and XFG. The precise correlates of protection underlying hybrid immunity remain incompletely elucidated. Beyond neutralising antibodies, T-cell memory responses are critical for establishing sustained immune memory. Studies have demonstrated that SARS-CoV-2 infection and vaccination induce specific T-cell priming, with robust central memory T-cell induction contributing to durable protective immunity in adults.^58^, ^59^, ^60^, ^61^ In this study, we demonstrated that CD40 vaccine boosts increased the frequency of memory RBD-specific CD4^+^ T cells in convalescent animals and even five months after mRNA priming. These findings corroborate the potential of the CD40 platform to induce vaccine-specific T-cell responses, as previously shown in various preclinical models^17^^,^^19^^,^^29^^,^^62^ and Phase 1/2a clinical trials.^15^ We did not observe a substantial increase in SARS-CoV-2-specific CD8^+^ T cells compared to baseline responses in convalescent or mRNA-vaccinated animals. This contrasts with our recent findings in a humanised mouse model, where CD40.AMV selectively enriched long-lived Spike- and nucleocapsid-specific human CD8^+^ T cell progenitors with stem-cell-like memory (Tscm) properties, while mRNA BNT162b2 predominantly induced effector memory CD8^+^ T cells.^19^ Nevertheless, we confirmed the immunogenicity of the highly conserved Nep2 sequence (>93% conservation across Sarbecoviruses^18^) in recipients of the CD40.Pan.CoV vaccine. A potential advantage of a vaccine incorporating a nucleocapsid sequence is the induction of responses against more conserved regions across SARS-CoV-2 variants, which could be particularly beneficial in the event of immune escape by neutralising antibodies, and potentially priming T-cell memory against non-COVID-19 Sarbecoviruses.

Recent investigations in human cohorts and preclinical models have revealed that while current mRNA vaccines effectively induce robust serum neutralising antibody responses, the humoural immunity within the nasal mucosa remains suboptimal.^63^, ^64^, ^65^, ^66^, ^67^ In this study, we demonstrated that both CD40 vaccines elicited RBD-specific IgG and IgA in upper and lower respiratory mucosa against RBD from Alpha, Beta, and Delta VOCs. These responses were observed 3–4 weeks post-vaccination in both naive and pre-immunised animals, including convalescents and those primed with mRNA vaccines. Although a direct quantitative comparison of RBD-specific IgG levels across vaccine groups was not performed, our data suggest that both CD40 vaccines elicited higher mucosal responses (IgG and IgA) in the context of hybrid immunity. These findings align with human data indicating that hybrid immunity may be more effective in generating mucosal responses compared to vaccination alone.^67^^,^^68^ The development of safe, affordable, and efficient mucosal vaccines remains a critical goal for enhancing protection against respiratory infections. However, the practicality of a systemic heterologous boost as a strategy for controlling breakthrough infections warrants consideration. Given that after five years of SARS-CoV-2 circulation, most individuals likely possess a combination of immunity from prior vaccination and/or infection, systemic boosting may offer a more feasible approach. Consequently, further investigations comparing systemic and mucosal administration of diverse vaccine platforms as booster strategies within the context of hybrid immunity are necessary.^69^

This study acknowledges limitations. Due to logistic constraints, a direct, head-to-head comparison of vaccine strategies was not feasible. Instead, a sequential evaluation was conducted under varying conditions, which may introduce confounding factors. The assessment of mucosal responses was limited to a relatively short timeframe (3–4 weeks post-vaccination), precluding any conclusions regarding the long-term durability of mucosal responses. We did not definitively establish the origin of SARS-CoV-2 IgG binding antibodies detected in the respiratory tracts. It remains unclear whether these antibodies are indicative of a true mucosal vaccine response or a result of systemic IgG translocation. Furthermore, it worth mentioning that results obtained by the model about the durability of vaccine-induced IgG binding response depends on the modelling choice and can be impacted by bias resulting from model misspecification. Notably, the lowest longevity identified in mRNA groups strongly depends on the linear model used constrained by the short follow-up in these groups. In addition, the relatively limited number of animals per group may limit direct extrapolation of these findings to humans.

We have investigated CD40 vaccines with varying antigenic compositions, coupled with the evaluation of their immunogenicity and protective efficacy against viral challenges in both pre-immunised and naive animals. This approach enabled the delineation of distinct immune response profiles under these diverse conditions and their respective contributions to protection against viral challenge. We demonstrated the protective activity of a single boost with CD40.RBDv or CD40.Pan.CoV vaccines in pre-immunised animals. Notably, boosted animals exhibited minimal or undetectable viral replication in the nasal mucosa and BAL, which may contribute to reduced viral shedding, whereas viral replication was readily detectable in nearly all naive or non-vaccinated convalescent controls. Moreover, protective efficacy was further supported by the absence of subgenomic viral RNA in CD40-vaccinated animals. Analysis of immune response contributions revealed that pre-immunised vaccinated animals displayed a robust T-cell response, particularly characterised by IL-2-specific T cells, compared to naive animals vaccinated with CD40-targeting vaccines, either alone or in combination with mRNA vaccines. This occurred despite comparable levels of anti-RBD-specific IgG mucosal responses across groups. Furthermore, post–challenge (Delta or Omicron) nasopharyngeal area under the curve (AUC) viral loads exhibited an inverse correlation with the magnitude of serum and BAL RBD-antibody responses, as well as the frequency of RBD-specific CD4^+^ T cells, measured after single CD40.RBDv or CD40.Pan.CoV boosts. These data contribute to the identification of SARS-CoV-2 correlates of protection and extend other studies investigating mRNA vaccine correlates of protection.^70^^,^^71^ Importantly, single administrations of CD40-targeted SARS-CoV-2 antigens without adjuvant induced durable immunogenicity and protective activity in macaque models, supporting further evaluation of this platform in human clinical trials.

Beyond SARS-CoV-2, the CD40-targeting approach represents a modular platform that has already shown applicability across multiple pathogens (HIV,^15^ HPV^72^), including emerging viruses (Nipah^29^), which may facilitate rapid antigen adaptation while preserving a common delivery framework.

## Contributors

Conceptualisation: RLG, RM, MC, YL, VG.

Data Curation: RM, CH, MG.

Formal Analysis: RM, PM.

Funding Acquisition: MC, YL.

Investigation: LB, LP, WG, VM, ASG, IS, DP.

Methodology: MA, RT, MP, PM.

Project Administration: RM, CH, MG, MC.

Resources: MC, CF, MS, SC, VC, FR, SZ, GZ, MARW.

Supervision: NDB, ASG, FR, OS.

Validation: MCa, NDB, ASG, DP, OS.

Visualisation: RM, MA.

Writing–Original Draft: YL, RM, RLG.

Writing–Review & Editing: all.

Access and verification of underlying data: RM, LB, ASG.

All authors read and approved the final version of the manuscript.

## Data sharing statement

Data that support the findings of this study are provided in the source data file of this paper. Source data are provided with this paper.

## Declaration of interests

The authors S.Z., G.Z., S.C, M.S., V.G., M.C., and Y.L., are named inventors on patent applications on CD40.RBDv and/or CD40.Pan.CoV vaccines held by Inserm Transfert. Inserm Transfert provided a licence for CD40 targeting vaccines to the biotech company LinKinVax/EnnoDC. The remaining authors declare no competing interests.
