# Supplementary material for: Immunogenicity and protective efficacy on non-adjuvanted CD40-targeting SARS-CoV-2 vaccines in non-human primates
Source: eBioMedicine. 2026 Jul 1;129:106361. doi: 10.1016/j.ebiom.2026.106361 (PMC13347779; doi:10.1016/j.ebiom.2026.106361)
Supplement: Supplementary Figures and Tables [file mmc2.pdf]

Supplementary materials

Immunogenicity and protective efficacy on non-adjuvanted CD40-targeting SARS-CoV-2 vaccines in non-human primates

Marlin R. et al.

List of contents:

Supplementary Figure S1	p.2
Supplementary Figure S2	p.3
Supplementary Figure S3	p.4
Supplementary Figure S4	p.5
Supplementary Figure S5	p.6
Supplementary Figure S6	p.7
Supplementary Figure S7	p.8
Supplementary Figure S8	p.9
Supplementary Figure S9	p.10
Supplementary Figure S10	p.11
Supplementary Figure S11	p.12
Supplementary Figure S12	p.13
Supplementary Figure S13	p.14
Supplementary Figure S14	p.15
Supplementary Figure S15	p.16
Supplementary Figure S16	p.17
Supplementary Figure S17	p.18
Supplementary Figure S18	p.19
Supplementary Figure S19	p.20
Supplementary Table SI	p.21
Supplementary Table SII	p.22-24
Supplementary Table SIII	p.25

Supplementary Figures

A

RBDv1 with K417N, E484K and N501Y

	RBD																																				
	332	339	346	356	368	371	373	375	376	403	405	408	417	435	440	444	445	446	450	452	455	456	460	475	477	478	481	483	484	486	487	490	493	496	498	501	505	521
**Wuhan**	**I**	**G**	**R**	**K**	**L**	**S**	**S**	**S**	**T**	**R**	**D**	**R**	**K**	**A**	**N**	**K**	**V**	**G**	**N**	**L**	**L**	**F**	**N**	**A**	**S**	**T**	**N**	**V**	**E**	**F**	**N**	**F**	**Q**	**G**	**Q**	**N**	**Y**	**P**
Alpha B.1.1.7 (20I, V1)																																				N501Y		
Beta B.1.351 (20H, V2)													K417N																E484K							N501Y		
Gamma P.1 (20J, V3)													K417T																E484K							N501Y		
Delta B.1.617.2 (21A, 21I; 21J)																				L452R						T478K												
Kappa B.1.617.1 (21B)																				L452R									E484Q									
Epsilon (21C)																				L452R																		
Eta B.1.525 (21D), Iota B.1.526 (21F), Zeta P.2																													E484K									
Lambda C.37 (21G)																				L452Q												F490S						
Mu B.1.621 (21H)			R346K																										E484K							N501Y		
Omicron BA.1 (21K)		G339D				S371L	S373P	S375F					K417N		N440K			G446S							S477N	T478K			E484A				Q493R	G496S	Q498R	N501Y	Y505H	
Omicron BA.2 (21L)		G339D				S371F	S373P	S375F	T376A		D405N	R408S	K417N		N440K										S477N	T478K			E484A				Q493R		Q498R	N501Y	Y505H	
Omicron BA.4 (22A), BA.5 (22B)		G339D				S371F	S373P	S375F	T376A		D405N	R408S	K417N		N440K					L452R					S477N	T478K			E484A	F486V					Q498R	N501Y	Y505H	
Omicron BA.2.12.1 (22C)		G339D				S371F	S373P	S375F	T376A		D405N	R408S	K417N		N440K					L452Q					S477N	T478K			E484A				Q493R		Q498R	N501Y	Y505H	
Omicron BA.2.75 (22D)		G339H				S371F	S373P	S375F	T376A		D405N	R408S	K417N		N440K			G446S					N460K		S477N	T478K			E484A						Q498R	N501Y	Y505H	
Omicron BQ.1 (22E)		G339D				S371F	S373P	S375F	T376A		D405N	R408S	K417N		N440K	K444T				L452R			N460K		S477N	T478K			E484A	F486V					Q498R	N501Y	Y505H	
Omicron BQ.1.1		G339D	R346T			S371F	S373P	S375F	T376A		D405N	R408S	K417N		N440K					L452R			N460K		S477N	T478K			E484A	F486V					Q498R	N501Y	Y505H	
Omicron BA.4.6, BF.7		G339D	R346T			S371F	S373P	S375F	T376A		D405N	R408S	K417N		N440K					L452R					S477N	T478K			E484A	F486V					Q498R	N501Y	Y505H	
Omicron BA.2.75.2		G339H	R346T			S371F	S373P	S375F	T376A		D405N	R408S	K417N		N440K			G446S					N460K		S477N	T478K			E484A	F486S					Q498R	N501Y	Y505H	
Omicron XBB (22F)		G339H	R346T		L368I	S371F	S373P	S375F	T376A		D405N	R408S	K417N		N440K		V445P	G446S					N460K		S477N	T478K			E484A	F486S		F490S			Q498R	N501Y	Y505H	
Omicron XBB.1.5 (23A), XBB.1.9 (23D)		G339H	R346T		L368I	S371F	S373P	S375F	T376A		D405N	R408S	K417N		N440K		V445P	G446S					N460K		S477N	T478K			E484A	F486P		F490S			Q498R	N501Y	Y505H	
Omicron XBB.1.16 (23B)		G339H	R346T		L368I	S371F	S373P	S375F	T376A		D405N	R408S	K417N		N440K		V445P	G446S					N460K		S477N	T478R			E484A	F486P		F490S			Q498R	N501Y	Y505H	
Omicron CH.1.1 (23C)		G339H	R346T			S371F	S373P	S375F	T376A		D405N	R408S	K417N		N440K	K444T		G446S		L452R			N460K		S477N	T478K			E484A	F486S					Q498R	N501Y	Y505H	
Omicron XBB.2.3 (23E)		G339H	R346T		L368I	S371F	S373P	S375F	T376A		D405N	R408S	K417N		N440K		V445P	G446S					N460K		S477N	T478K			E484A	F486P		F490S			Q498R	N501Y	Y505H	P521S
Omicron EG.5.1 (23F)		G339H	R346T		L368I	S371F	S373P	S375F	T376A		D405N	R408S	K417N		N440K		V445P	G446S				F456L	N460K		S477N	T478K			E484A	F486P		F490S			Q498R	N501Y	Y505H	
Omicron XBB.1.5.70 (23G)		G339H	R346T		L368I	S371F	S373P	S375F	T376A		D405N	R408S	K417N		N440K		V445P	G446S			L455F	F456L	N460K		S477N	T478K			E484A	F486P		F490S			Q498R	N501Y	Y505H	
Omicron HK.3 (23H)		G339H	R346T		L368I	S371F	S373P	S375F	T376A		D405N	R408S	K417N		N440K		V445P	G446S			L455F	F456L	N460K		S477N	T478K			E484A	F486P/S		F490S			Q498R	N501Y	Y505H	
Omicron BA.2.86 (23I)	I332V	G339H		K356T		S371F	S373P	S375F	T376A	R403K	D405N	R408S	K417N		N440K		V445H	G446S	N450D	L452W			N460K		S477N	T478K	N481K	V483-	E484K	F486P					Q498R	N501Y	Y505H	
Omicron FL.1.5.1		G339H	R346T		L368I	S371F	S373P	S375F	T376A		D405N	R408S	K417N		N440K		V445P	G446S				F456L	N460K		S477N	T478R			E484A	F486P		F490S			Q498R	N501Y	Y505H	
Omicron HV.1		G339H	R346T		L368I	S371F	S373P	S375F	T376A		D405N	R408S	K417N		N440K		V445P	G446S		L452R		F456L	N460K		S477N	T478K			E484A	F486P		F490S			Q498R	N501Y	Y505H	
Omicron JD.1.1		G339H	R346T		L368I	S371F	S373P	S375F	T376A		D405N	R408S	K417N		N440K		V445P	G446S			L455F	F456L	N460K	A475V	S477N	T478K			E484A	F486P		F490S			Q498R	N501Y	Y505H	
Omicron JN.1 (24A)	I332V	G339H		K356T		S371F	S373P	S375F	T376A	R403K	D405N	R408S	K417N		N440K		V445H	G446S	N450D	L452W	L455S		N460K		S477N	T478K	N481K	V483-	E484K	F486P					Q498R	N501Y	Y505H	
Omicron JN.1.11.1 (24B)	I332V	G339H		K356T		S371F	S373P	S375F	T376A	R403K	D405N	R408S	K417N		N440K		V445H	G446S	N450D	L452W	L455S	F456L	N460K		S477N	T478K	N481K	V483-	E484K	F486P					Q498R	N501Y	Y505H	
Omicron KP.2, LB.1	I332V	G339H	R346T	K356T		S371F	S373P	S375F	T376A	R403K	D405N	R408S	K417N		N440K		V445H	G446S	N450D	L452W	L455S	F456L	N460K		S477N	T478K	N481K	V483-	E484K	F486P					Q498R	N501Y	Y505H	
Omicron KP.3 (24C)	I332V	G339H		K356T		S371F	S373P	S375F	T376A	R403K	D405N	R408S	K417N		N440K		V445H	G446S	N450D	L452W	L455S	F456L	N460K		S477N	T478K	N481K	V483-	E484K	F486P			Q493E		Q498R	N501Y	Y505H	
Omicron XEC (24F)	I332V	G339H		K356T		S371F	S373P	S375F	T376A	R403K	D405N	R408S	K417N		N440K		V445H	G446S	N450D	L452W	L455S	F456L	N460K		S477N	T478K	N481K	V483-	E484K	F486P			Q493E		Q498R	N501Y	Y505H	
Omicron LP.8.1 (25A)	I332V	G339H	R346T	K356T		S371F	S373P	S375F	T376A	R403K	D405N	R408S	K417N		N440K		V445R	G446S	N450D	L452W	L455S	F456L	N460K		S477N	T478K	N481K	V483-	E484K	F486P			Q493E		Q498R	N501Y	Y505H	
Omicron NB.1.8.1 (25B)	I332V	G339H		K356T		S371F	S373P	S375F	T376A	R403K	D405N	R408S	K417N	A435S	N440K		V445H	G446S	N450D	L452W	L455S	F456L	N460K		S477N	T478I	N481K	V483-	E484K	F486P			Q493E		Q498R	N501Y	Y505H	
Omicron XFG (25C)	I332V	G339H	R346T	K356T		S371F	S373P	S375F	T376A	R403K	D405N	R408S	K417N		N440K	K444R	V445R	G446S	N450D	L452W	L455S	F456L	N460K		S477N	T478K	N481K	V483-	E484K	F486P	N487D		Q493E		Q498R	N501Y	Y505H	

B

RBDv2 with K417N, L452R, T478K, E484Q and N501Y

	RBD																																				
	332	339	346	356	368	371	373	375	376	403	405	408	417	435	440	444	445	446	450	452	455	456	460	475	477	478	481	483	484	486	487	490	493	496	498	501	505	521
**Wuhan**	**I**	**G**	**R**	**K**	**L**	**S**	**S**	**S**	**T**	**R**	**D**	**R**	**K**	**A**	**N**	**K**	**V**	**G**	**N**	**L**	**L**	**F**	**N**	**A**	**S**	**T**	**N**	**V**	**E**	**F**	**N**	**F**	**Q**	**G**	**Q**	**N**	**Y**	**P**
Alpha B.1.1.7 (20I, V1)																																				N501Y		
Beta B.1.351 (20H, V2)													K417N																E484K							N501Y		
Gamma P.1 (20J, V3)													K417T																E484K							N501Y		
Delta B.1.617.2 (21A, 21I; 21J)																				L452R						T478K												
Kappa B.1.617.1 (21B)																				L452R									E484Q									
Epsilon (21C)																				L452R																		
Eta B.1.525 (21D), Iota B.1.526 (21F), Zeta P.2																													E484K									
Lambda C.37 (21G)																				L452Q												F490S						
Mu B.1.621 (21H)			R346K																										E484K							N501Y		
Omicron BA.1 (21K)		G339D				S371L	S373P	S375F					K417N		N440K			G446S							S477N	T478K			E484A				Q493R	G496S	Q498R	N501Y	Y505H	
Omicron BA.2 (21L)		G339D				S371F	S373P	S375F	T376A		D405N	R408S	K417N		N440K										S477N	T478K			E484A				Q493R		Q498R	N501Y	Y505H	
Omicron BA.4 (22A), BA.5 (22B)		G339D				S371F	S373P	S375F	T376A		D405N	R408S	K417N		N440K					L452R					S477N	T478K			E484A	F486V					Q498R	N501Y	Y505H	
Omicron BA.2.12.1 (22C)		G339D				S371F	S373P	S375F	T376A		D405N	R408S	K417N		N440K					L452Q					S477N	T478K			E484A				Q493R		Q498R	N501Y	Y505H	
Omicron BA.2.75 (22D)		G339H				S371F	S373P	S375F	T376A		D405N	R408S	K417N		N440K			G446S					N460K		S477N	T478K			E484A						Q498R	N501Y	Y505H	
Omicron BQ.1 (22E)		G339D				S371F	S373P	S375F	T376A		D405N	R408S	K417N		N440K	K444T				L452R			N460K		S477N	T478K			E484A	F486V					Q498R	N501Y	Y505H	
Omicron BQ.1.1		G339D	R346T			S371F	S373P	S375F	T376A		D405N	R408S	K417N		N440K					L452R			N460K		S477N	T478K			E484A	F486V					Q498R	N501Y	Y505H	
Omicron BA.4.6, BF.7		G339D	R346T			S371F	S373P	S375F	T376A		D405N	R408S	K417N		N440K					L452R					S477N	T478K			E484A	F486V					Q498R	N501Y	Y505H	
Omicron BA.2.75.2		G339H	R346T			S371F	S373P	S375F	T376A		D405N	R408S	K417N		N440K			G446S					N460K		S477N	T478K			E484A	F486S					Q498R	N501Y	Y505H	
Omicron XBB (22F)		G339H	R346T		L368I	S371F	S373P	S375F	T376A		D405N	R408S	K417N		N440K		V445P	G446S					N460K		S477N	T478K			E484A	F486S		F490S			Q498R	N501Y	Y505H	
Omicron XBB.1.5 (23A), XBB.1.9 (23D)		G339H	R346T		L368I	S371F	S373P	S375F	T376A		D405N	R408S	K417N		N440K		V445P	G446S					N460K		S477N	T478K			E484A	F486P		F490S			Q498R	N501Y	Y505H	
Omicron XBB.1.16 (23B)		G339H	R346T		L368I	S371F	S373P	S375F	T376A		D405N	R408S	K417N		N440K		V445P	G446S					N460K		S477N	T478R			E484A	F486P		F490S			Q498R	N501Y	Y505H	
Omicron CH.1.1 (23C)		G339H	R346T			S371F	S373P	S375F	T376A		D405N	R408S	K417N		N440K	K444T		G446S		L452R			N460K		S477N	T478K			E484A	F486S					Q498R	N501Y	Y505H	
Omicron XBB.2.3 (23E)		G339H	R346T		L368I	S371F	S373P	S375F	T376A		D405N	R408S	K417N		N440K		V445P	G446S					N460K		S477N	T478K			E484A	F486P		F490S			Q498R	N501Y	Y505H	P521S
Omicron EG.5.1 (23F)		G339H	R346T		L368I	S371F	S373P	S375F	T376A		D405N	R408S	K417N		N440K		V445P	G446S				F456L	N460K		S477N	T478K			E484A	F486P		F490S			Q498R	N501Y	Y505H	
Omicron XBB.1.5.70 (23G)		G339H	R346T		L368I	S371F	S373P	S375F	T376A		D405N	R408S	K417N		N440K		V445P	G446S			L455F	F456L	N460K		S477N	T478K			E484A	F486P		F490S			Q498R	N501Y	Y505H	
Omicron HK.3 (23H)		G339H	R346T		L368I	S371F	S373P	S375F	T376A		D405N	R408S	K417N		N440K		V445P	G446S			L455F	F456L	N460K		S477N	T478K			E484A	F486P/S		F490S			Q498R	N501Y	Y505H	
Omicron BA.2.86 (23I)	I332V	G339H		K356T		S371F	S373P	S375F	T376A	R403K	D405N	R408S	K417N		N440K		V445H	G446S	N450D	L452W			N460K		S477N	T478K	N481K	V483-	E484K	F486P					Q498R	N501Y	Y505H	
Omicron FL.1.5.1		G339H	R346T		L368I	S371F	S373P	S375F	T376A		D405N	R408S	K417N		N440K		V445P	G446S				F456L	N460K		S477N	T478R			E484A	F486P		F490S			Q498R	N501Y	Y505H	
Omicron HV.1		G339H	R346T		L368I	S371F	S373P	S375F	T376A		D405N	R408S	K417N		N440K		V445P	G446S		L452R		F456L	N460K		S477N	T478K			E484A	F486P		F490S			Q498R	N501Y	Y505H	
Omicron JD.1.1		G339H	R346T		L368I	S371F	S373P	S375F	T376A		D405N	R408S	K417N		N440K		V445P	G446S			L455F	F456L	N460K	A475V	S477N	T478K			E484A	F486P		F490S			Q498R	N501Y	Y505H	
Omicron JN.1 (24A)	I332V	G339H		K356T		S371F	S373P	S375F	T376A	R403K	D405N	R408S	K417N		N440K		V445H	G446S	N450D	L452W	L455S		N460K		S477N	T478K	N481K	V483-	E484K	F486P					Q498R	N501Y	Y505H	
Omicron JN.1.11.1 (24B)	I332V	G339H		K356T		S371F	S373P	S375F	T376A	R403K	D405N	R408S	K417N		N440K		V445H	G446S	N450D	L452W	L455S	F456L	N460K		S477N	T478K	N481K	V483-	E484K	F486P					Q498R	N501Y	Y505H	
Omicron KP.2, LB.1	I332V	G339H	R346T	K356T		S371F	S373P	S375F	T376A	R403K	D405N	R408S	K417N		N440K		V445H	G446S	N450D	L452W	L455S	F456L	N460K		S477N	T478K	N481K	V483-	E484K	F486P					Q498R	N501Y	Y505H	
Omicron KP.3 (24C)	I332V	G339H		K356T		S371F	S373P	S375F	T376A	R403K	D405N	R408S	K417N		N440K		V445H	G446S	N450D	L452W	L455S	F456L	N460K		S477N	T478K	N481K	V483-	E484K	F486P			Q493E		Q498R	N501Y	Y505H	
Omicron XEC (24F)	I332V	G339H		K356T		S371F	S373P	S375F	T376A	R403K	D405N	R408S	K417N		N440K		V445H	G446S	N450D	L452W	L455S	F456L	N460K		S477N	T478K	N481K	V483-	E484K	F486P			Q493E		Q498R	N501Y	Y505H	
Omicron LP.8.1 (25A)	I332V	G339H	R346T	K356T		S371F	S373P	S375F	T376A	R403K	D405N	R408S	K417N		N440K		V445R	G446S	N450D	L452W	L455S	F456L	N460K		S477N	T478K	N481K	V483-	E484K	F486P			Q493E		Q498R	N501Y	Y505H	
Omicron NB.1.8.1 (25B)	I332V	G339H		K356T		S371F	S373P	S375F	T376A	R403K	D405N	R408S	K417N	A435S	N440K		V445H	G446S	N450D	L452W	L455S	F456L	N460K		S477N	T478I	N481K	V483-	E484K	F486P			Q493E		Q498R	N501Y	Y505H	
Omicron XFG (25C)	I332V	G339H	R346T	K356T		S371F	S373P	S375F	T376A	R403K	D405N	R408S	K417N		N440K	K444R	V445R	G446S	N450D	L452W	L455S	F456L	N460K		S477N	T478K	N481K	V483-	E484K	F486P	N487D		Q493E		Q498R	N501Y	Y505H	

Figure S1. Mutation list for RBDv1 and RBDv2. Conservation between RBDv1 contained in the CD40.RBDv vaccine (**A**) or RBDv2 contained in the CD40.Pan.CoV vaccine (**B**) and RBD from SARS-CoV-2 Wuhan and variants of concerns.

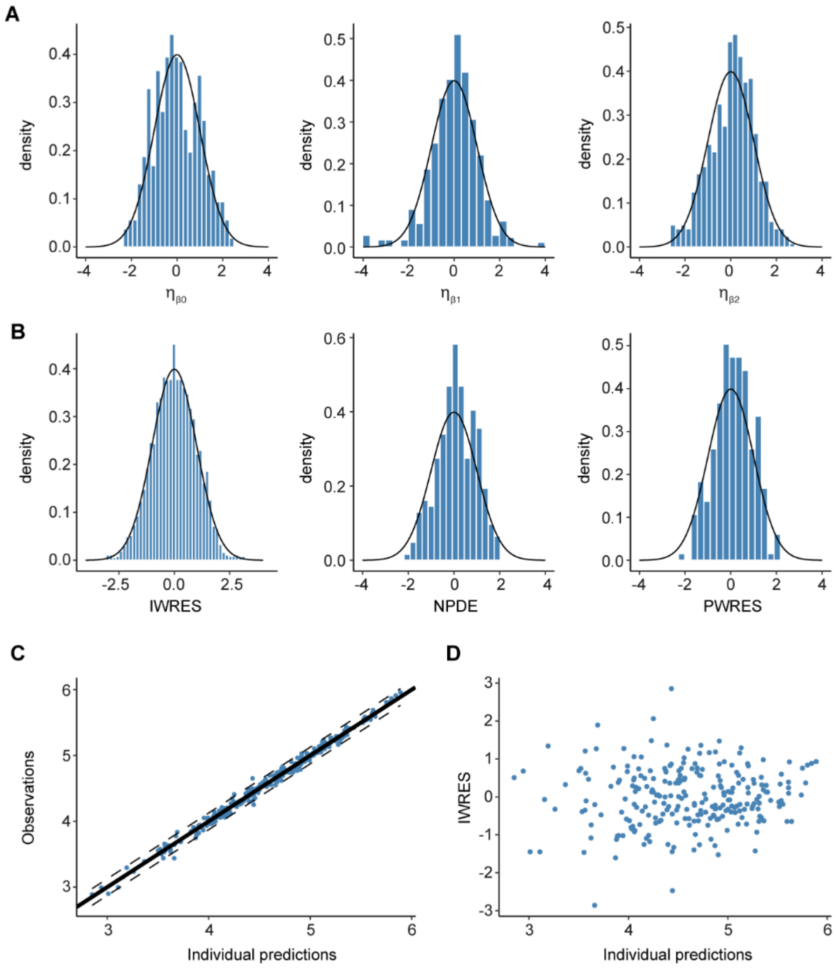

Figure S2. Statistical validation of the model estimated on anti-SARS-CoV-2 antibodies binding to Wuhan RBD. (A) Distribution of random effects on the model parameters β_0 (left), β_1(middle) and β_2 (right). Shrinkage [i.e. $1 - sd(\eta)/\omega$, with $sd(\eta)$ the empirical SD of random effects and ω the estimated SD of the population parameter] were estimated at -0·375%, -4·83%, and 1·36% for β_0, β_1 and β_2 respectively. P-values of Shapiro-Wilk tests {H0: random effects are normally distributed} were estimated at 7·76e-1, 1·34e-2 and 7·48e-1, respectively. **(B)** Distribution of residuals for individual weighted residuals (IWRES, left), normalized prediction distribution errors (NPDE, middle) and population weighted residuals (PWRES, right). P-values of Shapiro-Wilk test {H0: residuals are normally distributed} were estimated at 8·89e-1, 8.69e-1, 9·07e-1 (from left to right). **(A-B)** Solid black lines represent the theoretical distributions and histogram bars the empirical ones. **(C)** Observation versus individual predictions plot. A percentage of 2·01% of outliers for the 90%CI was found. Blue dots correspond to observed data, the solid black line represent the identity line (observation = prediction), and the dashed lines represent the 90%CI. **(D)** Scatterplot of residuals for individual predictions.

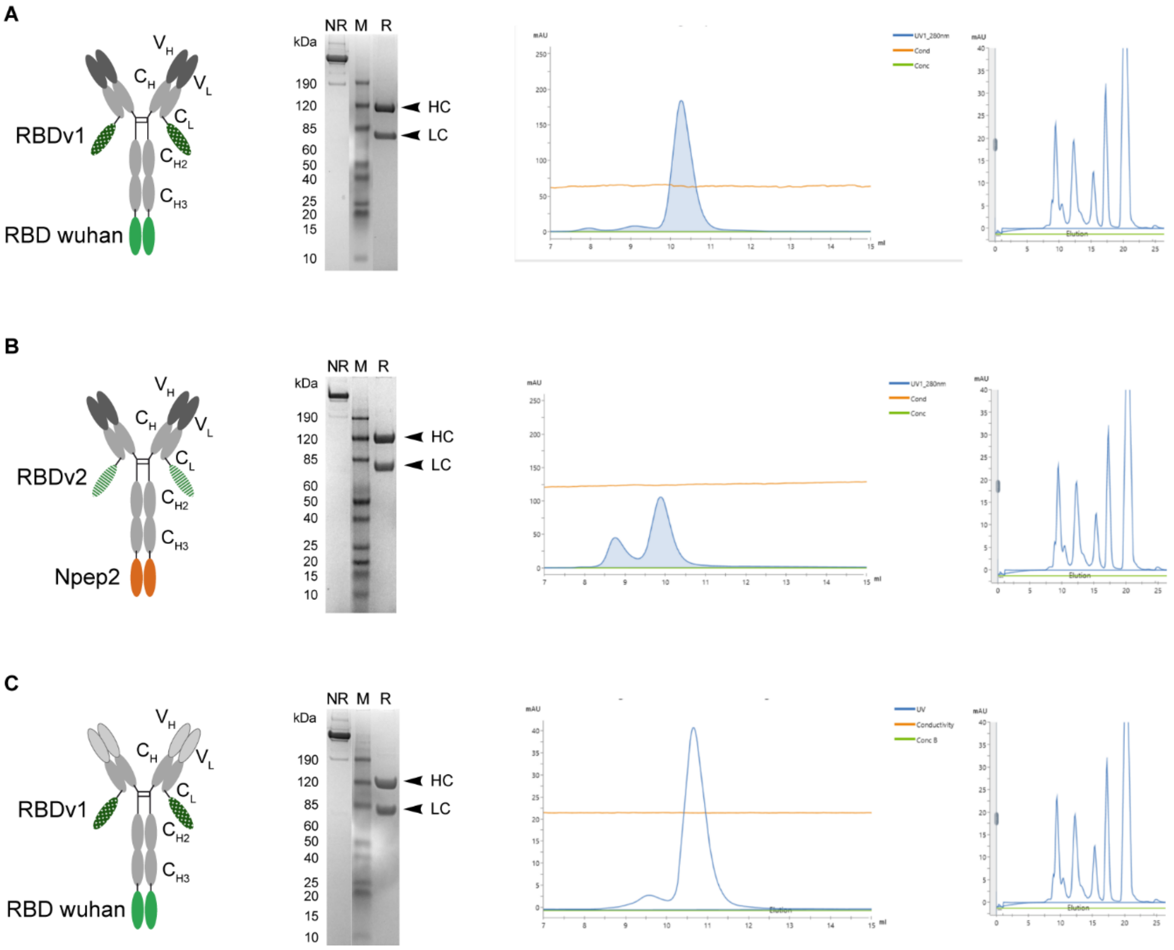

Figure S3. Structure and quality control of the CD40.RBDv, IgG4.RBDv and CD40.Pan.CoV vaccines. From left to right are shown panels for: Schematic representation of the vaccine, SDS-PAGE profiles under non-reduced (NR) and reduced (R) conditions, and Size exclusion chromatography (SEC, Sephadex® 200 10/300 GL prepacked gel filtration column (GE Healthcare)) of CD40.RBDv (**A**), CD40.Pan.CoV (**B**) and IgG4.RBDv (**C**). For SDS-PAGE, molecular weight (kDa) markers are shown on the left. Arrows indicate the heavy chain (HC) and light chain (LC) of the vaccine. Predicted sizes are based on the amino acid composition. HC and LC bands presented are as predicted, allowing for additional mass from expected glycosylations. For the SEC, standard protein molecular weight marker traces are shown on the right panel.

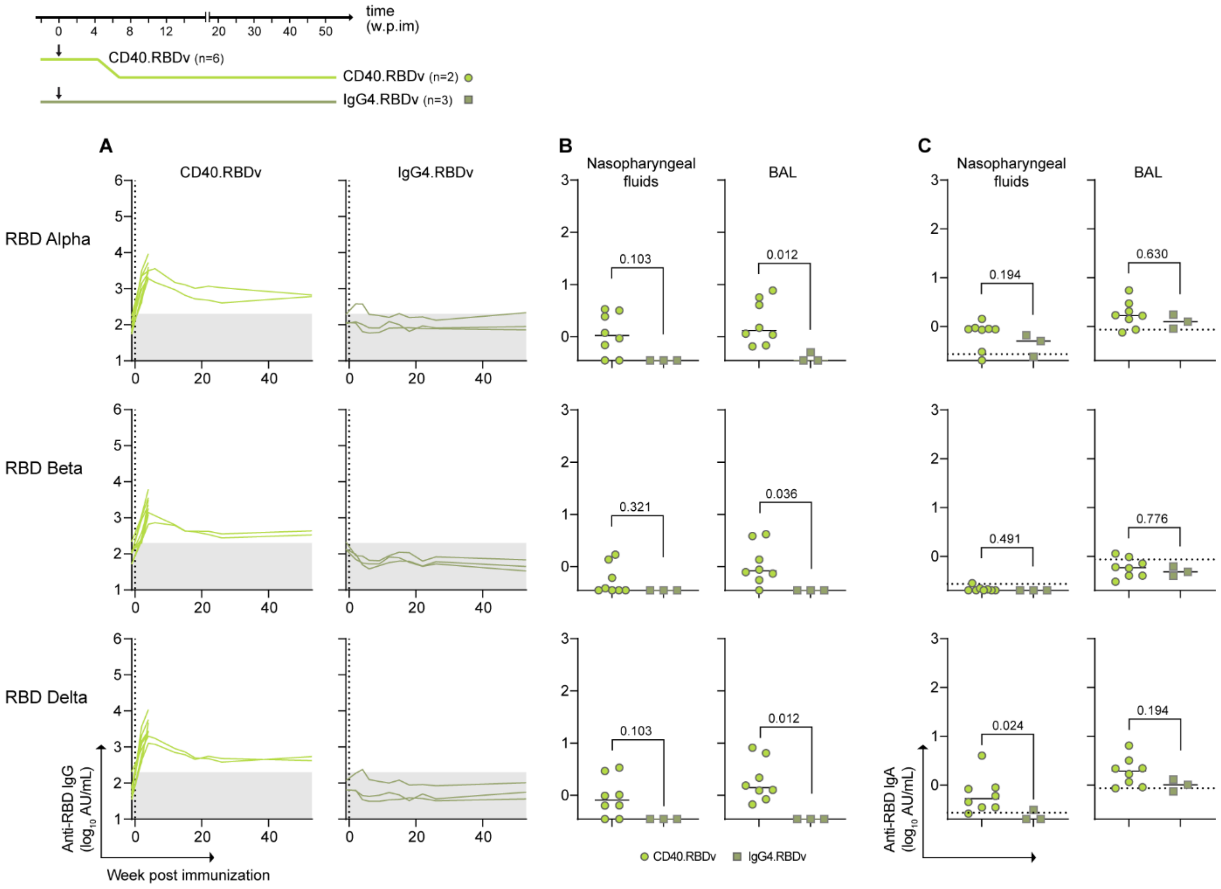

Figure S4. Titers of anti-SARS-CoV-2 antibodies measured in naive NHPs. (A & B) Specific Ab against RBD from Alpha, Beta and Delta VOCs. Individual serum measures (**A**) are indicated for CD40.RBDv (n=8, light green) and IgG4.RBDv (n=3, dark green) vaccinated animals. Dotted vertical line represents vaccine administrations and grey band indicates positivity cutoff (200 AU/ml). (**B & C**) Three weeks after vaccine injection, IgG (**B**) and IgA (**C**) titers against the RBD variants in nasopharyngeal fluids and BAL were compared between groups using the two-tailed non-parametric Mann-Whitney test. Dotted horizontal line indicates the median of non-immunized control animals. Horizontal lines indicate the median of each group.

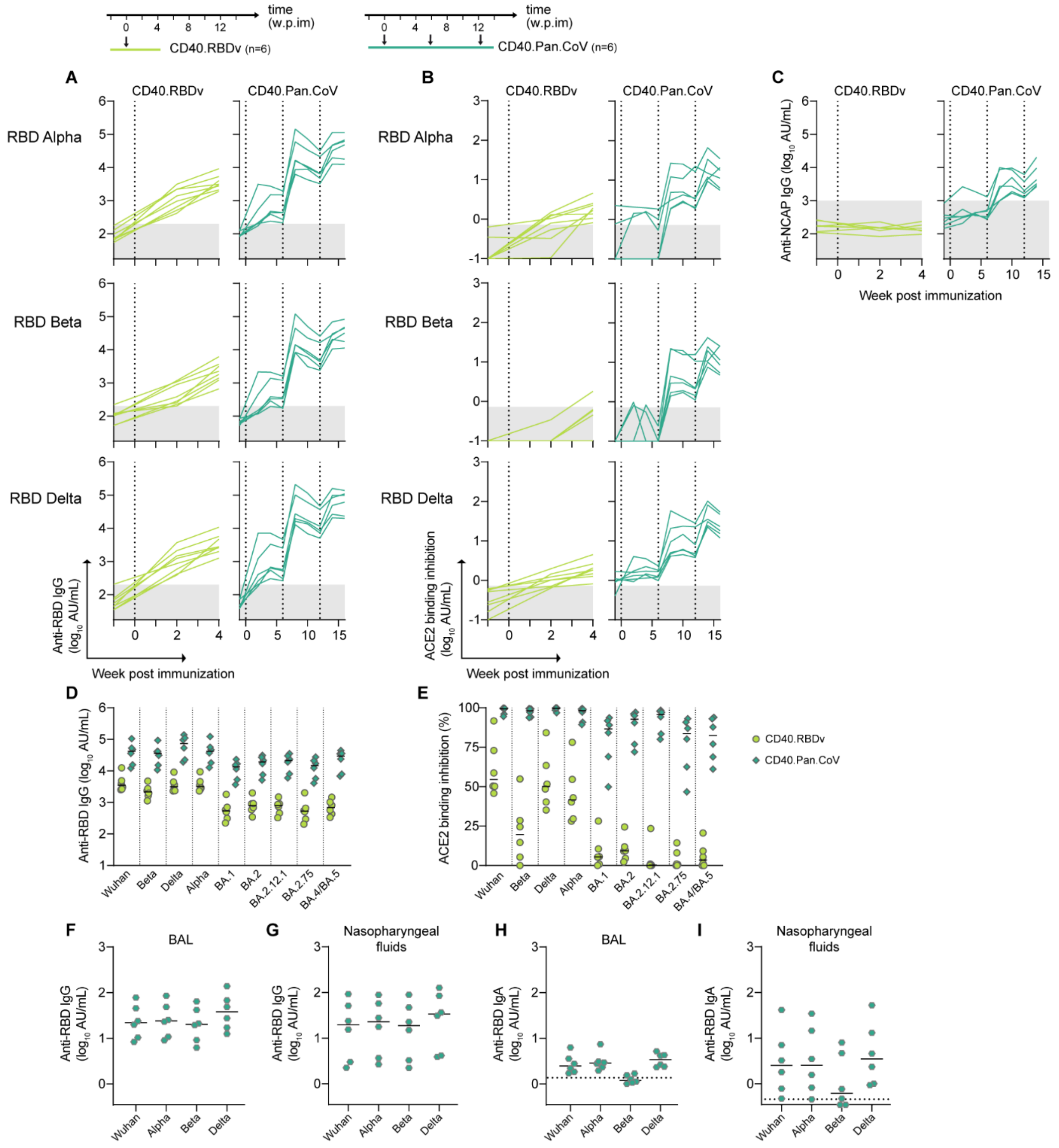

Figure S5. Ab response against VOC RBD sequences in naive NHPs. Individual values of IgG binding to different VOC RBD sequences (**A**), inhibition of binding to human ACE2 (**B**) and IgG binding to NCAP (**C**) were analysed in serum samples of naive animals. (**A-C**) Individual values are indicated for CD40.RBDv vaccinated animals (n=8, light green) and CD40.Pan.CoV vaccinated animal (n=6, dark cyan). Dotted vertical line represents vaccine administrations and grey band indicates positivity cutoff. Individual values of the Ab titer in serum (**D**) or the percentage of inhibition of ACE2 binding by animal serum (**E**) against different VOC RBD at 4 weeks post last vaccine injection (i.e., following a single injection of CD40.RBDv or 3 injections of CD40.Pan.CoV). Horizontal line indicates the median. Three weeks after the last vaccine injection, IgG (**F & G**) and IgA (**H & I**) titers against the RBD variants in BAL (**F & H**) and nasopharyngeal fluids (**G & I**) were measured in CD40.Pan.CoV vaccinated animal (n=6, dark cyan). Dotted horizontal line indicates the median of non-immunized control animals.

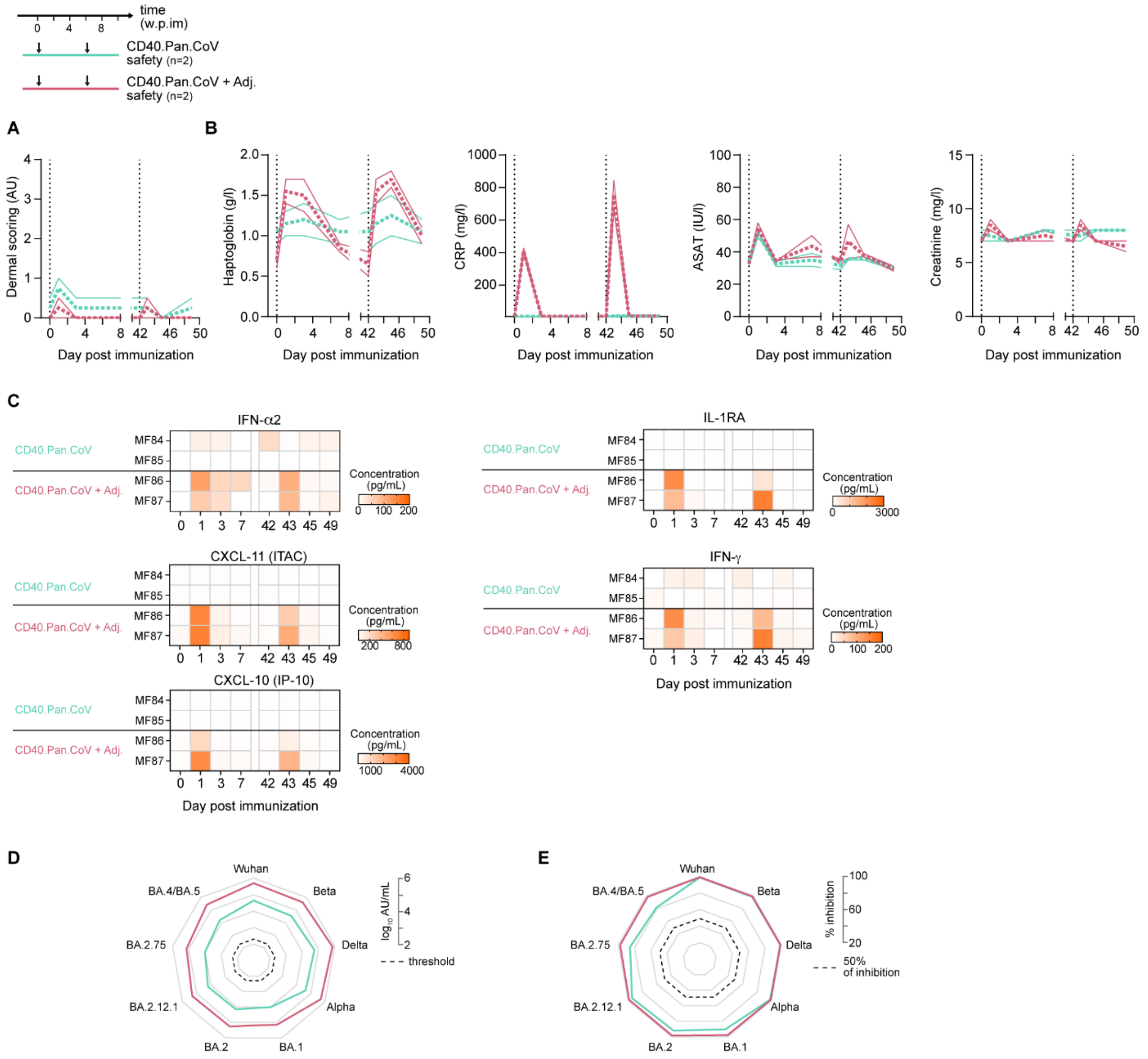

Figure S6. Safety data measured in CD40.Pan.CoV vaccinated animals. Immunoreaction and inflammation were analysed in CD40.Pan.CoV naive (n=2, turquoise) and CD40.Pan.CoV adjuvanted naive animals (n=2, Indian red). (**A**) Dermal score based on edema and erythema grade was analysed at the immunization area after each immunization. (**B**) Haptoglobin, C reactive protein, ASAT and creatinine were analysed in the plasma of vaccinated animals. Individual values (thin lines) and median (thick dotted lines) were indicated for each group. (**C**) Heatmaps of the plasma concentrations of IFN-α2, CXCL-11, CXCL-10, IL-1RA and IFN-γ. Each column represents one cytokine or chemokine; the colour scale (in pg/ml) is shown at the bottom. (**D & E**) Radar plots represent Ab titers in serum (**D**) or the percentage of inhibition of ACE2 binding by animal serum (**E**) against different VOC RBD at 4 weeks post last vaccine injection. Coloured line represents the median and dotted line indicates positivity cutoff (**D**) or 50% of inhibition (**E**).

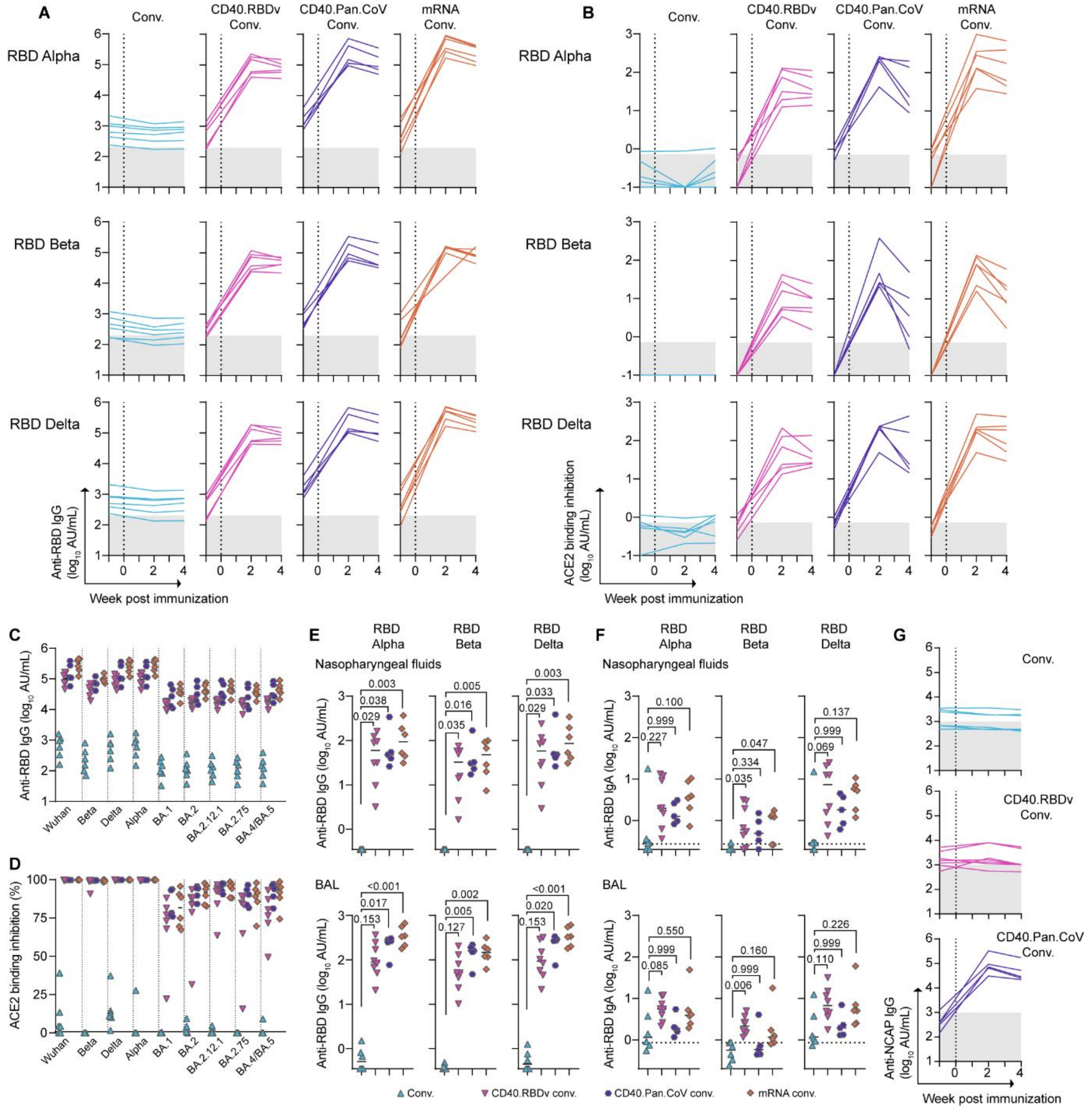

Figure S7. Titers of anti-SARS-CoV-2 antibodies measured in convalescent NHPs. Individual values of IgG and IgA binding to different VOC RBD sequences (**A, C, E & F**), inhibition of binding to human ACE2 (**B & D**) and IgG binding to NCAP (**G**) were analysed in serum samples of SARS-CoV-2 convalescent animals. Individual values are indicated for non-vaccinated (n=6, light blue); CD40.RBDv (n=6, pink), CD40.Pan.CoV (n=5, purple) and mRNA vaccinated convalescent animals (n=6, orange). Dotted vertical line represents vaccine administrations and grey band indicates positivity cutoff. Individual values of the Ab titer in serum (**C**) or the percentage of inhibition of ACE2 binding by animal serum (**D**) against different VOC RBD at 4 weeks post vaccine injection. Three weeks after vaccine injection, IgG (**E**) and IgA (**F**) titers against the RBD variants in nasopharyngeal fluids (top) and BAL (bottom) were compared between groups using using Kruskal-Wallis test following Dunn's multiple comparisons. Dotted horizontal line indicates the median of non-immunized control animals. Horizontal lines indicate the median of each group.

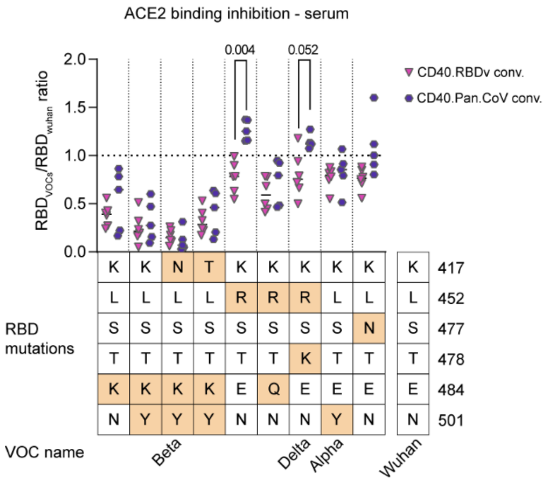

Figure S8. Comparison of antibody breadth responses measured in vaccinated convalescent NHPs. At 4 weeks post vaccination, inhibition of VOC RBD binding to human ACE2 measured in CD40.RBDv (n=6, pink) and CD40.Pan.CoV (n=5, purple) vaccinated convalescent animals, were compared with RBD Wuhan IgG titers as a reference (RBD_{VOCs} binding inhibition/ RBD_{Wuhan} binding inhibition). Amino acids at position 417, 452, 477, 478, 484 and 501 of the RBD sequences are indicated at the bottom. Mutations that differ from the original RBD Wuhan sequence are indicated in orange. RBD ratio were compared between groups using the two-tailed non-parametric Mann-Whitney test and significant p values are indicated.

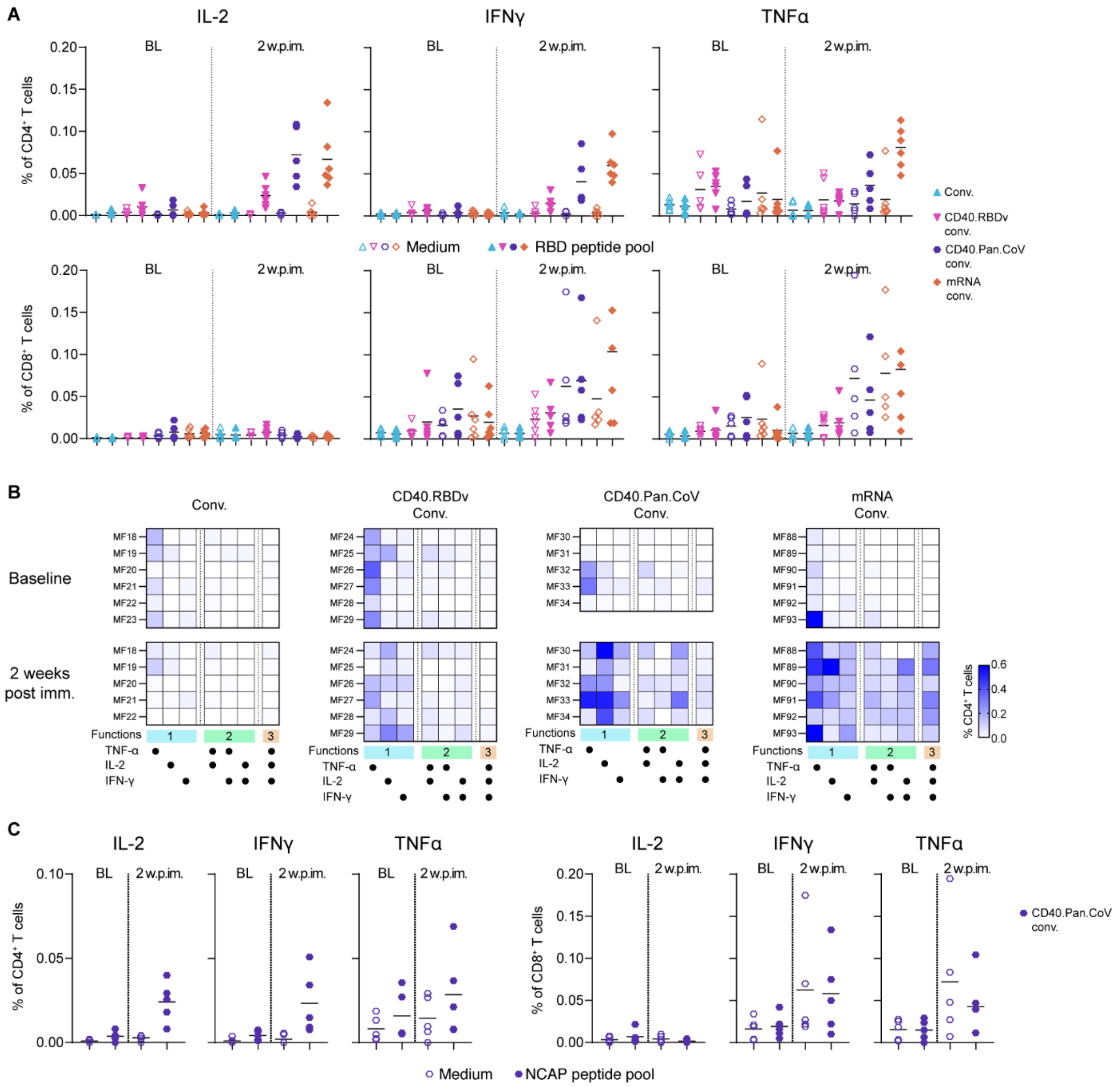

Figure S9. RBD-specific T-cell responses in convalescent NHPs. (**A**) Frequency of IL-2⁺ (left), IFNγ⁺ (middle) or TNFα⁺ (right) antigen-specific CD4⁺ T cells (CD154⁺) and CD8⁺ T cells (CD137⁺) in the total CD4⁺ T cell (top) or CD8⁺ T cell (bottom) populations, respectively, for each non-vaccinated (n=6, light blue), CD40.RBDv (n=6, pink), CD40.Pan.CoV (n=5, purple) and mRNA (n=6, orange) vaccinated convalescent animals. PBMCs were stimulated overnight with medium (open symbols) or SARS-CoV-2 RBD Wuhan overlapping peptide pool (filled symbols). BL: Baseline approximately 1 week before immunization. Bars indicate the mean values for each stimulation. (**B**) CD4⁺ T cell polyfunctionality analysed by Boolean gating is represented as a heat map for non-vaccinated, CD40.RBDv, CD40.Pan.CoV and mRNA vaccinated convalescent animals. Each line indicates one animal. (**C**) Frequency of antigen-specific CD4⁺ T cells (CD154⁺) and CD8⁺ T cells (CD137⁺) in the total CD4⁺ T cell (top) or CD8⁺ T cell (bottom) populations, respectively, against NCAP overlapping peptide pool in CD40.Pan.CoV vaccinated convalescent animals (n=5, purple).

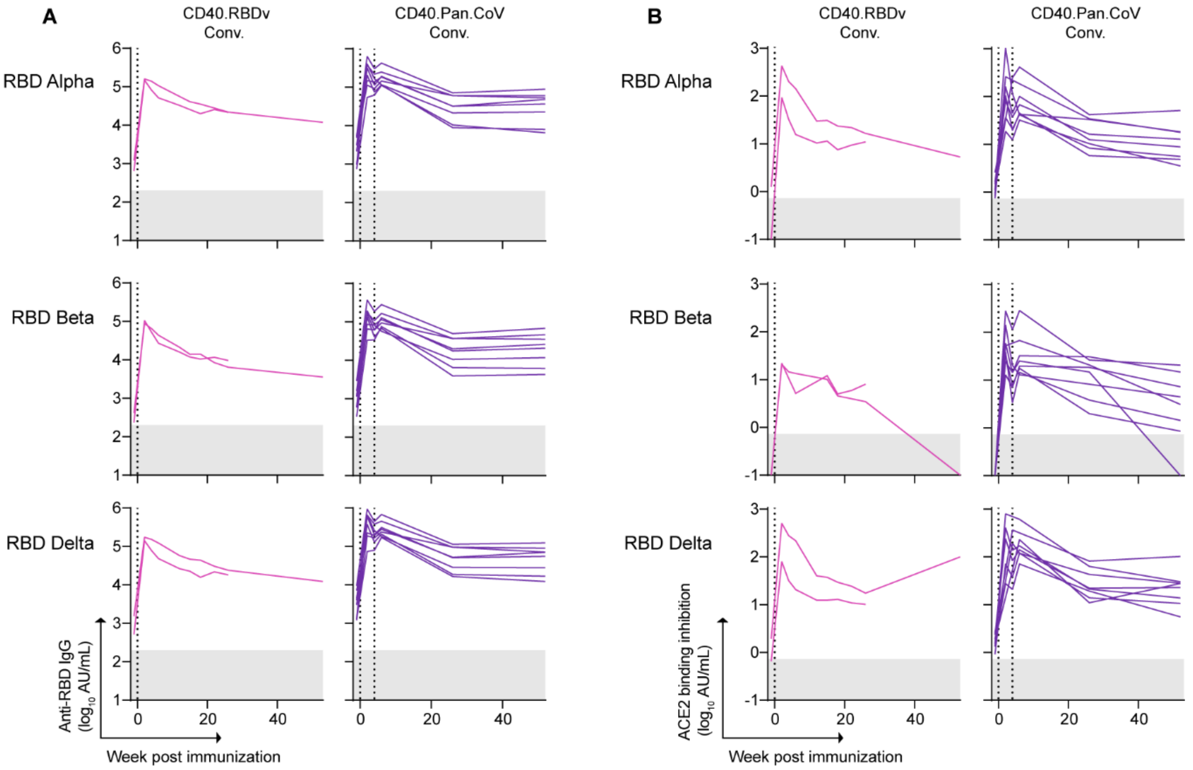

Figure S10. One-year follow-up of anti-SARS-CoV-2 antibodies in Wuhan SARS-CoV-2 convalescent NHPs. Individual values of IgG binding to different VOC RBD sequences (**A**) and inhibition of binding to human ACE2 (**B**) were analysed in serum samples of SARS-CoV-2 convalescent animals up to 53 weeks after immunization. Individual values are indicated for CD40.RBDv (n=2; pink) and CD40.Pan.CoV (n=8, purple) vaccinated convalescent animals. Dotted vertical line represents vaccine administrations and grey band indicates positivity cutoff.

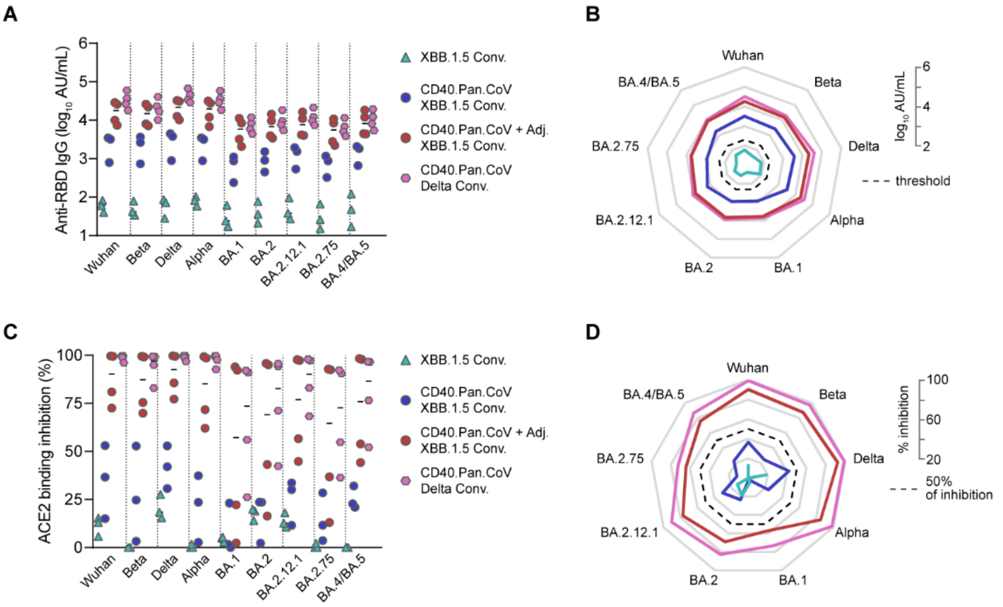

Figure S11. Durability of anti-SARS-CoV-2 antibodies in Omicron XBB.1.5 or Delta SARS-CoV-2 convalescent NHPs. Individual values of IgG binding to Wuhan RBD (**A**) and inhibition of binding to human ACE2 (**C**) were analysed in serum samples of SARS-CoV-2 convalescent animals at 42 weeks after immunization. Individual values are indicated for non-vaccinated Omicron XBB.1.5 convalescent (n=3, light sea green), CD40.Pan.CoV vaccinated Omicron XBB.1.5 convalescent (n=4, dark blue), adjuvanted CD40.Pan.CoV vaccinated Omicron XBB.1.5 convalescent (n=4, firebrick) and CD40.Pan.CoV vaccinated Delta convalescent animals (n=4, light pink). Radar plots represent Ab titers in serum (**B**) or the percentage of inhibition of ACE2 binding by animal serum (**D**) against different VOC RBD at 42 weeks post vaccine injection. Coloured line represents the median group and dotted line indicates positivity cutoff **(B)** or 50% of inhibition (**D**).

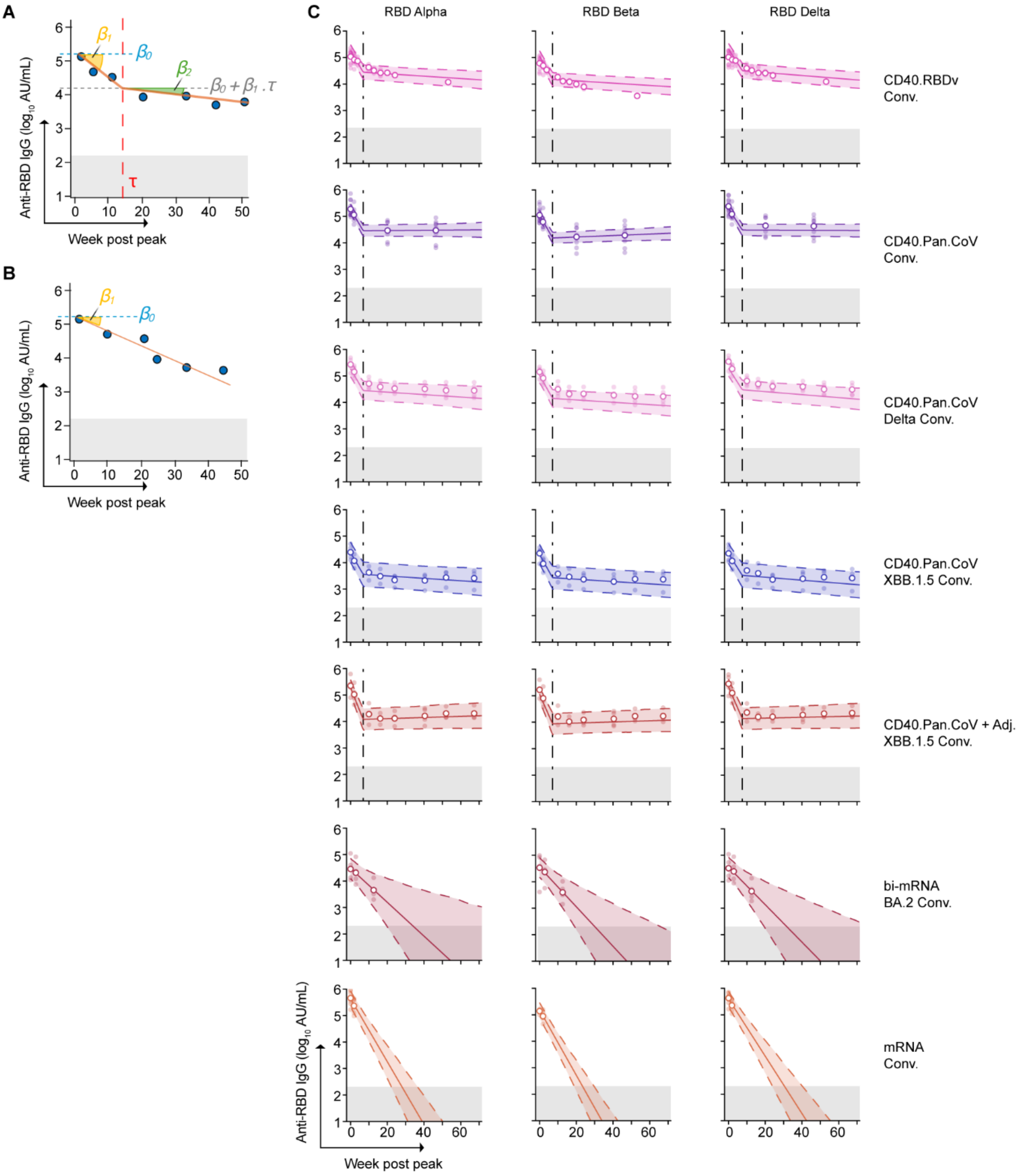

Figure S12. Modeling of the durability of anti-SARS-CoV-2 antibodies binding to Alpha, Beta and Delta RBD in SARS-CoV-2 convalescent vaccinated NHPs. (A) Schematic of the linear model with breakpoint used to describe the biphasic dynamics of antibody responses observed post-peak with β_0, β_1 and β_2 the intercept, the first and the second slope, respectively, and τ the breakpoint. **(B)** Schematic of the linear mixed model used to describe the monophasic dynamics of antibody responses observed post-peak with β_0 and β_1 the intercept and the slope, respectively. **(C)** Post-peak IgG binding to Alpha (1st column), Beta (2nd column) and Delta (3rd column) RBD predicted by the pooled monophasic-biphasic linear mixed model. Median (large white dot) and individual value (small coloured circles) are indicated for CD40.RBDv vaccinated Wuhan SARS-CoV-2 convalescent (n=8, dark pink), CD40.Pan.CoV vaccinated Wuhan SARS-CoV-2 convalescents (n=13, purple), CD40.Pan.CoV Vaccinated Delta SARS-CoV-2 convalescent (n=4, light pink), CD40.Pan.CoV vaccinated XBB.1.5 SARS-CoV-2 convalescent (n=4, dark blue), adjuvanted CD40.Pan.CoV vaccinated XBB.1.5 SARS-CoV-2 convalescent animals (n=4, firebrick), bivalent mRNA vaccinated BA.2 SARS-CoV-2 convalescent (n=4, dark red), and mRNA vaccinated Wuhan SARS-CoV-2 convalescent (n=6, orange). Median prediction and its 95% prediction interval are represented by the solid thick line and coloured shaded area, respectively. The black vertical dash-dotted line indicates the breakpoint time τ estimated in the model for groups described by the biphasic model, and the gray band indicates positivity cutoff.

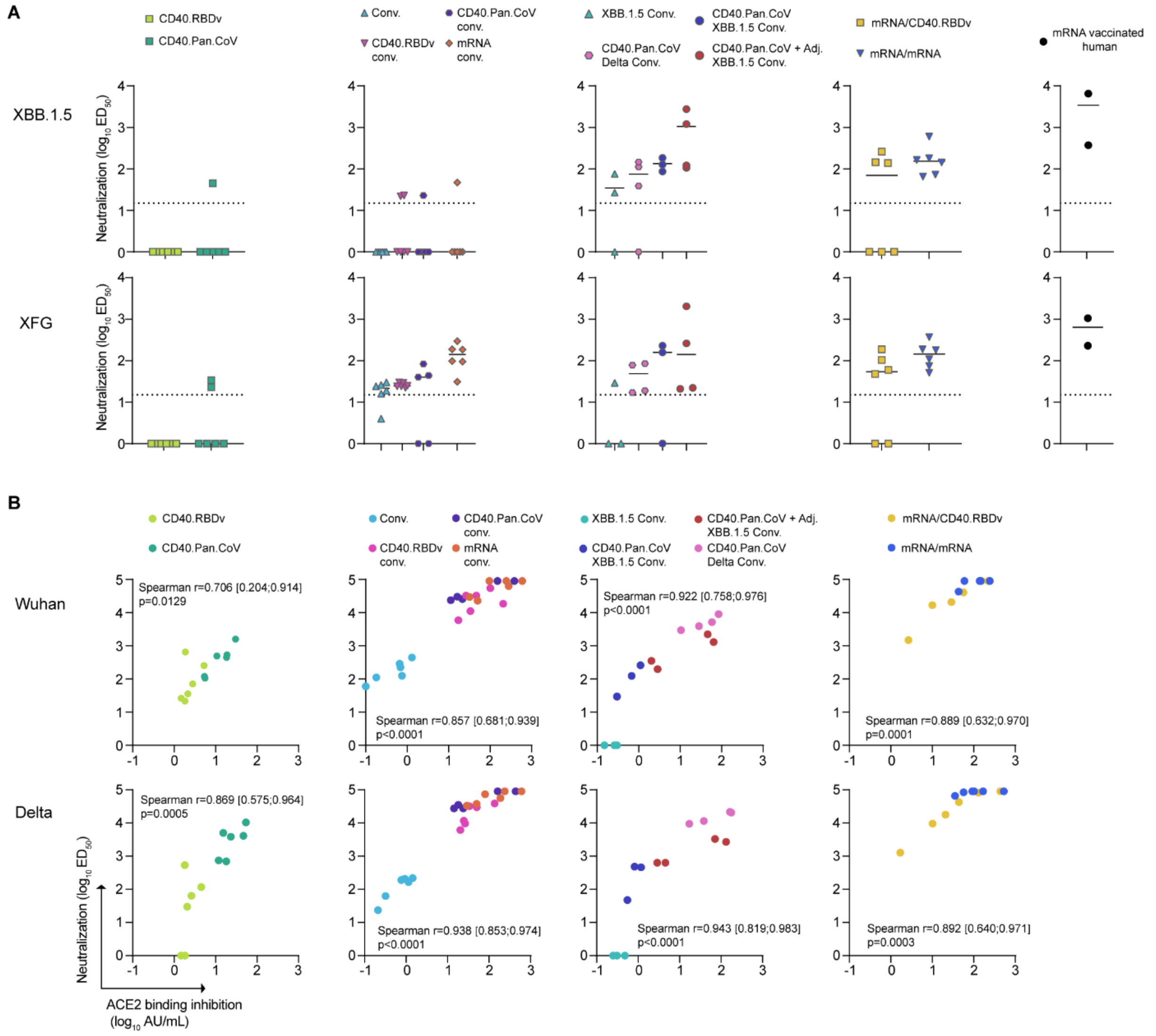

Figure S13. Neutralization responses in naive and SARS-CoV-2 convalescent NHPs. Neutralization against SARS-CoV-2 strains and inhibition of binding to human ACE2 were analysed in serum samples of CD40.RBDv naive (n=8, light green), CD40.Pan.CoV naive (n=6, dark cyan), SARS-CoV-2 convalescent (n=6, light blue), CD40.RBDv convalescent (n=6, pink), CD40.Pan.CoV convalescent (n=5, purple), mRNA convalescent (n=6, orange), mRNA/CD40.RBDv vaccinated (n=6, yellow) and mRNA/mRNA vaccinated (n=6, blue) convalescent animals at 4 weeks after the last immunization; and in serum samples of non-vaccinated Omicron XBB.1.5 convalescent (n=3, light sea green), CD40.Pan.CoV vaccinated Omicron XBB.1.5 convalescent (n=4, dark blue), adjuvanted CD40.Pan.CoV vaccinated Omicron XBB.1.5 convalescent (n=4, firebrick) and CD40.Pan.CoV vaccinated Delta convalescent animals (n=4, light pink) at 42 weeks after immunization. (**A**) Neutralization titers against Omicron XBB.1.5 and XFG subvariants measured in animal sera and sera from XBB.1.5 mRNA vaccinated humans (black). Horizontal dotted line indicates positivity cutoff. (**B**) Correlation between inhibition of binding to human ACE2 and neutralization for Wuhan (top) and Delta (bottom) virus in naive, SARS-CoV-2 convalescent or mRNA primed animals. The spearman r [95% CI] and p (two-tailed) values are indicated.

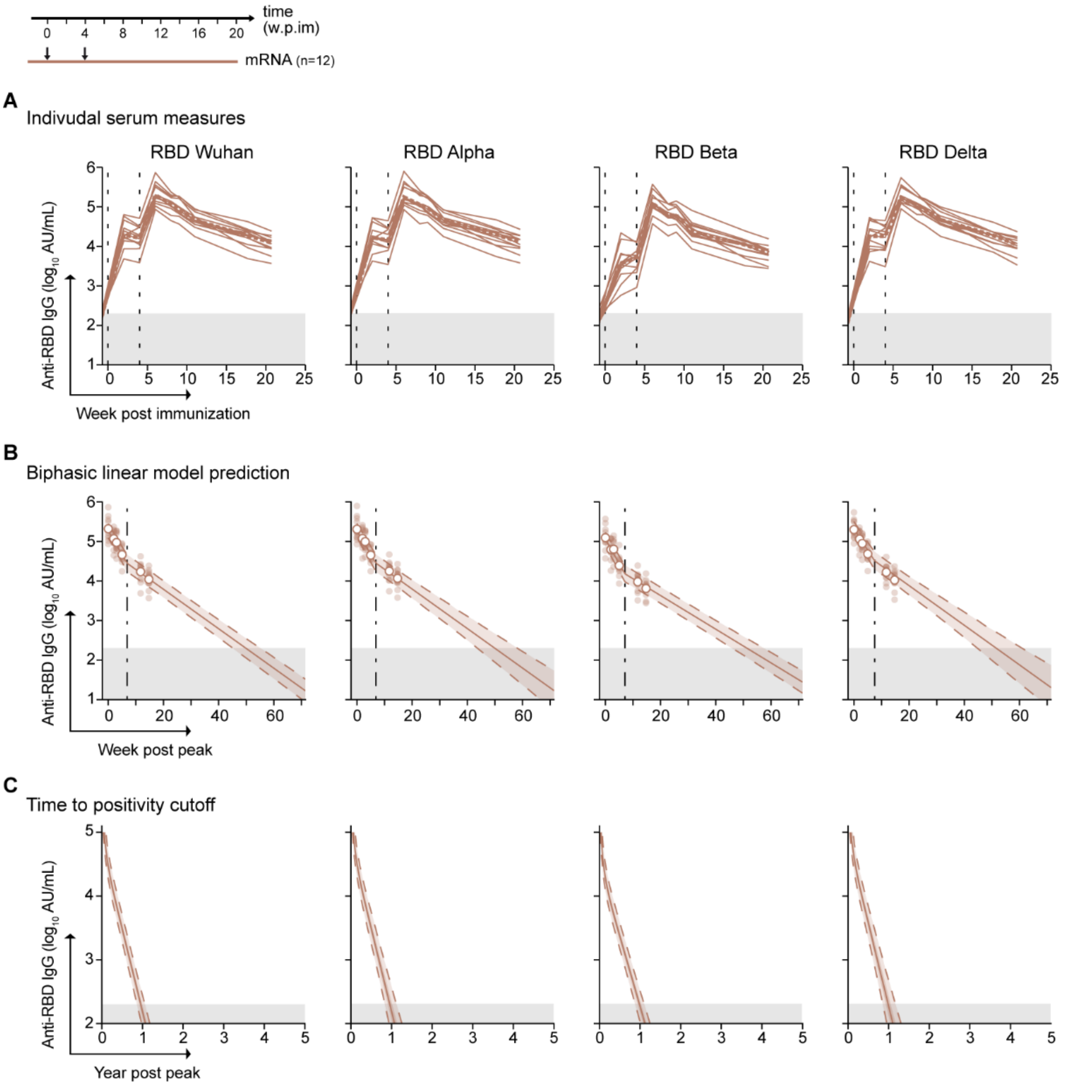

Figure S14. Analysis of the durability of anti-SARS-CoV-2 antibodies binding to Wuhan, Alpha, Beta and Delta RBD in mRNA vaccinated NHPs. (**A**) IgG binding to RBD were analysed in serum samples of mRNA vaccinated animals. Individual values (thin lines) and median (thick dotted lines) were indicated for mRNA vaccinated animals (n=12, brown). Dotted vertical line represents mRNA vaccine administrations. (**A-C**) Gray band indicates positivity cutoff. (**B**) Post-peak IgG binding to RBD predicted by the pooled monophasic-biphasic linear mixed model. Median (thick solid line) and its 95% confidence interval (coloured shaded area) predicted by the model are compared to observations with median and individual observed dynamics represented by large white dots and small circles, respectively. The black vertical dash-dotted line indicates the breakpoint time τ estimated in the model. (**C**) Relationship between IgG binding to Wuhan RBD levels and time post peak, in years, required to reach them predicted by the model. Median prediction is indicated by the solid line, and 95% and 50% prediction intervals are displayed by light and dark shaded areas, respectively.

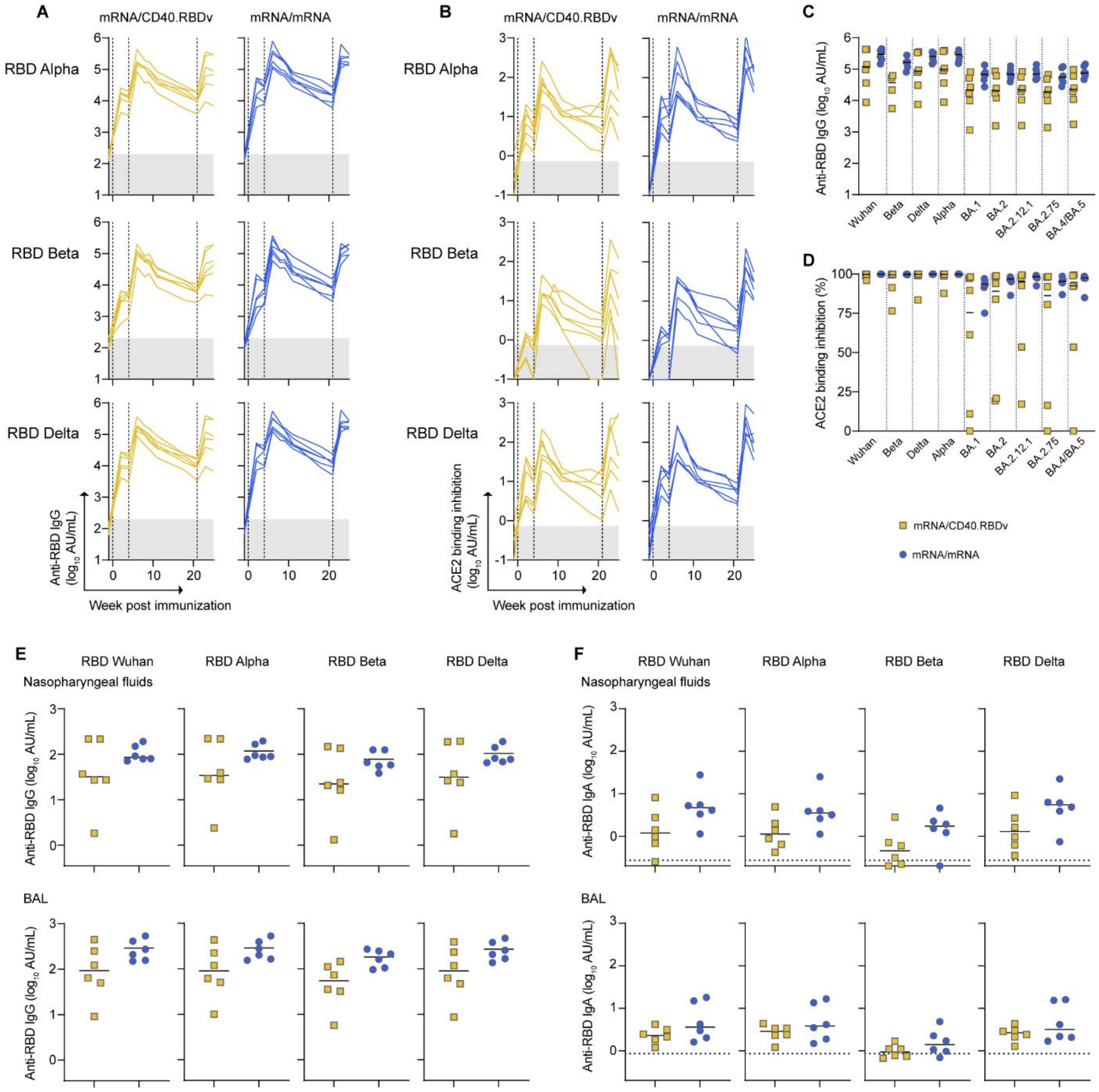

Figure S15. Titers of anti-SARS-CoV-2 antibodies measured in serum of mRNA primed NHPs. Individual values of IgG binding to different VOC RBD sequences (**A**) and inhibition of binding to human ACE2 (**B**) were analysed in serum samples of mRNA primed animals. Individual values are indicated for mRNA/CD40.RBDv (n=6, yellow) and mRNA/mRNA (n=6, blue) groups. Dotted vertical line represents vaccine administrations and grey band indicates positivity cutoff. Individual values of the Ab titer in serum (**C**) or the percentage of inhibition of ACE2 binding by animal serum (**D**) against different VOC RBD at 4 weeks post last vaccine injection. Horizontal line indicates the median. Three weeks after the last vaccine injection, IgG (**E**) and IgA (**F**) titers against the RBD variants in nasopharyngeal fluids (top) and BAL (bottom) were measured in mRNA/CD40.RBDv (n=6, yellow) and mRNA/mRNA (n=6, blue) vaccinated animals. Dotted horizontal line indicates the median of non-immunized control animals. Horizontal lines indicate the median of each group.

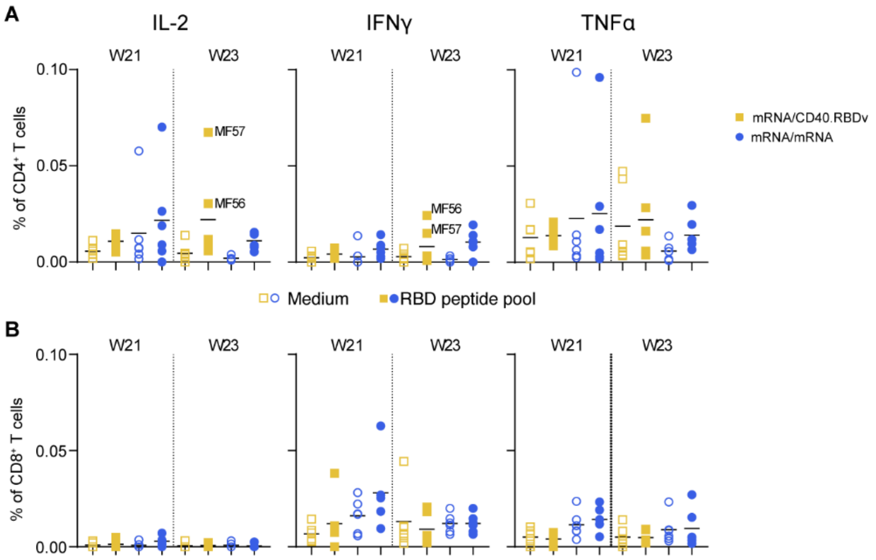

Figure S16. RBD-specific T-cell responses in mRNA primed NHPs. Frequency of IL-2⁺ (left), IFNγ⁺ (middle) or TFNα⁺ (right) antigen-specific CD4⁺ T cells (CD154⁺) and CD8⁺ T cells (CD137⁺) in the total CD4⁺ T cell (**A**) or CD8⁺ T cell (**B**) population, respectively, for each mRNA/CD40.RBDv (n=6, yellow) and mRNA/mRNA (n=6, blue) vaccinated animals. PBMCs were stimulated overnight with medium (open symbols) or SARS-CoV-2 RBD Wuhan overlapping peptide pool (filled symbols). W21: week 21 post immunization, i.e., the baseline of CD40.RBDv or mRNA boost; W23: Two weeks post CD40.RBDv or third mRNA immunization. Bars indicate the mean values for each stimulation.

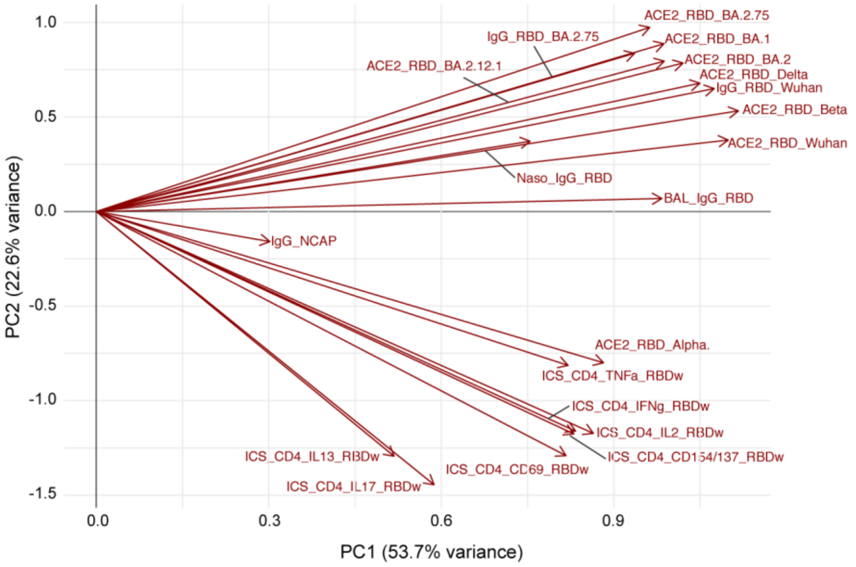

Figure S17. Variable contributions within PCA conducted on experimental groups and immunogenicity measures. The arrows represent the contribution of each variable to the first two principal components. The length and direction of arrow indicate the influence and association of variable to the principal components. Names of variables are indicated.

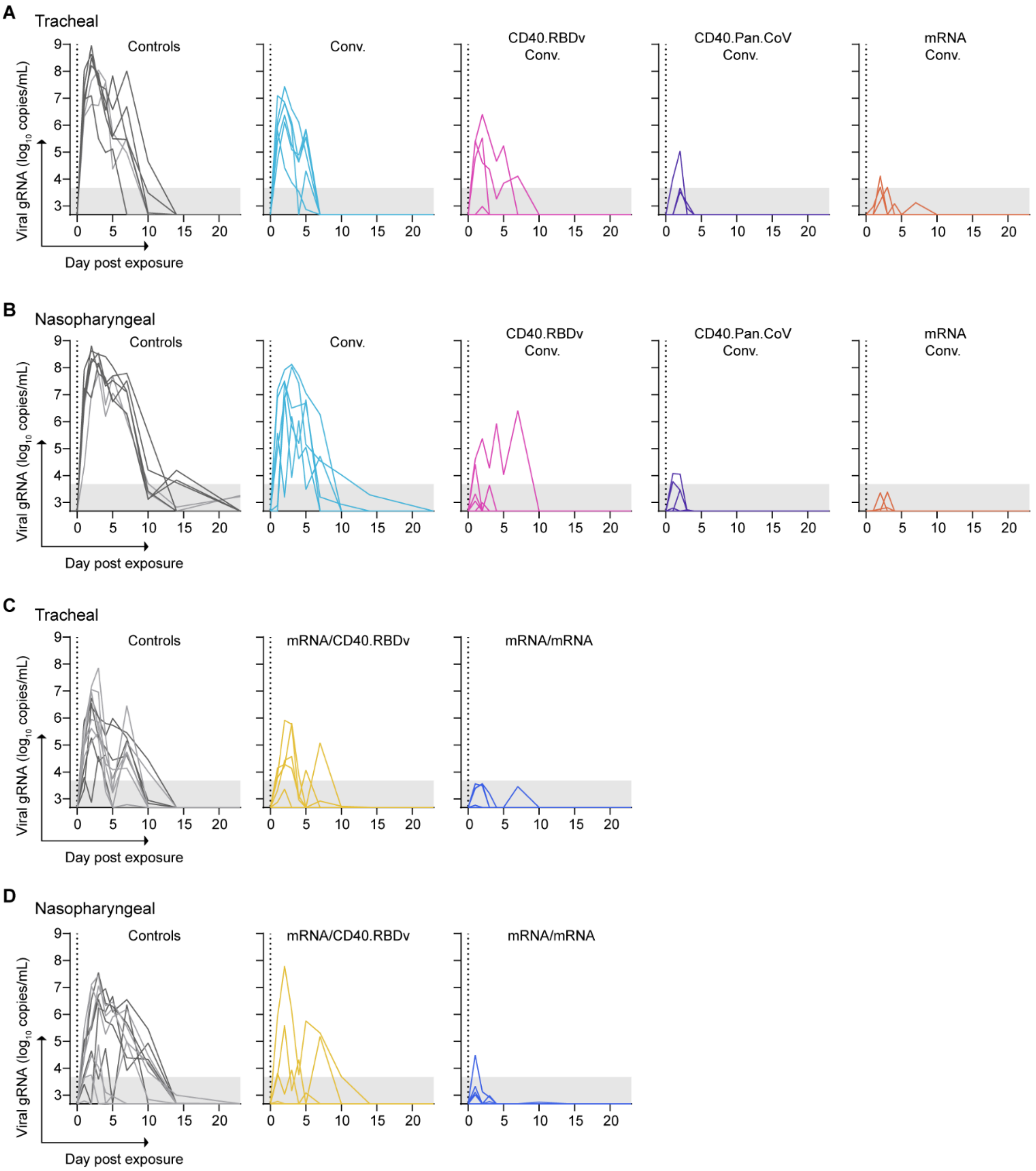

Figure S18. Individual SARS-CoV-2 viral loads after viral exposure. Viral loads were measured after Delta B.1.617.2 (**A & B**) and Omicron BA.1 (**C & D**) exposure. Tracheal (**A & C**) and nasopharyngeal (**B & D**) viral loads determined by RT-qPCR in animal of control (n=7 and n=11, grey), convalescent (n=6, light blue), CD40.RBDv convalescent (n=6, pink), CD40.Pan.CoV convalescent (n=5, purple), mRNA convalescent (n=6, orange), mRNA/CD40.RBDv vaccinated (n=6, yellow) and mRNA/mRNA vaccinated (n=6, blue) groups. Dotted vertical line represents viral exposure and grey band indicates limit of quantification.

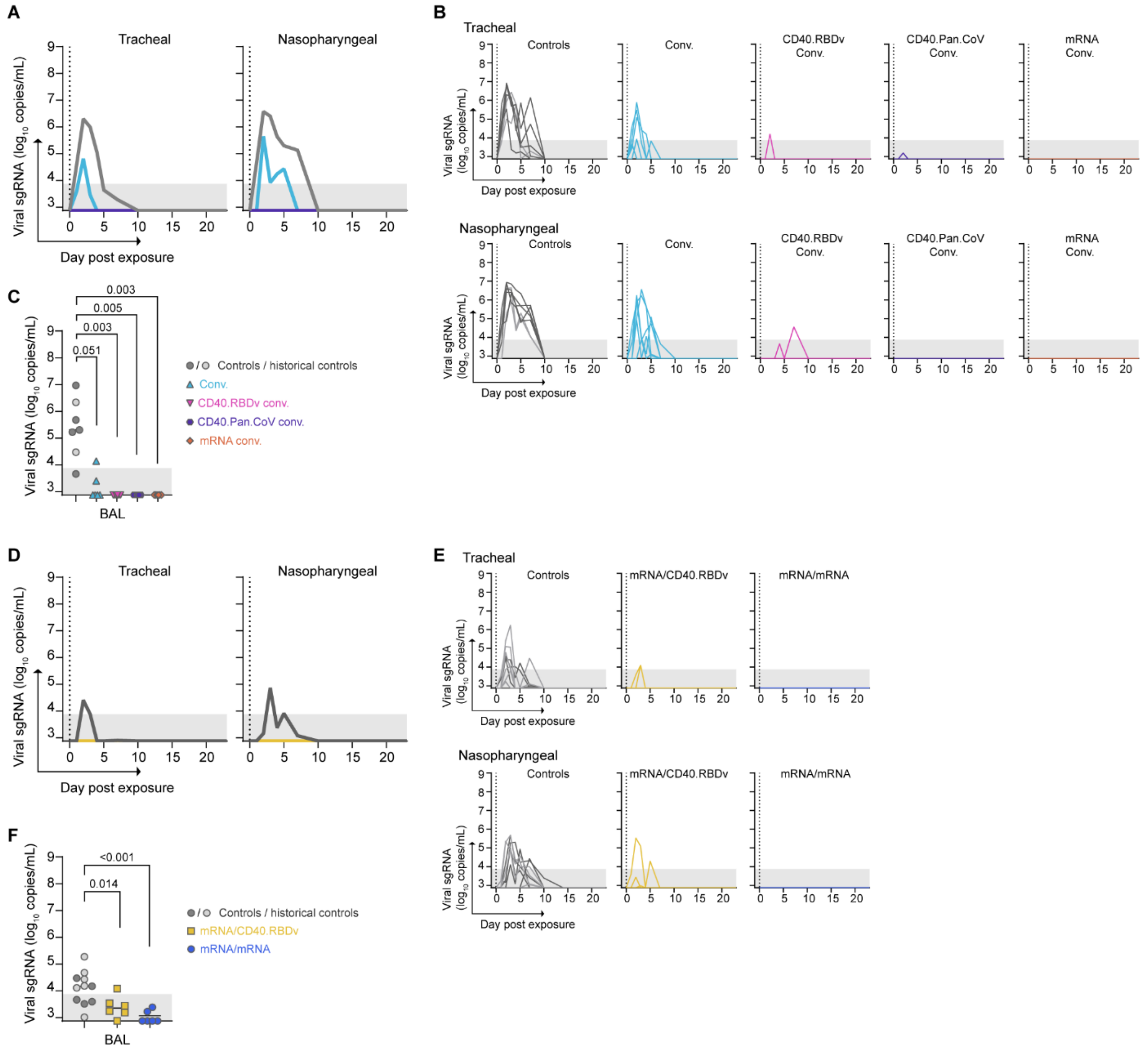

Figure S19. Detection of SARS-CoV-2 sub-genomic RNA after viral exposure. Sub-genomic RNA (sgRNA) was measured after Delta B.1.617.2 (**A-C**) and Omicron BA.1 (**d-f**) exposure. Tracheal and nasopharyngeal viral loads determined by RT-qPCR in animal of control (n=7 and n=11, grey), convalescent (n=6, light blue), CD40.RBDv convalescent (n=6, pink), CD40.Pan.CoV convalescent (n=5, purple), mRNA convalescent (n=6, orange), and mRNA/CD40.RBDv vaccinated (n=6, yellow) and mRNA/mRNA (n=6, blue) groups. Lines indicate the median (**A & D**) or individual value (**B & E**), dotted vertical line represents viral exposure and grey band indicates limit of quantification. (**C & F**) Quantification of sgRNA in BAL at 3 days post exposure. Symbol represents individual value and median value is indicated by horizontal bar. Grey band indicates limit of quantification. Viral loads were compared between Delta B.1.617.2 exposed groups using Kruskal-Wallis test following Dunn's multiple comparisons (**C**), and Omicron BA.1 exposed groups using the two-tailed non-parametric Mann-Whitney test (**F**).

Supplementary tables

Supplementary table I. Names and sequences of primers.

**Name**	**Sequences (5'-3')**
RdRp gene / nCoV_IP4	
nCoV_IP4-14059Fw	GGTAACTGGTATGATTTCG
nCoV_IP4-14146Rv	CTGGTCAAGGTTAATATAGG
nCoV_IP4-14084Probe(+)	TCATACAAACCACGCCAGG [5']Fam [3']BHQ-1
SARS-CoV-2 E gene sub-genomic mRNA	
leader-specific primer sgLeadSARSCoV2-F	CGATCTCTTGTAGATCTGTTCTC
E-Sarbeco-R primer	ATATTGCAGCAGTACGCACACA
E-Sarbeco probe	ACACTAGCCATCCTTACTGCGCTTCG [5']HEX [3']BHQ-1

Supplementary table II. Estimation of the three pooled monophasic-biphasic linear mixed models on anti-SARS-CoV-2 antibodies binding to Alpha, Beta and Delta RDB for the convalescent groups vaccinated either by the CD40 targeting vaccines or mRNA vaccines, and prediction of the time required to reach the positivity cutoff of 200 AU/mL.

	Breakpoint (τ)<br><br>(days post-peak)<br>Mean [95% CI]<br>P-value[c]	Peak Value $(\boldsymbol{\beta_0})$<br><br>($\log_{10}$ AU/mL)<br>Mean [95% CI]<br>P-value[c]	First Slope $(\boldsymbol{\beta_1})$<br><br>($\log_{10}$ AU/mL/Week)<br>Mean [95% CI]<br>P-value[c]	Second Slope $(\boldsymbol{\beta_2})$<br><br>($\log_{10}$ AU/mL/Week)<br>Mean [95% CI]<br>P-value[c]	Time to the positivity cutoff [a]<br>(years post-peak)<br>Median [Q1; Q3]<br>Cutoff reached (%)[d]
Model on IgG binding to Alpha RBD					
Groups described by the biphasic part of the model					
CD40.RBDv Conv.<br>[reference]		5.27<br>[5.18 ; 5.36]	-0.119<br>[-0.130 ; -0.108]	-0.005<br>[-0.007 ; -0.002]	12.26<br>[9.15 ; 17.39]<br>(93.1%)
CD40.Pan.CoV Conv.		5.27<br>[5.18 ; 5.36]<br>(na)	-0.119<br>[-0.130 ; -0.108]<br>(na)	0.001<br>[-0.002 ; 0.003]<br>(***)	40.28<br>[25.44 ; 75.15]<br>(42.2%)
CD40.Pan.CoV Delta Conv.	48.06<br>[46.88; 49.24]<br>(na)	5.27<br>[5.18 ; 5.36]<br>(na)	-0.119<br>[-0.130 ; -0.108]<br>(na)	-0.005<br>[-0.007 ; -0.002]<br>(na)	10.43<br>[7.82 ; 15.10]<br>(93.3%)
CD40.Pan.CoV XBB.1.5 Conv.		4.37<br>[4.09 ; 4.65]<br>(***)	-0.119<br>[-0.130 ; -0.108]<br>(na)	-0.005<br>[-0.007 ; -0.002]<br>(na)	6.27<br>[4.63 ; 9.48]<br>(93.4%)
CD40.Pan.CoV+Adj XBB.1.5 Conv.		5.27<br>[5.18 ; 5.36]<br>(na)	-0.174<br>[-0.205 ; -0.143]<br>(**)	0.002<br>[-0.001 ; 0.005]<br>(***)	26.57<br>[14.98 ; 59.98]<br>(24.0%)
Groups described by the monophasic part of the model					
Bi-mRNA BA.2 Conv.	[b]	4.47<br>[4.20 ; 4.75]<br>(***)	-0.064<br>[-0.094 ; -0.034]<br>(*)	[b]	*0.75* [b]<br>*[0.58 ; 1.11]*<br>*(97.2%)*
mRNA Conv.	[b]	5.63<br>[5.41 ; 5.86]<br>(*)	-0.119<br>[-0.130 ; -0.108]<br>(na)	[b]	*0.57* [b]<br>*[0.52 ; 0.63]*<br>*(100%)*

Model on IgG binding to Beta RBD					
Groups described by the biphasic part of the model					
CD40.RBDv Conv.		5.06 [4.97 ; 5.14]	-0.123 [-0.133 ; -0.113]	-0.004 [-0.006 ; -0.003]	10.78 [8.48 ; 14.74] (95.9%)
CD40.Pan.CoV Conv.		5.06 [4.97 ; 5.14] (na)	-0.123 [-0.133 ; -0.113] (na)	0.003 [0.001 ; 0.005] (***)	51.03 [28.84 ; 102.88] (13.2%)
CD40.Pan.CoV Delta Conv.	49.55 [47.63; 51.47] (na)	5.06 [4.97 ; 5.14] (na)	-0.123 [-0.133 ; -0.113] (na)	-0.004 [-0.006 ; -0.003] (na)	9.66 [7.55 ; 13.45] (96.3%)
CD40.Pan.CoV XBB.1.5 Conv.		4.30 [4.03 ; 4.57] (***)	-0.123 [-0.133 ; -0.113] (na)	-0.004 [-0.006 ; -0.003] (na)	5.86 [4.39 ; 8.16] (95.7%)
CD40.Pan.CoV+Adj XBB.1.5 Conv.		5.06 [4.97 ; 5.14] (na)	-0.159 [-0.196 ; -0.122] (*)	0.002 [-0.002 ; 0.006] (**)	29.12 [15.47 ; 65.92] (24.0%)
Groups described by the monophasic part of the model					
Bi-mRNA BA.2 Conv.	[b]	4.55 [4.28 ; 4.81] (***)	-0.075 [-0.103 ; -0.047] (**)	[b]	*0.64* [b] *[0.52 ; 0.84] (98.9%)*
mRNA Conv.	[b]	5.18 [4.96 ; 5.40] (3e-1)	-0.123 [-0.133 ; -0.113] (na)	[b]	*0.47* [b] *[0.43 ; 0.51] (100%)*

Model on IgG binding to Delta RBD					
Groups described by the biphasic part of the model					
CD40.RBDv Conv.		5.32 [5.23 ; 5.41]	-0.108 [-0.119 ; -0.097]	-0.006 [-0.007 ; -0.004]	8.82 [7.40 ; 10.81] (99.4%)
CD40.Pan.CoV Conv.		5.32 [5.23 ; 5.41] (na)	-0.108 [-0.119 ; -0.097] (na)	0.000 [-0.002 ; 0.001] (***)	54.13 [34.91 ; 102.50] (55.2%)
CD40.Pan.CoV Delta Conv.	52.44 [50.63; 54.26] (na)	5.32 [5.23 ; 5.41] (na)	-0.108 [-0.119 ; -0.097] (na)	-0.006 [-0.007 ; -0.004] (na)	8.38 [6.96 ; 10.40] (99.6%)
CD40.Pan.CoV XBB.1.5 Conv.		4.33 [4.05 ; 4.60] (***)	-0.108 [-0.119 ; -0.097] (na)	-0.006 [-0.007 ; -0.004] (na)	4.76 [3.77 ; 6.10] (99.5%)
CD40.Pan.CoV+Adj XBB.1.5 Conv.		5.32 [5.23 ; 5.41] (na)	-0.159 [-0.188 ; -0.130] (**)	0.002 [-0.001 ; 0.004] (***)	40.42 [23.29 ; 96.15] (24.4%)
Groups described by the monophasic part of the model					
Bi-mRNA BA.2 Conv.	[b]	4.54 [4.27 ; 4.82] (***)	-0.071 [-0.100 ; -0.042] (*)	[b]	*0.69* [b] *[0.55 ; 0.94] (98.3%)*
mRNA Conv.	[b]	5.60 [5.37 ; 5.83] (*)	-0.108 [-0.119 ; -0.097] (na)	[b]	*0.63* [b] *[0.57 ; 0.70] (100%)*

[a] Threshold of 200 AU/mL. The median, Q1 and Q3 were extracted as the 50th, 25th, and 75th percentiles of the distribution of the time to positivity cutoff calculated on parameters simulated from the empirical posterior distribution. The reader can refer to the Supplementary Materials for more information [b] The available data do not allow estimation of a second slope because the follow-up duration is insufficient, strongly impacting the calculation of the time requested to reach the positivity cutoff. [c] P-values of the Wald tests performed to verify the significance of group effects; na: not adjusted, ns: not significant, * $p < 0.05$, ** $p < 0.01$, *** $p < 0.001$. The group CD40.RBDv Conv. being considered as the group of reference, no adjustment for group effects have been performed on it. [d] The percentage of simulations for which the simulated parameters allowed to reach the positivity cutoff. CI, confidence interval; Q1, first quartile; Q3, third quartile.

Supplementary table III. Estimation of the four pooled monophasic-biphasic linear mixed models on anti-SARS-CoV-2 antibodies binding to Wuhan, Alpha, Beta and Delta RDB for the mRNA group, and prediction of the time required to reach the positivity cutoff of 200 AU/mL.

	Breakpoint (τ)<br><br>(days post-peak)<br>Mean [95% CI]<br>P-value[b]	Peak Value (β_0)<br><br>(log_{10} AU/mL)<br>Mean [95% CI]<br>P-value[b]	First Slope (β_1)<br><br>(log_{10} AU/mL/Week)<br>Mean [95% CI]<br>P-value[b]	Second Slope (β_2)<br><br>(log_{10} AU/mL/Week)<br>Mean [95% CI]<br>P-value[b]	Time to the positivity cutoff [a]<br><br>(years post-peak)<br>Median [Q1; Q3]<br>Cutoff reached (%)[c]
IgG binding to Wuhan RBD	47.97<br>[46.71 ; 49.22]<br>(na)	5.27<br>[5.18 ; 5.35]<br>(na)	-0.120<br>[-0.130 ; -0.109]<br>(na)	-0.050<br>[-0.053 ; -0.047]<br>(***)	0.96<br>[0.92 ; 0.99]<br>(100%)
IgG binding to Alpha RBD	48.06<br>[46.88; 49.24]<br>(na)	5.27<br>[5.18 ; 5.36]<br>(na)	-0.119<br>[-0.130 ; -0.108]<br>(na)	-0.050<br>[-0.057 ; -0.043]<br>(***)	0.96<br>[0.91 ; 1.01]<br>(100%)
IgG binding to Beta RBD	49.55<br>[47.63; 51.47]<br>(na)	5.06<br>[4.97 ; 5.14]<br>(na)	-0.123<br>[-0.133 ; -0.113]<br>(na)	-0.043<br>[-0.046 ; -0.039]<br>(***)	0.99<br>[0.95 ; 1.02]<br>(100%)
IgG binding to Delta RBD	52.44<br>[50.63; 54.26]<br>(na)	5.32<br>[5.23 ; 5.41]<br>(na)	-0.108<br>[-0.119 ; -0.097]<br>(na)	-0.050<br>[-0.059 ; -0.042]<br>(***)	0.99<br>[0.94 ; 1.05]<br>(100%)

[a] Threshold of 200 AU/mL. The median, Q1 and Q3 were extracted as the 50th, 25th, and 75th percentiles of the distribution of the time to positivity cutoff calculated on parameters simulated from the empirical posterior distribution. The reader can refer to the Supplementary Materials for more information [b] P-values of the Wald tests performed to verify the significance of group effects, compared to the group of reference CD40.RBDv Conv. ; na: not adjusted, ns: not significant, * $p < 0.05$, ** $p < 0.01$, *** $p < 0.001$. [c] The percentage of simulations for which the simulated parameters allowed to reach the positivity cutoff. CI, confidence interval; Q1, first quartile; Q3, third quartile.
